# Phylogenomics and Molecular Signatures for Species from the Plant Pathogen-Containing Order Xanthomonadales

**DOI:** 10.1371/journal.pone.0055216

**Published:** 2013-02-08

**Authors:** Hafiz Sohail Naushad, Radhey S. Gupta

**Affiliations:** Department of Biochemistry and Biomedical Sciences, McMaster University, Hamilton, Ontario, Canada; Université Claude Bernard - Lyon 1, France

## Abstract

The species from the order Xanthomonadales, which harbors many important plant pathogens and some human pathogens, are currently distinguished primarily on the basis of their branching in the 16S rRNA tree. No molecular or biochemical characteristic is known that is specific for these bacteria. Phylogenetic and comparative analyses were conducted on 26 sequenced Xanthomonadales genomes to delineate their branching order and to identify molecular signatures consisting of conserved signature indels (CSIs) in protein sequences that are specific for these bacteria. In a phylogenetic tree based upon sequences for 28 proteins, Xanthomonadales species formed a strongly supported clade with *Rhodanobacter* sp. 2APBS1 as its deepest branch. Comparative analyses of protein sequences have identified 13 CSIs in widely distributed proteins such as GlnRS, TypA, MscL, LysRS, LipA, Tgt, LpxA, TolQ, ParE, PolA and TyrB that are unique to all species/strains from this order, but not found in any other bacteria. Fifteen additional CSIs in proteins (viz. CoxD, DnaE, PolA, SucA, AsnB, RecA, PyrG, LigA, MutS and TrmD) are uniquely shared by different Xanthomonadales except *Rhodanobacter* and in a few cases by *Pseudoxanthomonas* species, providing further support for the deep branching of these two genera. Five other CSIs are commonly shared by Xanthomonadales and 1–3 species from the orders *Chromatiales, Methylococcales* and *Cardiobacteriales* suggesting that these deep branching orders of Gammaproteobacteria might be specifically related. Lastly, 7 CSIs in ValRS, CarB, PyrE, GlyS, RnhB, MinD and X001065 are commonly shared by Xanthomonadales and a limited number of Beta- or Gamma-proteobacteria. Our analysis indicates that these CSIs have likely originated independently and they are not due to lateral gene transfers. The Xanthomonadales-specific CSIs reported here provide novel molecular markers for the identification of these important plant and human pathogens and also as potential targets for development of drugs/agents that specifically target these bacteria.

## Introduction

The Xanthomonadales are gram-negative, non-spore forming, catalase-positive, aerobic, rod shape bacteria [1], which are part of the class Gammaproteobacteria [2]. This order is comprised of two families Xanthomonadaceae and Sinobacteraceae that contain 22 and 6 genera, respectively (http://www.bacterio.cict.fr/classifphyla.html#Proteobacteria). The *Xylella* and *Xanthomonas* species, which are part of the order Xanthomonadales, cause a wide variety of serious diseases in more than 400 agriculturally important plants. Some of the economically important crops that are affected by species from these two genera include tomato, cabbage, pepper, banana, citrus, rice, grapes, peach, plum, almond, coffee and maple [3]–[9] Additionally, *Xylella fastidiosa* is responsible for causing leaf scorch disease in many landscape and ornamental plants including oak, elm, mulberry, sycamore, maple and oleander [7], [9]–[11]. The diseases caused by these bacteria lead to major crop losses globally and thus they constitute serious agricultural and economic threat. In addition to these important phytopathogens, the Xanthomonadales also harbors the genus *Stenotrophomonas*, whose members (viz. *S. maltophila*) are multidrug resistant opportunistic pathogens, responsible for many hospital-acquired infections in immuno-compromised patients. These latter bacteria are also implicated in respiratory infections in cystic fibrosis patients [12]–[14].

The species from the order Xanthomonadales and its different families/genera are currently distinguished from other bacteria primarily on the basis of their branching in the 16S rRNA trees [1], [4], [15]. There is no biochemical, morphological or physiological characteristics known that are uniquely shared by various species from this order. Although Xanthomonadales are an order within the class Gammaproteobacteria, in phylogenetic trees based upon some genes/proteins sequences, these species are observed to branch with other classes of proteobacteria, particularly the Betaproteobacteria [16]–[20]. However, detailed phylogenetic studies based upon two independent, large datasets of concatenated protein sequences have now established that the species from the order Xanthomonadales are a deep branching clade within the class Gammaproteobacteria [21], [22]. Several recently identified molecular signatures that are uniquely shared by Xanthomonadales and all other Gammaproteobacteria also support the placement of this group within the Gammaproteobacteria [21], [23]. The anomalous branching of Xanthomonadales in some phylogenetic trees possibly results from the deep branching of Xanthomonadales within the Gammaproteobacteria and also in some cases by lateral gene transfers (LGTs). In particular, extensive work by Menck and coworkers indicate that about 25% of the genes in *Xanthomonas*, which include many genomic islands as well as some genes involved in the biosynthesis of NAD, arginine and cysteine, are acquired by LGTs [16], [24]–[28].

Because Xanthomonadales harbor many major phytopathogens and also some important human pathogens, it is important to understand the evolutionary relationships among these bacteria and identify molecular markers that are specific for either all Xanthomonadales or its different genera. Due to the importance of these bacteria for agriculture and human health, the complete genome sequences for 26 Xanthomonadales species/strains are now available in the NCBI database (see Table 1). In addition, genomes for many other species/strains from this order are currently being sequenced and partial sequence information for them is also available in the databases. These genomes provide valuable resource for discovering molecular and biochemical characteristics that are uniquely shared by these bacteria and which should provide novel means for their identification and also as potential new targets for development of drugs targeting these bacteria. Earlier comparative genomic studies on Xanthomonadales have focused on identifying characteristics that are responsible for the virulence and host specificity of different strains and pathovars of *Xanthomonas* and *Xylella* and on understanding the role of LGTs in their genome evolution [3], [4], [4], [7], [8], [11], [29]–[34], [34]–[36]. A recent study on DNA repair proteins also identified four conserved indels that were specific for the available Xanthomonadales species [28]. However, thus far no detailed study has been carried out which is aimed at identifying genetic or molecular characteristics that are uniquely shared by either all Xanthomonadales or its different genera.

**Table 1:** Sequence Characteristics of Xanthomonadales genomes.

Organism	GenBank Accession No.	Size(Mbp)	No. of Proteins	% GC content	Reference
*Stenotrophomonas maltophilia K279a*	AM743169.1	4.8	4386	66	[12]
*Stenotrophomonas maltophilia R551-3*	CP001111.1	4.6	4039	66	[12]
*Stenotrophomonas sp. SKA14*	ACDV00000000	4.9	4469	66	JCVI *
*Xanthomonas albilineans GPE PC73*	FP565176.1	3.7	3114	63	[32]
*Xanthomonas axonopodis pv. citri str. 306*	AE008923.1	5.3	4312	64	[56]
*Xanthomonas axonopodis pv. citrumelo FL 1195*	CP002914.1	5.0	4181	65	[55]
*Xanthomonas campestris pv. raphani strain 756C*	CP002789.1	4.9	4520	65	[33]
*Xanthomonas campestris pv. campestris str. 8004*	CP000050.1	5.1	4271	64	[30]
*Xanthomonas campestris pv. campestris str. ATCC 33913*	AE008922.1	5.1	4179	65	[56]
*Xanthomonas campestris pv. campestris str. B100*	AM920689.1	5.1	4466	65	[56]
*Xanthomonas campestris pv. vesicatoria str. 85-10*	AM039952.1	5.4	4487	64	[29]
*Xanthomonas vesicatoria ATCC 35937*	AEQV00000000	5.5	4927	65	[31]
*Xanthomonas gardneri ATCC 19865*	AEQX00000000	5.5	5027	65	[31]
*Xanthomonas oryzae pv. oryzicola BLS256*	CP003057.1	4.8	4474	64	[33]
*Xanthomonas oryzae pv. oryzae KACC10331*	AE013598.1	5.0	4064	63	[5]
*Xanthomonas oryzae pv. oryzae MAFF 311018*	AP008229.1	5.0	4372	63	NIAS*
*Xanthomonas oryzae pv. oryzae PXO99A*	CP000967.1	5.2	4988	63	[6]
*Xanthomonas perforans 91-118*	AEQW00000000	5.2	4637	63	[31]
*Xylella fastidiosa 9a5c*	AE003849.1	2.8	2766	52	[35]
*Xylella fastidiosa M23*	CP000941.1	2.5	2104	51	[10]
*Xylella fastidiosa M12*	CP001011.1	2.6	2161	51	[10]
*Xylella fastidiosa Temecula1*	AE009442.1	2.5	2034	51	[34]
*Xylella fastidiosa subsp. fastidiosa GB514*	CP002165	2.5	2216	51	[57]
*Pseudoxanthomonas spadix BD-a59*	CP003093.2	3.5	3149	67	[58]
*Pseudoxanthomonas suwonensis 11-1*	CP002446.1	3.4	3070	70	DOE-JGI*
*Rhodanobacter sp. 2APBS1*	AGIL00000000	4.0	3800	68	DOE-JGI*

* NIAS = Genome was sequenced by National Institute of Agrobiological Sciences, Japan.

* DOE-JGI = Genome was sequenced by DOE Joint Genome Institute USA.

* JCVI = Genome was sequenced by J. Craig Venter Institute, USA.

Using genome sequence data, our recent work has focused on identifying Conserved Signature Indels (inserts or deletions) (CSIs) of defined lengths that are present at specific locations in widely distributed proteins and which are uniquely found in particular groups of organisms [37]–[40]. The most parsimonious explanation of these CSIs is that they resulted from highly specific genetic changes that first occurred in a common ancestor of the particular groups of species and were then passed on to various descendants [37], [40], [41]. Further, depending upon the presence or absence of these CSIs in outgroup species, it is possible to infer whether a given CSI is an insert or a deletion and this information can be used to develop rooted phylogenetic relationships independently of phylogenetic trees [21], [37], [42]–[45]. Additionally, the shared presence of some CSIs in unrelated groups of bacteria can also identify possible cases of LGTs [46]. In this work, we report detailed phylogenetic and comparative analyses of protein sequences from Xanthomonadales genomes to identify CSIs that are specific for these organisms. These studies have identified 13 CSIs that are specific for all sequenced Xanthomonadales species and many others CSIs that provide information regarding evolutionary relationships among these bacteria. These molecular signatures provide novel and highly specific means for identification of Xanthomonadales species and for different types of studies on these bacteria. We also report here several CSIs that are commonly shared by Xanthomonadales and either Beta- and/or Alpha-proteobacteria. However, our analysis indicates that the shared presence of these CSIs in Xanthomonadales and these other bacterial groups is due to independent occurrence of similar genetic changes and not due to LGTs.

## Methods

### Phylogenetic Analyses

Phylogenetic analyses were conducted on a concatenated sequence alignment for 28 conserved and widely distributed proteins that have been widely used for phylogenetic studies [21], [47], [48] and are present in all the Xanthomonadales. These proteins included, alanyl-tRNA synthetase, arginyl-tRNA synthetase, cell division protein FtsY, chaperonin GroEL, dimethyladenosine transferase, DNA gyrase subunit A, DNA gyrase subunit B, DNA polymerase I, DNA-dependent helicase II, elongation factor Tu, histidyl-tRNA synthetase, isoleucyl-tRNA synthetase, methionyl-tRNA synthetase, molecular chaperone DnaK, O-sialoglycoprotein endopeptidase, phenylalanyl-tRNA synthetase subunit alpha, phosphatidate cytidylyltransferase, prolyl-tRNA synthetase, RpoB, RpoC, SecA, SecY, serine hydroxymethyltransferase, seryl-tRNA synthetase, signal recognition particle protein, thioredoxin reductase, tryptophanyl-tRNA synthetase and valyl-tRNA synthetase. For each of these proteins, sequences for all sequenced Xanthomonadales and a number of other Gamma-, Beta- and Alpha-proteobacteria were retrieved by Blastp searches and multiple sequence alignments were created by using the CLUSTAL_X 2.0 [49]. These sequence alignments were concatenated into a single large file and the poorly aligned regions from the alignment were removed using Gblocks 0.91 b program [50]. After removal of poor aligned regions, a total of 14621 aligned positions were present in the final dataset. A neighbor-joining (NJ) tree based on 100 bootstrap replicates was constructed using the JTT matrix-based method [51] in MEGA 5 [52]. A maximum-likelihood tree based upon the same sequence data set was also constructed using the Whelan and Goldman+Freq. model [53] using MEGA5. All positions containing gaps were not considered during these tree constructions.

### Identification of Xanthomonadales Specific Conserved Signature Indels (CSIs)

To search for signature sequences in different proteins that are specific for Xanthomonadales or for its subclades, Blastp searches were carried out on each proteins (open reading frame) from the genome of *Xylella fastidiosa* 9a5c against the NCBI nr database [35]. The results of blast searches were examined for high scoring homologs. For those proteins for whom high scoring homologs (E value <1e^−20^) were present in Xanthomonadales and several other bacteria, about 10–15 sequences representing different groups were retrieved and multiple sequence alignments were constructed using the CLUSTAL_X 2.0 program [49]. The sequence alignments were visually inspected to identify any conserved indels that were restricted to Xanthomonadales and which were flanked on both sides by at least 5–6 identical/conserved residues in the neighboring 30–50 amino acids [21], [40], [54]. The conserved indels, which in addition to Xanthomonadales were also present in few other species, were also retained. The indels that were not flanked on both sides by conserved regions were not further evaluated as they do not provide useful molecular markers [23], [37], [40]. The species distribution of all indels thus identified (∼150) was further examined by detailed Blastp searches against the nr database (500 top hits) on short sequence segments containing the indels and their flanking conserved regions. Based upon detailed Blast searches, many original indels queries were found to be uninformative for this study due to a variety of reasons including their presence in only a single species/strain, lack of sequence conservation, presence of other confounding indels in the same area in other species, lack of specificity of the indels for any particular group and large variation in their lengths, etc. Hence, such indels were not further studied. However, for different indels those were specific for Xanthomonadales or present in a limited number of other bacteria, sequence information for them were compiled into signature files that are shown here. Due to space considerations, the signature files shown here contain information for only a limited number of species from other bacteria such as Alpha, Beta and Gammaproteobacteria and different strains of the same species are also not shown. However, unless otherwise noted, all of these CSIs are specific for the indicated groups and they are also present in different strains of the Xanthomonadaceae species for which sequence information is available (Table 1).

## Results

### Phylogenetic Analysis of Genome Sequenced Xanthomonadales

The genome sequences are now available for 26 Xanthomonadales including 15 for *Xanthomonas* species/strains [5], [6], [29]–[33], [55], [56], 5 for different strains/pathovars of *Xylella fastidiosa* [10], [34], [35], [57], 3 for *Stenotrophomonas* species/strains [12], 2 for *Pseudoxanthomonas* species [58] and for the *Rhodanobacter* sp. 2APBS1. Some characteristics of these genomes are listed in Table 1. Their genome sizes varied from 2.5 Mb to 5.3 Mb and the xylem-inhabiting bacterium *Xylella fastidiosa* had the smallest or most reduced genome. Further, in contrast to other Xanthomonadales species/strains whose mol G+C % was in the range of 61–67%, the *Xylella* strains/pathovars have much lower G+C content. The reduced genome size and the lower G+C mole content of *Xylella* strains/pathovars have likely resulted from their adaptation to the more stable xylem environment [7].

**Figure 1:**
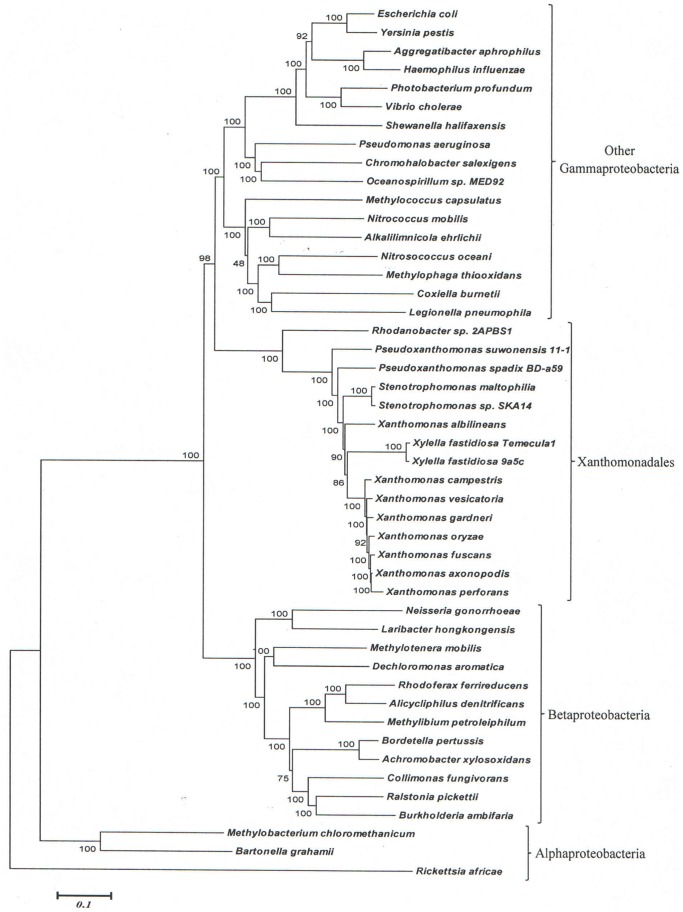
Phylogenetic tree for Xanthomonadales based on concatenated sequences for 28 conserved proteins. The tree shown is a NJ distance tree, however, similar branching was observed in the ML tree (Figure S1). The observed bootstrap scores for various nodes are shown on the branch points. The tree was rooted using sequences from Alphaproteobacteria.

The sequence information from these genomes was also used to examine the evolutionary relationships among the sequenced Xanthomonadales species. Detailed phylogenetic studies on Gammaproteobacteria and other proteobacteria based upon concatenated sequences for different large datasets of protein sequences have been reported previously [19], [21], [22]. In these trees [4], [28], [59], species from the order Xanthomonadales formed a monophyletic clade and one of the deepest branching lineages within the Gammaproteobacteria [21], [22]. Hence, in the present work, phylogenetic trees based upon concatenated sequences were mainly constructed to clarify the branching order of species within the order Xanthomonadales. The dataset employed in this study included sequence information for only a limited number of other proteobacteria. Figure 1 shows a NJ distance tree based upon concatenated protein sequences, which was rooted using sequences from Alphaproteobacteria. The branching order of various Xanthomonadales species in the ML tree (Figure S1) is very similar to that seen in the NJ tree. In both ML and NJ tree, the Xanthomonadales species formed a strongly supported clade branching within the other Gammaproteobacteria. This clade was separated from all other Gammaproteobacteria by a long branch. Similar monophyletic grouping and branching of the Xanthomonadales species within the Gammaproteobacteria have been observed in earlier studies [19], [21], [22]. Among the sequenced Xanthomonadales species, *Rhodanobacter* was found to be the deepest branching species and it was separated from all other Xanthomonadales by a long branch. Interestingly, the sequenced *Xanthomonas* species showed polyphyletic branching in the tree, with *X. albilineans* branching deeply and separately from the other *Xanthomonas* species (Figure 1 and Figure S1). The tree shown in Figure 1 provides a phylogenetic framework for understanding and interpreting the significance of various CSIs observed in this work.

### Identification of Conserved Signature Indels that are Specific for Xanthomonadales

**Figure 2:**
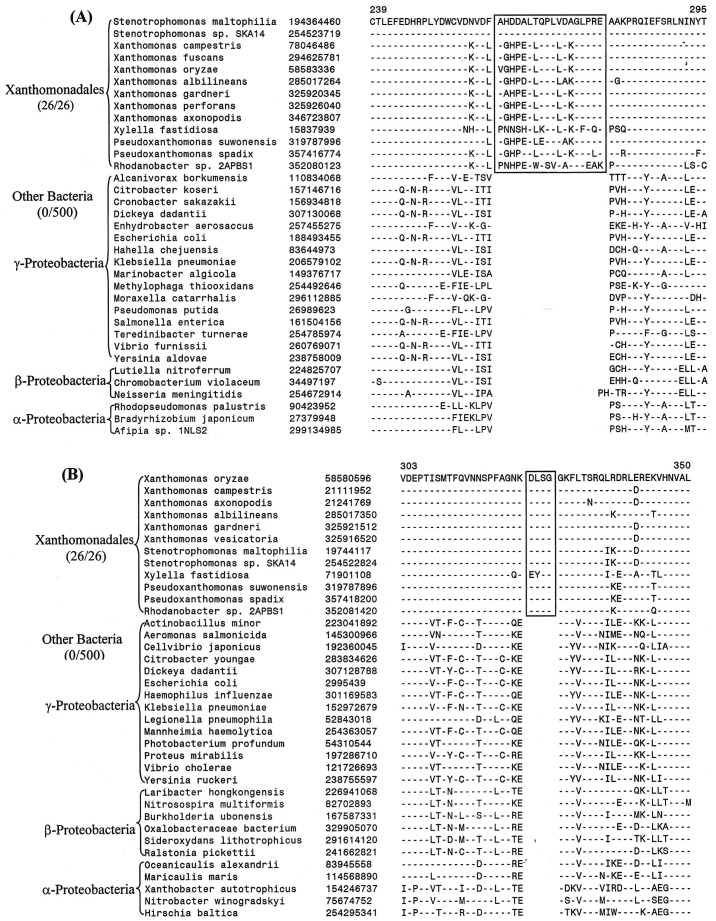
Examples of conserved signature indels (CSIs) that are specific for the order Xanthomonadales. Excerpts are shown from the sequence alignments of (A) Glutaminyl t-RNA synthetase and (B) GTP-binding elongation factor proteins showing two CSIs that are uniquely found in various sequenced Xanthomonadales species, but not found in any other bacteria. Information for other CSIs that are specific for the Xanthomonadales is provided in Figures S2–S12 and Table 2. The dashes in these as well as all other alignments show identity with the amino acid on the top line. The Gene bank identification numbers of various sequences are shown in the second column and the numbers on the top indicate the position of this sequence in the species shown on the top line. The sequence information is shown here for only representative species. However, unless otherwise indicated, these CSIs are highly specific for the indicated group of species.

Our work has identified 13 CSIs that are uniquely present in all sequenced Xanthomonadales including the deepest branching *Rhodanobacter*. Two examples of these CSIs are shown in Figure 2A & B. In the first case (Figure 2A), an 18 aa insert in highly conserved region of the protein glutaminyl-tRNA synthetase, which plays an essential role in protein synthesis by linking glutamine to its cognate tRNA [60]. The large insert in GlnRS is uniquely shared by all available sequences from Xanthomonadales species but not found in any other bacteria (at least the top 500 blast hits). In the other example shown here (Figure 2B), a 4 aa insert in a GTP-binding elongation factor protein (typA) is commonly shared by all sequenced Xanthomonadales, but again it is not found in any other bacteria. Both these CSIs are present in highly conserved regions of the proteins and their sequences are also highly conserved. Because these CSIs are lacking in other bacteria, they constitute inserts in the Xanthomonadales rather than deletions in other bacteria [38]. The sequence information for other CSIs that are uniquely present in all sequenced species/strains of Xanthomonadales is presented in Figures S2–S12 and a summary of their characteristics is provided in Table 2 (first 13 entries). These CSIs include a 7 aa insert in amino acid/peptide transported protein; 5 aa insert in conserved region of the large-conductance mechanosensitive channel protein; a 3 aa insert in LysRS; 2 aa insert in highly conserved region of the protein lipoyl synthase (LipA); 1 aa inserts in the proteins Tgt, LpxA and TolQ; a 13 aa deletion in alpha-2-macroglobulin domain-containing protein and 1 aa deletions in the ParE, PolA and TyrB proteins. Because these CSIs are present in all sequenced Xanthomonadales but not found in any other bacteria, the most likely explanation is that genetic changes responsible for them first occurred in a common ancestor of the Xanthomonadales and then passed on to various descendants by vertical descent.

**Table 2:** Conserved Signatures Indels that are specific for Xanthomonadales.

Protein Name	Gene Name	GenBankIdentifier	Figure No	Indel Size	Indel Position^a^	Exceptions^b^
Glutaminyl-tRNA synthetase	glnS	194364460	Figure 2A	18 aa ins	239–295	None
GTP-binding elongation factor protein	typA	58580596	Figure. 2B	4 aa ins	303–350	None
Amino acid/peptide transporter	–	71275790	Figure S2	7 aa ins	164–213	None
Large-conductance mechanosensitive channel	mscL	294667079	Figure S3	5 aa ins	40–85	None
Lysyl-tRNA synthetase	lysS	194365604	Figure S4	3 aa ins	34–85	None
Lipoyl synthase	lipA	58583575	Figure S5	2 aa ins	156–209	None
Queuine tRNA-ribosyltransferase	tgt	194365393	Figure S6	1 aa ins	289–339	None
Acyl-(acyl-carrier-protein)–UDP-N-acetyl-glucosamine O-acyltransferase	lpxA	71275623	Figure S7	1 aa ins	164–210	None
TolQ protein	tolQ	21232451	Figure S8	1 aa ins	177–217	None
Alpha-2-macroglobulin domain-containing protein	–	194366795	Figure S9	13 aa del	607–661	None
DNA topoisomerase IV subunit B	parE	84624476	Figure S10	1 aa del	282–326	None
DNA polymerase I	polA	194367713	Figure S11	1 aa del	28–65	None
Aromatic amino acid aminotransferase	tyrB	28197970	Figure S12	1 aa del	306–354	None
Glutaminyl-tRNA synthetase	glnS	194364460	Figure 3	1 aa del	77–131	*Methylobacter tundripaludum, Methylomicrobium album BG8,Dichelobacter nodosus*
DNA polymerase III subunit beta	RpoB	194363780	Figure S13	1 aa del	44–81	*Marinomonas* sp. MWYL1, *Thioalkalivibrio sp. HL-EbGR7*
Lipid-A-disaccharide synthase	lpxB	190573490	Figure S14	2 aa ins	317–358	*Cardiobacterium hominis, Allochromatium vinosum, Alteromonadales bacterium*
Carbamoyl phosphate synthase large subunit	carB	166711938	Figure S15	1 aa ins	403–457	*Marinobacter sp. ELB17*
Putative secreted protein	–	188992701	Figure S16	1 aa ins	1285–1318	*Teredinibacter turnerae*
Aminopeptidase P	pepP	294627124	Figure S17	1 aa del	211–246	*Thioalkalivibrio sp. HL-EbGR7,Alkalilimnicola ehrlichii*

a The indel position provided indicates the region of the protein containing the CSI.

b For details go to respective figures.

**Figure 3:**
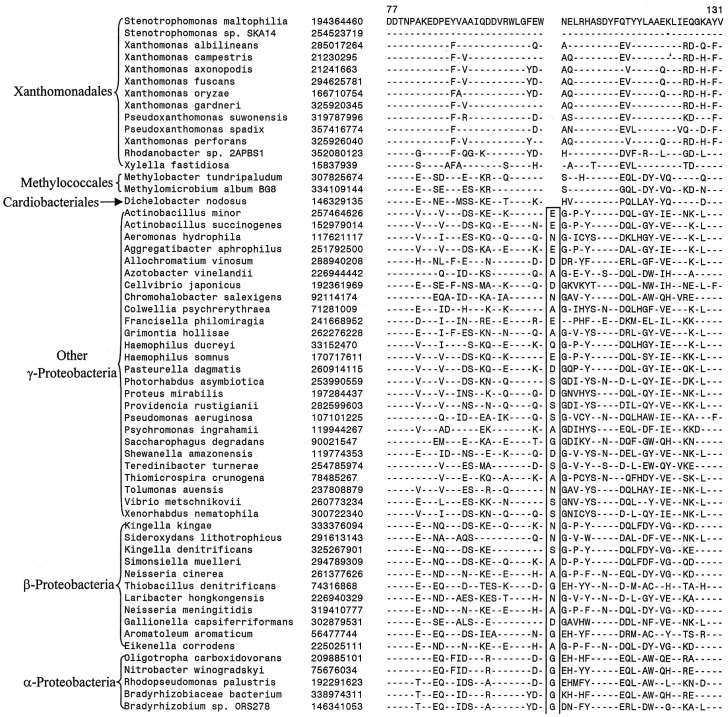
Partial sequence alignment of glutaminyl t-RNA synthetase showing a CSI that is specifically present in various sequenced Xanthomonadales and some other Gammaproteobacteria. This CSI as well as a few other CSIs identified in this work (see Table 2 and Figures S13–S17) suggest a possible relationship of Xanthomonadales to these deep branching orders of Gammaproteobacteria.

In addition to these CSIs that are uniquely found in all Xanthomonadales, we have also come across 6 other CSIs, where in addition to the Xanthomonadales, the identified CSIs are also present in 1–3 other Gammaproteobacteria. These species are generally from some of the other deep branching orders of Gammaproteobacteria such as *Chromatiales, Methylococcales* and *Cardiobacteriales*, which branch in the proximity of Xanthomonadales [21], [22], [28]. One example of such a CSI consisting of a 1 aa deletion in a conserved region of the protein glutaminyl-tRNA synthetase that is commonly shared by various Xanthomonadales and also by a few *Methylococcales* and *Cardiobacteriales* species is presented in Figure 3. Sequence information for others CSIs of this kind is presented in Figures S13–S17 and in Table 2 (last six records). Cutino-Jimenez et al. [28]also reported a CSI in Topoisomerase I that was commonly shared by various *Xanthomonadales*, *Methylococcales*, *Cardiobacteriales, Chromatiales, Legionellales* and *Thiotrichales.* The information provided by these CSIs could prove useful in establishing a specific relationship of the Xanthomonadales to these other deep branching orders of Gammaproteobacteria.

### CSIs Supporting the Deeper Branching of *Rhodanobacter* within the Xanthomonadales

**Figure 4:**
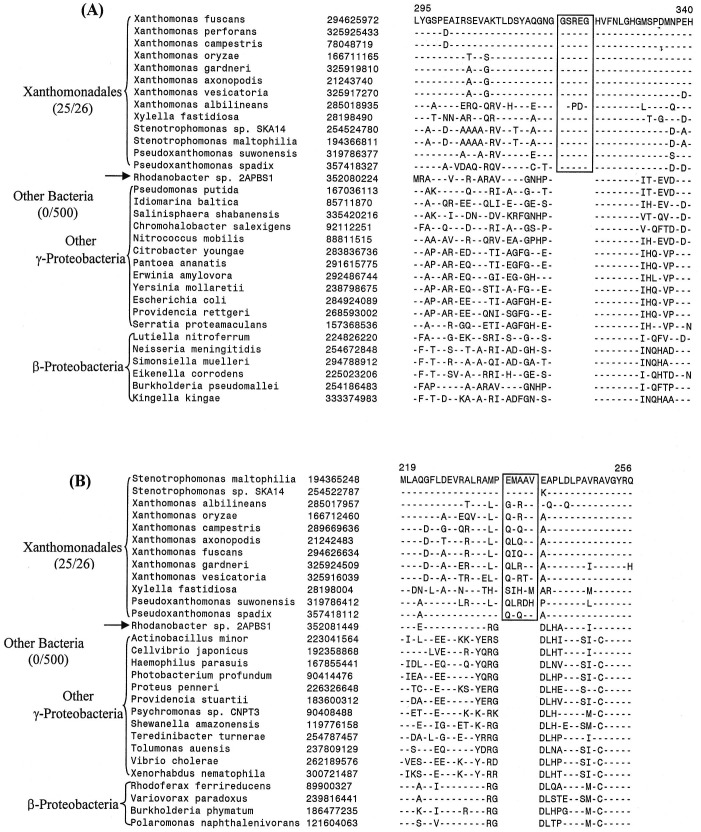
Examples of CSIs those are present in various Xanthomonadales species except *Rhodanobacter* sp. 2APBS1. Excerpts are shown from the sequence alignments of (A) uroporphyrinogen decarboxylase (HemE) and (B) tRNA delta(2)-isopentenylpyrophosphate transferase (MiaA) proteins showing two conserved signature indels (boxed) that are specifically found in various sequenced Xanthomonadales species, except *Rhodanobacter* sp. 2APBS1. These CSIs were likely introduced in these genes in a common ancestor of the Xanthomonadales after branching of *Rhodanobacter*. Information for CSIs in other proteins showing similar species specificities is provided in Figures S18–S30 and Table 3.

In the phylogenetic tree shown in Figure 1 and Figure S1, *Rhodanobacter* sp. 2APBS1 exhibited the deepest branching amongst the sequenced Xanthomonadales. During our analyses, we have found 15 CSIs that are uniquely shared by all other Xanthomonadales except *Rhodanobacter*, supporting the deeper branching of this species in comparison to other Xanthomonadales. Two examples of such CSIs that are uniquely found in different Xanthomonadales, but not in *Rhodanobacter* are shown in Figure 4A & B. In both these cases 5 aa inserts in highly conserved regions of the proteins uroporphyrinogen decarboxylase (Figure 4A) and in the protein tRNA delta(2)-isopentenylpyrophosphate transferase (Figure 4B) are uniquely shared by different sequenced Xanthomonadales except *Rhodanobacter*. These CSIs are not present in any other bacteria. A summary of the characteristics of different CSIs showing this type of species distribution pattern is presented in Table 3 and the sequence alignments of the corresponding proteins are provided as Figures S18–S30. The proteins in which these CSIs are found include protoheme IX farnesyltransferase (CoxD), DNA polymerase III alpha subunit (DnaE), DEAD box helicase domain-containing protein, ribose-5-phosphate isomerase A (RpiA), DNA polymerase I (PolA), glucose-6-phosphate 1-dehydrogenase (Zwf1), AspRS, 2-oxoglutarate-dehydrogenase E1 component (SucA), coproporphyrinogen III oxidase (CpoX), and TrmD. In a few of these cases, the CSIs under consideration was also not found in one or both of the *Pseudoxanthomonas* species, supporting their deeper branching in comparison to other Xanthomonadales genera (viz. *Xylella, Xanthomonas* and *Stenotrophomonas*) (Figures S32–S34). In a recent study, Cutino-Jimenez et al. [28] had reported four CSIs in DNA repair proteins that were indicated to be specific for Xanthomonadales. Our analyses of these CSIs, which were also identified in our work, indicate that they are lacking in either *Rhodanobacter* (4 aa insert in DnaE and 1 aa insert in RecA) or both *Rhodanobacter* and in *P. suwonensis* (5 aa insert in MutS and >50 aa insert in LigA) (Figures S29–S31 and S35). The information for these CSIs is also summarized in Table 3. Based upon the species distribution of these CSIs and the branching positions of *Rhodanobacter* (and *Pseudoxanthomonas*) in phylogenetic trees, the genetic changes responsible for these CSIs likely occurred in common ancestors of other Xanthomonadales species after the divergence of *Rhodanobacter* sp. 2APBS1 and also in some cases that of *Pseudoxanthomonas* species.

**Table 3:** CSIs that are specific for Xanthomonadales except *Rhodanobacter* sp. 2APBS1.

Protein Name	Gene Name	GenBankIdentifier	Figure No	Indel Size	Indel Position^a^	Specificity within Xanthomonadales
Uroporphyrinogen decarboxylase	hemE	294625972	Figure 4A	5 aa ins	295–340	All except *Rhodanobacter* sp. 2APBS1
tRNA delta(2)-isopentenylpyrophosphate transferase	miaA	194365248	Figure 4B	5 aa ins	219–256	All except *Rhodanobacter* sp. 2APBS1
Protoheme IX farnesyltransferase	coxD	15837961	Figure S18	4 aa ins	150–192	All except *Rhodanobacter* sp. 2APBS1
DNA polymerase III subunit alpha	dnaE	21242159	Figure S19	1 aa ins	583–638	All except *Rhodanobacter* sp. 2APBS1
DEAD box helicase domain-containing protein	–	194364258	Figure S20	1 aa ins	155–200	All except *Rhodanobacter* sp. 2APBS1
Ribose-5-phosphate isomerase A	rpiA	194367055	Figure S21	1 aa ins	127–169	All except *Rhodanobacter* sp. 2APBS1
DNA polymerase I	polA	21244827	Figure S22	1 aa ins	136–180	All except *Rhodanobacter* sp. 2APBS1
Aspartyl-tRNA synthetase	aspS	194366904	Figure S23	4 aa del	343–391	All except *Rhodanobacter* sp. 2APBS1
2-oxoglutarate-dehydrogenase E1 component	sucA	194366403	Figure S24	1 aa del	782–830	All except *Rhodanobacter* sp. 2APBS1
Coproporphyrinogen III oxidase	cpoX	194367710	Figure S25	1 aa del	166–215	All except *Rhodanobacter* sp. 2APBS1
2-oxoglutarate-dehydrogenase E1 component	sucA	194366403	Figure S26	1 aa del	106–164	All except *Rhodanobacter* sp. 2APBS1
Asparagine synthase b protein	asnB	285018780	Figure S27	4–5 aa ins	404–445	All except *Rhodanobacter* sp. 2APBS1
Asparagine synthase b protein	asnB	194365058	Figure S28	1–2 aa ins	96–132	All except *Rhodanobacter* sp. 2APBS1
DNA polymerase III subunit alpha b	dnaE	77747494	Figure S29	4 aa ins	522–576	All except *Rhodanobacter* sp. 2APBS1
DNA repair protein RecA^b^	recA	15836728	Figure S30	2 aa ins	172–238	All except *Rhodanobacter* sp. 2APBS1
5′-nucleotidase	–	21231001	Figure 5A	11–13 aa ins	123–188	All except *Rhodanobacter* sp. 2APBS1& *Pseudoxanthomonas suwonensis*
CTP synthetase	pyrG	194365226	Figure 5B	2 aa ins	253–291	All except *Rhodanobacter* sp. 2APBS1 *Pseudoxanthomonas suwonensis & Pseudoxanthomonas suwonensis*
DNA mismatch repair protein MutS b	mutS	15838317	Figure S31	5 aa ins	765–806	All except *Rhodanobacter* sp. 2APBS1 & *Pseudoxanthomonas suwonensis*
DNA polymerase III subunit alpha	dnaE	194365029	Figure S32	2 aa del	65–120	All except *Rhodanobacter* sp. 2APBS1 & *Pseudoxanthomonas suwonensis*
tRNA (guanine-N(1)-)-methyltransferase	trmD	194364933	Figure S33	2 aa ins	140–200	All except *Rhodanobacter* sp. 2APBS1 & *Pseudoxanthomonas suwonensis*
Glucose-6-phosphate 1-dehydrogenase	zwf	190573773	Figure S34	4 aa del	290–334	All except *Rhodanobacter* sp. 2APBS1 & *Pseudoxanthomonas spadix BD-a59*
DNA ligase NAD dependent b	ligA	77747612	Figure S35	57–65 aa ins	461–583	All except *Rhodanobacter* sp. 2APBS1 & *Pseudoxanthomonas suwonensis*

a The indel position provided indicates the region of the protein containing the CSI.

b These CSIs have been previously described [28].

**Figure 5:**
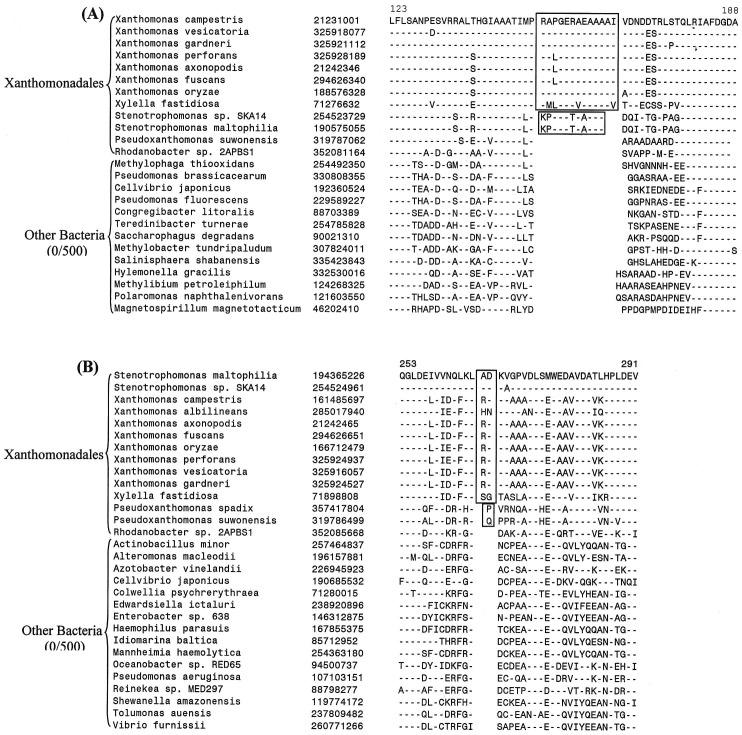
Example of CSIs those are able to distinguish two different clades of Xanthomonadales. Partial sequence alignments are shown of the proteins (A) 5′-nucleotidase and (B) CTP synthetase showing two CSI, which due to their different lengths are able to distinguish between two different clades of Xanthomonadales. In (A), a 13 aa insert is present in all of the *Xanthomonas* and *Xylella* species, whereas the two *Stenotrophomonas* spp. contain an 11 aa insert in this position. Similarly, in (B), all of the *Xanthomonas*, *Xylella* and *Stenotrophomonas* species have a 2 aa insert, whereas the two Pseudoxanthomonas spp. contain a 1 aa insert in this position. Different possibilities to account for these CSIs are discussed in the text.

In addition to the CSIs discussed above 4 other proteins contains CSIs of different lengths at the same position, which are uniquely shared by all sequenced species/strains of Xanthomonadales except *Rhodanobacter* sp. 2APBS1 and in some cases *Pseudoxanthomonas.* However, these CSIs due to differences in their lengths are also able to distinguish between different genera of *Xanthomonadaceae*. Two examples of such CSIs are presented in Figure 5. In the first case in the protein 5′-nucleotidase (Figure 5A), which catalyzes the hydrolysis of nucleotides to nucleosides, a 13 aa insert is uniquely shared by all *Xanthomonas* and *Xylella* species, whereas the two *Stenotrophomonas* species have an 11 aa insert in the same position. Because both these CSIs are present at the same position and they are related in sequences, the most likely explanation about their occurrence is that a 13 aa insert was initially introduced in a common ancestor of the *Xanthomonas, Xylella* and *Stenotrophomonas* genera and it was followed by a 2 aa deletion in the genus *Stenotrophomonas.* Alternatively, an 11 aa insert was initially introduced in a common ancestor of these three genera followed by another 2 aa insert in a common ancestor of the *Xanthomonas* and *Xylella* genera. Likewise, in a conserved region of the asparagine synthase b protein (AsnB), a 5 aa insert is present in various *Xylella, Xanthomonas* and *Pseudoxanthomonas*, whereas the two *Stenotrophomonas* species have a smaller insert (4 aa) in this position (Figure S27). The AsnB protein also contains another CSI in a different position (see Figure S28), where a 1 aa insert is present in *Xylella, Xanthomonas* and *Pseudoxanthomonas*, species, whereas the two *Stenotrophomonas* species have a 2 aa insert in the same position. In another example of this kind, in the protein CTP synthetase, a 2 aa insert in a conserved region is uniquely shared by various *Xylella, Xanthomonas* and *Stenotrophomonas* species/strains, whereas the two *Pseudoxanthomonas* species contain a 1 aa insert in this position (Figure 5B). These CSIs, in addition to supporting the deeper branching of *Rhodanobacter* in comparison to other Xanthomonadales, also serve to differentiate *Stenotrophomonas* and *Pseudoxanthomonas* species from other genera of *Xanthomonadaceae*.

### CSIs that are Commonly Shared by Xanthomonadales and Some Alpha- and Beta-proteobacteria

**Figure 6:**
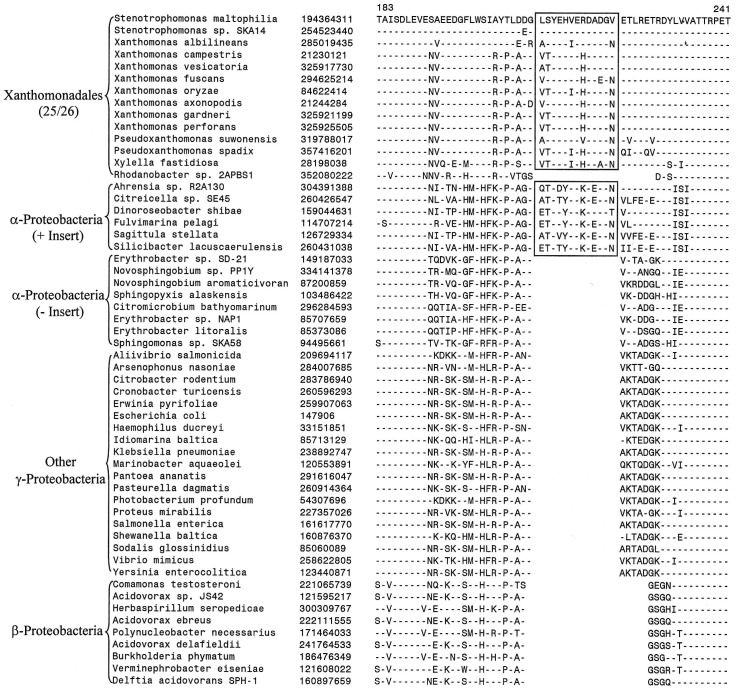
Partial sequence alignments of valyl t-RNA synthetase showing a 13 aa insert that is commonly shared by various Xanthomonadales and a subgroup of Alphaproteobacteria. Other Alpha- and Gamma-proteobacteria do not contain this insert.

**Figure 7:**
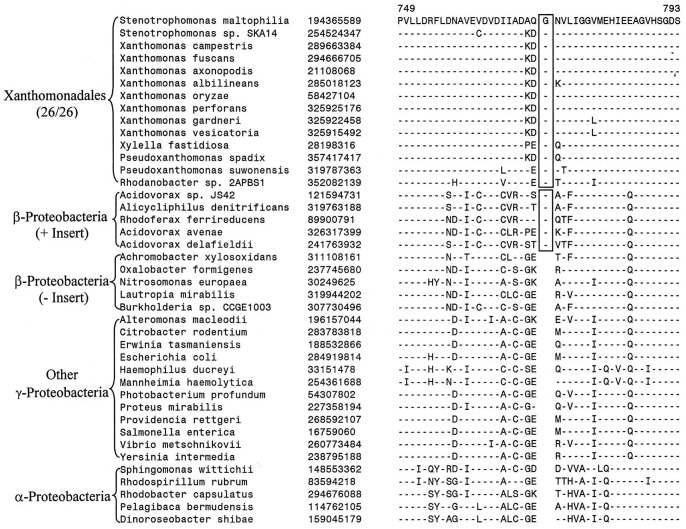
Partial sequence alignment of carbamoyl phosphate synthase showing a 1 aa insert that is commonly shared by Xanthomonadales and a subgroup of Betaproteobacteria. The distinct branching of these two groups in a phylogenetic tree based upon CarB sequence (Figure S36) provides evidence that this shared CSIs is not a result of LGT.

In addition to the above proteins that contained CSIs, which were highly specific for Xanthomonadales species (or 1–2 closely related species), our analyses have also identified 7 other CSIs, which in addition to various Xanthomonadales are also shared by some Betaproteobacteria and/or Alphaproteobacteria. Two examples of these CSIs are shown in Figures 6 and 7. In the protein valyl-tRNA synthetase, which plays an essential role in protein synthesis, a 13 aa insert in a highly conserved region is present in all sequenced Xanthomonadales, except *Rhodanobacter* (Figure 6). Interestingly, a very similar CSI is also present in several species belonging to the class Alphaproteobacteria (e.g. *Ahrensia sp. R2A130, Labrenzia alexandrii, Rhodobacter capsulatus, Sagittula stellata* etc.) whereas other Alphaproteobacteria do not contain this insert. In the other example shown here (Figure 7), in the protein carbamoyl phosphate synthase large subunit (CarB), a 1 aa insert in a conserved region is commonly shared by various Xanthomonadales and a subgroup of Betaproteobacteria (mainly *Burkholderiales*), but not by any other bacterial groups. The shared presence of similar CSIs by different Xanthomonadales and species from these other classes of proteobacteria could result from a variety of possibilities including lateral transfers of genes for these proteins between these two groups of bacteria or alternatively by independent occurrence of similar genetic changes in these lineages.

**Figure 8:**
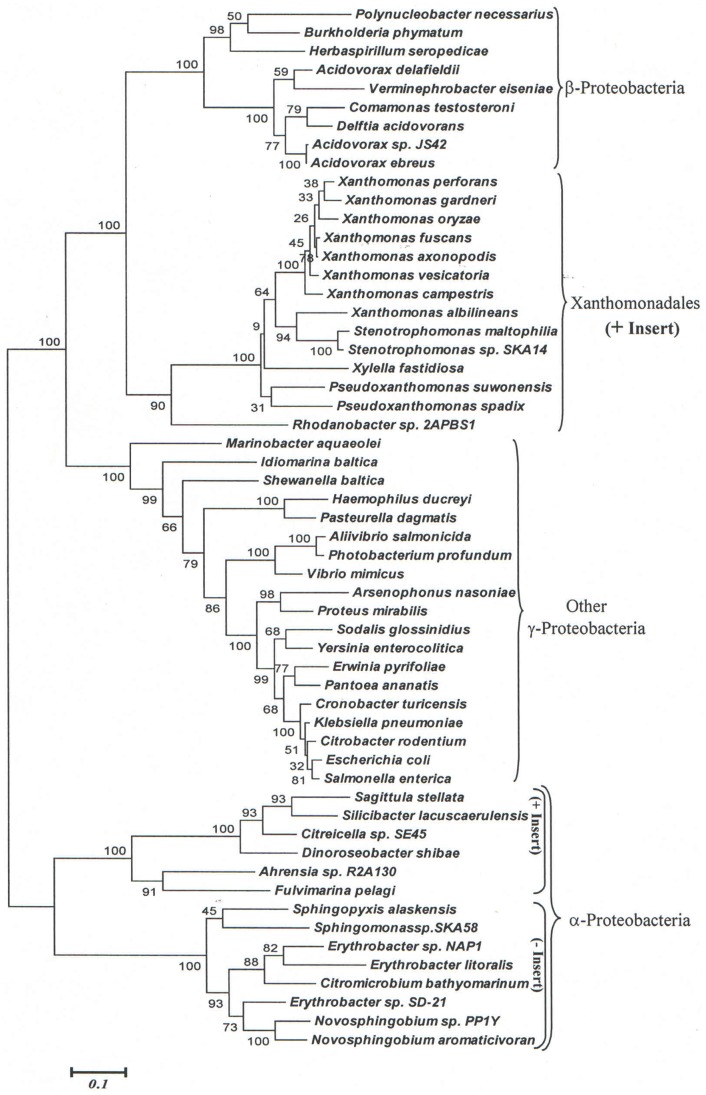
Phylogenetic tree based upon valyl t-RNA synthetase sequences. The distinct branching of Xanthomonadales and the Alphaproteobacteria containing this insert suggests that the shared presence of this CSIs in these two groups is not due to a LGT.

To distinguish between these possibilities, phylogenetic trees for the ValRS and CarB sequences for the same species as shown in Figures 6 and Figure 7 were constructed. In the tree based upon ValRS sequences, which is shown Figure 8, all of the Alphaproteobacteria species (both containing and lacking the insert) formed a strongly supported clade that branched distinctly from the Xanthomonadales. The Xanthomonadales species in this tree branched in between the clades consisting of Betaproteobacteria and the other Gammaproteobacteria, but that is not surprising in view of phylogenetic position within the Gammaproteobacteria. If the shared presence of the CSI in the Xanthomonadales and the CSI-containing Alphaproteobacteria was due to LGTs, then the Alphaproteobacteria containing this CSI should have branched with the Xanthomonadales, which is not observed here. Similarly, in the tree based upon CarB sequences (Figure S36), all of the Betaproteobacteria branched together and no association was observed between the insert containing Betaproteobacteria and the Xanthomonadales. These results do not support the possibility that LGT was responsible for the shared presence of CSIs in these two groups. Instead in the phylogenetic trees shown in Figure 8 and Figure S36, the clades comprising of the inserts containing Alphaproteobacteria or Betaproteobacteria formed distinct subclades within the rest of the Alpha- or Beta-proteobacteria. Thus, it is likely that the genetic changes responsible for these CSI occurred independently in the common ancestors of these subclades of species.

Besides these two proteins that contained CSIs, which were commonly shared by Xanthomonadales and either some Alpha- or Beta-proteobacteria, five other proteins were identified that contained CSIs showing similar species distributions. These included: two CSIs consisting of 1 aa conserved deletions in a hypothetical protein XOO1065 and the protein orotate phosphoribosyltransferase (PyrE) that are commonly shared by various Xanthomonadales and some Betaproteobacteria (Figures S37 and S38); two CSIs consisting of 1 aa and 2 aa inserts in the proteins putative ribonuclease HII (RnhB) and glycyl-tRNA synthetase subunit beta (GlyS) that are also commonly shared by various Xanthomonadales and some Betaproteobacteria (Figures S39 and S40); a 1 aa deletion in a conserved region in the septum site-determining protein MinD that is commonly shared by Xanthomonadales and some Alpha- and Beta-proteobacteria (Figure S41). The phylogenetic trees based upon the sequences of these proteins are shown in Figures S42 to S46. In all of these trees, the proteobacterial groups which contained similar CSIs as found in the Xanthomonadales did not branch with the Xanthomonadales. These results provide evidence that the CSIs in these other bacterial groups have originated independently and their shared presence is not due to LGTs from Xanthomonadales.

## Discussion

**Figure 9:**
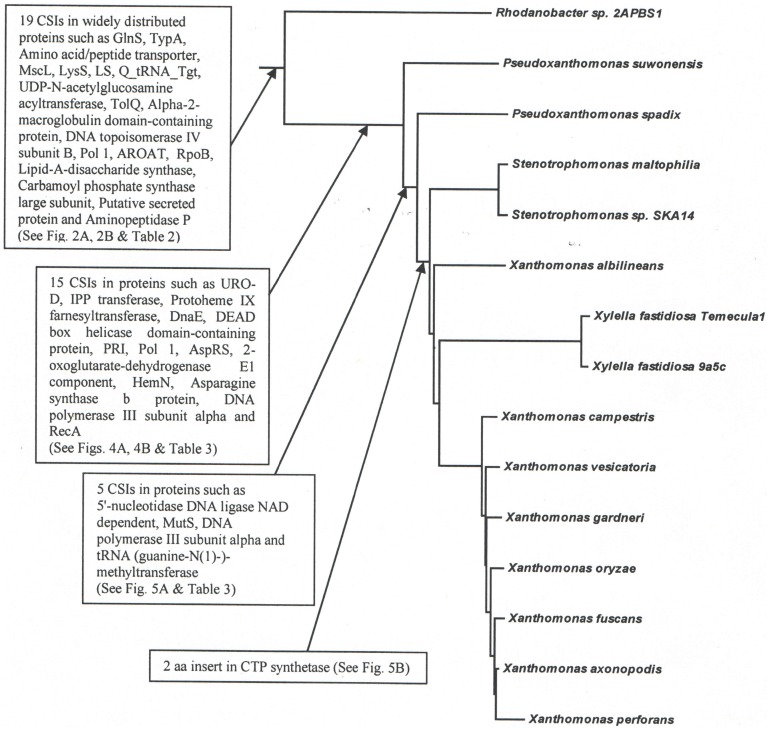
A summary diagram showing the species specificity of various CSIs identified in this work and the evolutionary stages where the genetic changes responsible for them were likely introduced.

The Xanthomonadales species harbor many major plant pathogens [3], [4], [9] as well as some important human pathogens. However, these bacteria are presently distinguished from other bacteria solely on the basis of their branching in phylogenetic trees (primarily 16S rRNA) and no molecular or biochemical characteristic that is uniquely shared by various species from this group of bacteria is currently known [1]. This paper reports detailed phylogenetic and comparative genomic analyses of sequenced Xanthomonadales species to identify molecular markers that are specific for these bacteria and which are also helpful in understanding their evolutionary relationships. We report here for the first time 13 molecular signatures consisting of conserved indels in widely distributed proteins that are distinctive characteristics of all sequenced Xanthomonadales species, but they are not found in any other bacteria. In view of their Xanthomonadales-specificity, the most parsimonious explanation to account for these CSIs is that the rare genetic changes responsible for them occurred only once in a common ancestor of the Xanthomonadales and were then passed on to various descendent species vertically as shown in Figure 9 [37], [54], [61]. Further, the absence of these CSIs in all other bacteria strongly indicates that the genes for these proteins have not been laterally transferred from Xanthomonadales to other bacterial groups or vice versa. Thus, these molecular signatures (or synapomorphies) provide novel means for the identification and circumscription of species from the order Xanthomonadales in clear molecular terms.

We also report in this work detailed phylogenetic analyses of (sequenced) Xanthomonadales species based upon concatenated sequences for 28 widely distributed proteins. Earlier phylogenetic studies on Xanthomonadales are mainly based upon 16S rRNA or single genes such as Gyrase B and most of them cover only the genus *Xanthomonas* [4], [15], [59], [62], [63]. Among a number of novel relationships seen in this tree, these trees showed that *Rhodanobacter* sp. 2APBS1 formed the deepest branch within the Xanthomonadales and it was separated from all other species by a long branch. The branching of *Pseudoxanthomonas* and then other *Xanthomonadaceae* genera followed it. Importantly, our analyses have also identified 15 CSIs that are uniquely present in all other Xanthomonadales, except *Rhodanobacter* and in a few cases also by the *Pseudoxanthomonas* species. The genetic changes responsible for theses CSIs were likely introduced in a common ancestor of the other Xanthomonadales after the branching of *Rhodanobacter* and also in some cases *Pseudoxanthomonas* (Figure 9) and they provide independent evidence for the deep branching of these lineages with respect to other genera within this order.

Xanthomonadales species are indicated to have undergone extensive LGTs with other prokaryotic taxa particularly Alpha, Beta and some orders of Gamma- proteobacteria and in some cases with Archaea as well [16], [24]–[27]. In the present work, we have also identified several examples where a given CSI, in addition to being shared by all or most Xanthomonadales, was also present in some species from other groups of bacteria, most commonly from Alpha-, Beta- and Gamma- proteobacteria. Of these CSIs, five were present only in 1–3 species from other deep branching orders of Gammaproteobacteria and their possible significance is discussed below. Seven other CSIs were commonly shared by various Xanthomonadales and also several Betaproteobacteria and/or both Alpha- and Beta- proteobacteria. The shared presence of these CSIs between Xanthomonadales and these other proteobacteria could result from a number of possibilities including later transfer of the corresponding genes between these groups of bacteria or independent occurrence of similar genetic changes in these groups. However, phylogenetic trees based upon these protein sequences showed that Xanthomonadales species and the Alpha- and/or Beta- proteobacteria containing similar CSIs branched separately from each other, indicating that the presence of similar CSIs in these groups of bacteria was not due to LGTs. Therefore, genetic changes leading to similar CSIs in these groups likely occurred independently due to similar functional requirements for these CSIs. Although in our work we have not come across many examples of LGTs between Xanthomonadales and other groups of bacteria, our analyses is based only on proteins that contain conserved indels. Such genes/proteins represent only a small fraction of the total genes that are found in various genomes. Because most of these proteins are involved in essential functions, they are less prone to LGTs. In contrast, extensive work that Menck and coworkers have carried out on identification of cases of LGTs is primarily on species from the genus *Xanthomonas* [16], [24]–[27], which have thus far not studied in detail.

Xanthomonadales is one of the deepest branching orders within the Class Gammaproteobacteria. Some of the other orders that branch in its proximity include *Chromatiales, Methylococcales*, *Cardiobacteriales*, *Legionelalles* and *Thiotrichales*. However, the relationship of Xanthomonadales to these other orders is presently not understood. In the present work, we also identified six other CSIs (Table 2, last six entries), which in addition to various Xanthomonadales were also uniquely shared by 1 or 2 species from these orders of Gammaproteobacteria. The shared presence of these CSIs by Xanthomonadales and some of these other orders of Gammaproteobacteria suggests that either these orders are closely related or that similar genetic changes have occurred in them independently. However, further information from additional species from these orders will be necessary to establish whether the Xanthomonadales and some of these other orders of Gammaproteobacteria are specifically related and form a higher taxonomic clade within the Gammaproteobacteria.

The focus of the present study was on identifying molecular signatures that are specific for either the entire Xanthomonadales order or some of its deep branching lineages. Thus far, we have not carried out careful analyses of various signature sequences that are specific for specific genera viz. *Xylella, Xanthomonas*, *Stenotrophomonas* and *Pseudoxanthomonas* and such studies will be part of our future work. Nonetheless, based upon the identified molecular signatures it is now possible to identify and circumscribe species from the order Xanthomonadales from all other bacteria in clear molecular terms based upon large numbers of discrete molecular characteristics. Based upon our earlier work on CSIs for other groups/phyla of bacteria, most of these CSIs have degree of predictive ability [21], [64]–[66] and thus they are useful in identifying both known as well as unknown species belonging to these clades (viz. Xanthomonadales) in different environments. Xanthomonadales harbor many important plant pathogens that cause a variety of diseases in economically important crops and plants [3]–[9]. In addition, they also contain Stenotrophomonas, which are opportunistic human pathogens [12]–[14]. Thus, novel methods for sensitive and specific identification of species from this order in different settings are of much importance. Most of the Xanthomonadales-specific CSIs discovered in this work are present in highly conserved regions of the genes/proteins. Hence, based upon these gene sequences degenerate PCR primers (based upon either flanking conserved regions or the indel region and a flanking conserved region) could be readily designed to examine the presence or absence of gene sequences containing these CSIs in any given sample [64], [67]. Thus, molecular probes based upon these CSIs and/or their flanking regions should provide novel and specific means for the detection of new as well as existing Xanthomonadales species in different environments. The Xanthomonadales-specific CSIs, in addition to their usefulness for evolutionary and diagnostic studies, also provide novel and useful tools for genetic and biochemical investigations and possible means for identification of agents that specifically target these plant pathogenic bacteria.

## Supporting Information

Figure S1 **A maximum-likelihood tree based upon concatenated sequences for 28 conserved proteins.** The tree shows the branching of Xanthomonadales group with Gammaproteobacteria. The tree was rooted using Alphaproteobacteria. The numbers on the nodes indicate statistical support for the nodes.
(PDF)

Figure S2 **Partial sequence alignment of a conserved region in the amino acid/peptide transporter showing a 7 aa insert that is specific for all Xanthomonadales.**
(PDF)

Figure S3 **Partial sequence alignment of large-conductance mechanosensitive channel protein showing the presence of a 5 aa insert that is commonly shared by Xanthomonadales.**
(PDF)

Figure S4 **Partial sequence alignment of lysyl-tRNA synthetase showing a 3 aa insert that is uniquely shared by all members of Xanthomonadales.**
(PDF)

Figure S5 **Partial sequence alignment of lipoyl synthase showing a 2 aa insert that is commonly shared by Xanthomonadales.**
(PDF)

Figure S6 **Partial sequence alignment of a conserved region in the queuine tRNA-ribosyltransferase showing a 1 aa insert that is specific for Xanthomonadales.**
(PDF)

Figure S7 **Partial sequence alignment of a conserved region in the acyl-(acyl-carrier-protein)–UDP-N-acetylglucosamine O-acyltransferase showing a 1 aa insert that is commonly shared by Xanthomonadales.**
(PDF)

Figure S8 **Partial sequence alignment of a conserved region in the TolQ protein showing a 1 aa insert that is commonly shared by Xanthomonadales.**
(PDF)

Figure S9 **Partial sequence alignment of a conserved region in alpha-2-macroglobulin domain-containing protein showing a 13 aa deletion that is uniquely present in Xanthomonadales.**
(PDF)

Figure S10 **Partial sequence alignment of a conserved region of DNA topoisomerase IV subunit B showing a 1 aa deletion that is commonly specifically found in Xanthomonadales.**
(PDF)

Figure S11 **Partial sequence alignment of DNA polymerase I showing a 1 aa deletion that is uniquely shared by all members of Xanthomonadales.**
(PDF)

Figure S12 **Partial sequence alignment of a conserved region of aromatic amino acid aminotransferase showing a 1 aa deletion that is uniquely shared by Xanthomonadales.**
(PDF)

Figure S13 **Partial sequence alignment of a conserved region of DNA polymerase III subunit beta showing a 1 aa deletion that is present in Xanthomonadales.** The CSI has also been found to be shared by *Marinomonas sp. MWYL1* and *Thioalkalivibrio sp. HL-EbGR7.*
(PDF)

Figure S14 **Partial sequence alignment of a conserved region of lipid-A-disaccharide synthase a 2 aa insert that is present in Xanthomonadales.** The CSI has also been found to be shared by *Cardiobacterium hominis, Allochromatium vinosum* and *Alteromonadales bacterium.*
(PDF)

Figure S15 **Partial sequence alignment of carbamoyl phosphate synthase large subunit a 1 aa insert that is present in Xanthomonadales.** The CSI has also been found to be shared by *Marinobacter* sp. ELB17.
(PDF)

Figure S16 **Partial sequence alignment of putative secreted protein showing a 1 aa insert that is present in Xanthomonadales.** The CSI has also been found to be shared by *Teredinibacter turnerae*.
(PDF)

Figure S17 **Partial sequence alignment of a conserved region of aminopeptidase P, showing a 1 aa deletion that is commonly shared by Xanthomonadales.** The CSI has also been found to be shared by *Thioalkalivibrio* sp. HL-EbGR7 and *Alkalilimnicola ehrlichii*.
(PDF)

Figure S18 **Partial sequence alignment of protoheme IX farnesyltransferase showing a 4 aa insert that is uniquely shared by subclade of Xanthomonadales after the divergence of** ***Rhodanobacter*** **sp. 2APBS1.**
(PDF)

Figure S19 **Partial sequence alignment of DNA polymerase III subunit alpha, showing a 1 aa insert that is uniquely present in Xanthomonadales except** ***Rhodanobacter*** **sp. 2APBS1.**
(PDF)

Figure S20 **Partial sequence alignment of a conserved region in the DEAD box helicase domain-containing protein showing a 1 aa insert that is specific for Xanthomonadales except** ***Rhodanobacter*** **sp. 2APBS1.**
(PDF)

Figure S21 **Partial sequence alignment of a conserved region in ribose-5-phosphate isomerase A, showing a 1 aa insert that is commonly shared by Xanthomonadales except** ***Rhodanobacter*** **sp. 2APBS1.**
(PDF)

Figure S22 **Partial sequence alignment of a conserved region in DNA polymerase I, showing a 1 aa insert that is uniquely shared by a subclade of Xanthomonadales except** ***Rhodanobacter*** **sp. 2APBS1.**
(PDF)

Figure S23 **Partial sequence alignment of aspartyl-tRNA synthetase, showing a 4 aa deletion that is commonly shared by all Xanthomonadales except** ***Rhodanobacter*** **sp. 2APBS1.**
(PDF)

Figure S24 **Partial sequence alignment of a conserved region of 2-oxoglutarate-dehydrogenase E1 component, showing a 1 aa deletion that is unique to Xanthomonadales except** ***Rhodanobacter*** **sp. 2APBS1.**
(PDF)

Figure S25 **Partial sequence alignment of a conserved region of coproporphyrinogen III oxidase, showing a 1 aa deletion that is unique to Xanthomonadales except** ***Rhodanobacter*** **sp. 2APBS1.**
(PDF)

Figure S26 **Partial sequence alignment of a conserved region in 2-oxoglutarate-dehydrogenase E1 component, showing a 1 aa deletion that is uniquely shared by Xanthomonadales except** ***Rhodanobacter*** **sp. 2APBS1.**
(PDF)

Figure S27 **Partial sequence alignment of a conserved region of asparagine synthase b protein that is showing a 4–5 aa insert, unique to Xanthomonadales except** ***Rhodanobacter*** **sp. 2APBS1.**
(PDF)

Figure S28 **Partial sequence alignment of a conserved region of Asparagine synthase b protein, showing a 1–2 aa insert that is uniquely shared by Xanthomonadales except** ***Rhodanobacter*** **sp. 2APBS1.** While genus Stenotrophomonas can be differentiated from other Xanthomonadales because of having 1 aa insert instead of 2 aa.
(PDF)

Figure S29 **Partial sequence alignment of a conserved region DNA polymerase III subunit alpha showing a 4 aa insert that is commonly shared by Xanthomonadales except** ***Rhodanobacter*** **sp. 2APBS1.** This CSI was previously identified by [28] as all Xanthomonadales specific signature.
(PDF)

Figure S30 **Partial sequence alignment of a conserved region of RecA showing a 2 aa insert that is commonly shared by Xanthomonadales except** ***Rhodanobacter*** **sp. 2APBS1.** This CSI was previously identified by [28] as all Xanthomonadales specific signature.
(PDF)

Figure S31 **Partial sequence alignment of a conserved region of MutS showing a 5 aa insert that is commonly shared by Xanthomonadales except** ***Pseudoxanthomonas suwonensis*** **and** ***Rhodanobacter*** **sp. 2APBS1.** This CSI was previously identified by [28] as all Xanthomonadales specific signature.
(PDF)

Figure S32 **Partial sequence alignment of a conserved region of DNA polymerase III subunit alpha, showing a 2 aa deletion that is commonly shared by all Xanthomonadales except** ***Pseudoxanthomonas suwonensis*** **and** ***Rhodanobacter*** **sp. 2APBS1.** These two have only 1 aa deletion at the same position.
(PDF)

Figure S33 **Partial sequence alignment of tRNA (guanine-N(1)-)-methyltransferase, showing a 2 aa insert that is commonly present in all members of Xanthomonadales except** ***Pseudoxanthomonas suwonensis*** **and** ***Rhodanobacter*** **sp. 2APBS1.**
(PDF)

Figure S34 **Partial sequence alignment of a conserved region in glucose-6-phosphate 1-dehydrogenase, showing a 4 aa deletion that is uniquely present in all Xanthomonadales except** ***Pseudoxanthomonas spadix*** **BD-a59 and** ***Rhodanobacter*** **sp. 2APBS1 which has 3 aa insert.**
(PDF)

Figure S35 **Partial sequence alignment of a conserved region of DNA ligase NAD dependent, showing a 57–65 aa insert that is commonly shared by all Xanthomonadales except** ***Pseudoxanthomonas suwonensis*** **and** ***Rhodanobacter*** **sp. 2APBS1.** Both these species do not contain the insert of same length. This CSI was previously identified by [28] as all Xanthomonadales specific signature.
(PDF)

Figure S36 **A Neighbor-joining tree based upon carbamoyl phosphate synthase large subunit sequence.** The Tree is showing the distinct branching of Xanthomonadales from various β-Proteobacteria with and without insert.
(PDF)

Figure S37 **Partial sequence alignment of a conserved region of Hypothetical protein XOO1065, showing a 1 aa deletion that is present in Xanthomonadales.** The deletion has also been found to be shared by few species from β-Proteobacteria but not in all of them.
(PDF)

Figure S38 **Partial sequence alignment of a conserved region of orotate phosphoribosyltransferase, showing a 1 aa deletion that is present in all Xanthomonadales.** The deletion has also been found to be shared by species from β-Proteobacteria.
(PDF)

Figure S39 **Partial sequence alignment of a conserved region of Putative ribonuclease HII, showing a 1 aa insert that is present in Xanthomonadales.** The CSI has also been found to be shared by few species from β-Proteobacteria but is not present in all.
(PDF)

Figure S40 **Partial sequence alignment of a conserved region of glycyl-tRNA synthetase subunit beta, showing a 2 aa insert that is present in Xanthomonadales.** The insert has also been found to be shared by some β-Proteobacteria.
(PDF)

Figure S41 **Partial sequence alignment of a conserved region of the septum-site determining protein MinD, showing a 1 aa deletion that is present in Xanthomonadales.** This CSI is also present in some species from β-Proteobacteria.
(PDF)

Figure S42 **A Neighbor-joining tree based upon sequences from hypothetical protein X001065.** The Tree is showing the Xanthomonadales and various β-Proteobacteria that share the 1 aa deletion. Species representing some other Gammaproteobacteria are also shown in tree.
(PDF)

Figure S43 **A Neighbor-joining tree for Proteobacterial species based upon orotate phosphoribosyltransferase sequences.** The Xanthomonadales and different β-Proteobacteria that contain the 1 aa deletion in this protein do not branch together in this tree suggesting that the deletion in these two groups have likely occurred independently. The tree shows only representative species from other Gammaproteobacteria and Alphaproteobacteria and it was rooted using sequences from Epsilonproteobacteria.
(PDF)

Figure S44 **A Neighbor-joining tree based upon sequences from putative ribonuclease HII.** The Tree is showing the Xanthomonadales and various β-Proteobacteria with insert. The tree also shows representative species from other Gammaproteobacteria and Alphaproteobacteria.
(PDF)

Figure S45 **A maximum-likelihood tree based upon sequences from glycyl-tRNA synthetase subunit beta.** The Tree shows the branching of Xanthomonadales separately from the other insert containing Betaproteobacteria. The species distribution of this insert could be explained by either the independent occurrence of a similar genetic event in the Betaproteobacteria and the Xanthomonadales, or that this insert was introduced in a common ancestor of the Beta- and Gamma-proteobacteria, followed by its loss from other Gammaproteobacteria after the divergence of deep-branching Xanthomonadales.
(PDF)

Figure S46 **A Neighbor-joining tree based upon sequences from septum site-determining protein MinD protein.** The Tree is showing the branching of Xanthomonadales distinctly from the other insert containing Betaproteobacteria.
(PDF)
