# Supplementary material for: Phylogenomics and Molecular Signatures for Species from the Plant Pathogen-Containing Order Xanthomonadales
Source: PLoS One. 2013 Feb 8;8(2):e55216. doi: 10.1371/journal.pone.0055216 (PMC3568101; doi:10.1371/journal.pone.0055216)
Supplement: Figure S1 — A maximum-likelihood tree based upon concatenated sequences for 28 conserved proteins. The tree shows the branching of Xanthomonadales group with Gammaproteobacteria. The tree was rooted using Alphaproteobacteria. The numbers on the nodes indicate statistical support for the nodes. (PDF) [file pone.0055216.s001.pdf]

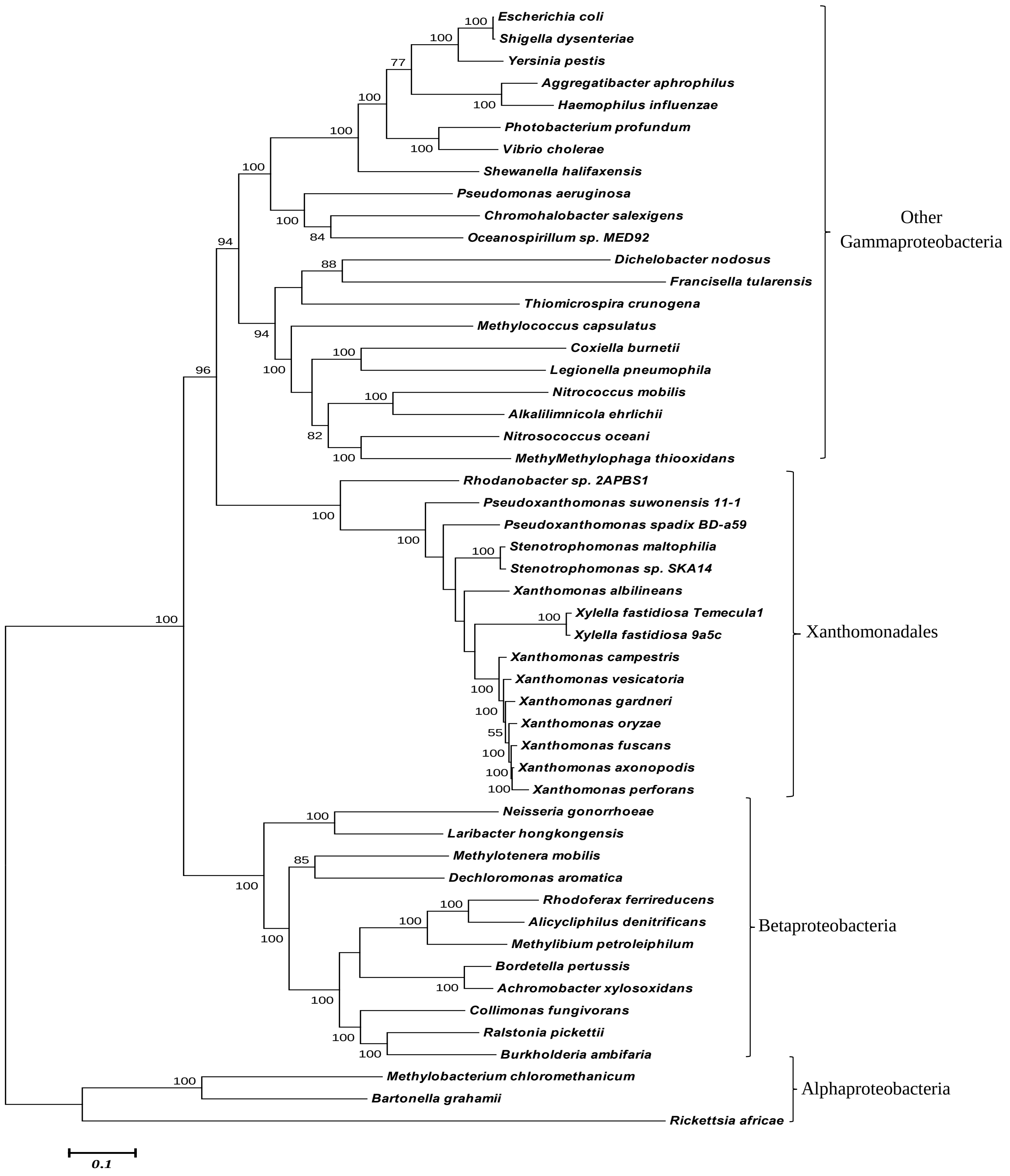

Figure S1

A maximum-likelihood (ML) tree based upon concatenated sequences for 28 conserved global proteins. The tree shows the branching of Xanthomonadales group with Gammaproteobacteria. The Tree the tree was rooted using Alphaproteobacteria. The numbers on the nodes indicate statistical support for the nodes.
