# Supplementary material for: Phylogenomics and Molecular Signatures for Species from the Plant Pathogen-Containing Order Xanthomonadales
Source: PLoS One. 2013 Feb 8;8(2):e55216. doi: 10.1371/journal.pone.0055216 (PMC3568101; doi:10.1371/journal.pone.0055216)
Supplement: Figure S2 — Partial sequence alignment of a conserved region in the amino acid/peptide transporter showing a 7 aa insert that is specific for all Xanthomonadales. (PDF) [file pone.0055216.s002.pdf]

			164		213
Xanthomonadales	Xylella fastidiosa Dixon	71275790	FTIFYMGINAGSLISPLITSWLAGQVFGT	PMQQNYK	VVFIASGIGMFLSL
	Xanthomonas fuscans	294667688	---------L-AM---VL-QL--EK----	EVMPS--	M--M---V--LI--
	Xanthomonas campestris	78046035	---------L-AM---VL-QL--EK----	EVMPS--	M--M---V--LI--
	Xanthomonas perforans	325927726	---------L-AM---VL-QL--EK----	EVMPS--	M--M---V--LI--
	Xanthomonas axonopodis	21241222	---------L-AM---VL-QL--EK----	DVMPS--	M--M---V--LI--
	Xanthomonas gardneri	325919443	---------L-AM---VL-QL--EK----	QAMPS--	M--M---V--LI--
	Xanthomonas oryzae	58583697	-----------GFVA-IL-GL--DKL---	EAMPA--	Y---------LI--
	Xanthomonas albilineans	285019501	-----------GFFA-IL-GL--DKL---	EAMPA--	Y---------LI--
	Xanthomonas vesicatoria	325915181	---------L-AM---VL-QL--EK----	QAMPA--	M--M---A--LI--
	Stenotrophomonas maltophilia	194367516	---------I-AM-A-VL-EY--RK----	SEMPS--	-------V--LI--
	Stenotrophomonas sp. SKA14	254520981	---------I-AM-A-VL-EY--RK----	SEMPS--	-------V--LI--
	Rhodanobacter sp. 2APBS1	352079924	--L--------A--A-IL--I--NK----	-GHH--H	---A---V--LI--
Other γ-Proteobacteria	Grimontia hollisae	262275906	---------L---LAGI-AGTVSMHYGWK		AG-LSA----L---
	Teredinibacter turnerae	254786138	Y--Y-----I--FFAT-LCG--GETFGWS		YG-G-A-V--L-G-
	Pseudoalteromonas tunicata	88857793	--L--------AFLA---CAY-GETYGWK		YG-GLA----LAG-
	Vibrionales bacterium	148980745	---------L-A-LGG--SGAA-ESYGWK		AG-L-A----VI--
	Aeromonas hydrophila	117618661	---------L---LAGV-CGAA-TAYGWQ		AA-VSA----L---
	Klebsiella pneumoniae	152971821	-SL------I--F-A---SG--IKSHGWH		WG-GIG----LVA-
	Vibrio fischeri	197334596	---------L---LAGVVSGSVT-VWGWK		AG-V-A----II--
	Acinetobacter baumannii	301346734	-S-------M--F-A----GL--KDHGWH		LG-GIG----LIA-
	Vibrio alginolyticus	91225921	---------L-A-LAGVVSGSVTTSYGWK		AG-V-A-V--LV--
	Colwellia psychrerythraea	71282472	---------L-AFLA-ITVGI-GE--NWH		YG--LA-C--LIG-
	Acinetobacter baumannii	193077887	-S-------M--F-A----GL--KDHGWH		LG-GIG----LIA-
	Aliivibrio salmonicida	209694404	---------L---LAGVVSGSVT-IWGWK		AG-A-A----II--
	Providencia stuartii	183598535	-SL------M--F-A---IGP-HEKYGWH		LG-GLG----LIA-
	Vibrio orientalis	261252243	---------L-A--AGVASGSVTNSFGWK		AG-LVA-L--II--
	Arsenophonus nasoniae	284007204	-SL------M--F-A---IGP-YEKYGWH		IG-GLG----LIA-
	Shewanella baltica	126173789	-----A---L-ATLA---CAY-GETYGWK		YG-GLA-L--LAG-
	Kangiella koreensis	256821955	--L------L--FL-SITCGIIGIVWGWA		YG-GLA----L-G-
	Alteromonas sp. SN2	333894220	YALY-----I----AY-V-GY-QENWGWD		YA-G-AA---AIG-
	Idiomarina baltica	85711680	Y-LY-----I--I-GYFV-GY-QENAGYH		WG-G-AAV--AFG-
	Glaciecola sp. HTCC2999	221133332	Y-LY-----I-AV-GYTVCGY-QVNMGYH		WA-G-AAV--AIG-
	Salmonella enterica	224582899	-SLL-AAG-I--IAA-IACGLA-QWYGWH		-G-ALA-V---IG-
	Escherichia coli	300947710	-SLL-AAG-I--IAA-IACGLA-QWYGWH		-G-ALA-G---IG-
α-Proteobacteria	Novosphingobium nitrogenifigen	326388414	-SL--V---I-ATAA--LCA---DRWGWA		PA-E-TSV--V-G-
	Nitrobacter hamburgensis	92119225	YS---V---L-AMLA--VAGT-GEEIGWP		YG-G-A----MIG-
	Sphingomonas sp. S17	332185434	-S---------GALG-IVCGV-GET-GWA		YG-G-A-V--L-G-
	Hirschia baltica	254293389	---------L--F-ASFACGI-GIVYGWK		YG-GLA----L-G-
	Hyphomonas neptunium	114800217	--L------L--FLATWTCGI-GIVYGWA		WG-G-A-V--L-G-
	Erythrobacter sp. SD-21	149185140	Y--------V-AATASI-CGY-GQTYGWQ		YG-GLA-F--L-G-
	Phenylobacterium zucineum	197105272	-Q---L-----VIAA---AGT-GEG-GWH		YG-G-A-V--LIA-
	Sphingopyxis alaskensis	103486444	Y--------L-AFLGS-LCGY-GETYGWA		YG-G-A-F--L-G-
	Erythrobacter litoralis	85375324	Y--------L-AA-GS-LCGYIGETYGWA		YG-GLA----L-G-
	Zymomonas mobilis	56551897	---------L-GALG---CGYVGEKYGWR		YG-GMA-V--AIG-
	Parvularcula bermudensis	304321342	--L--V---L-AFLGAIWAGY-GEVHGWK		YG-GLA-V--L-G-
	Citromicrobium bathyomarinum	296282079	Y----I---L-AAAGTI-AGY-GETIGWA		YG-G-A----L-G-
β-Proteobacteria	Chromobacterium violaceum	34496617	---------T----A--VCGT-GDTGNPA	DF-	WG-M-A-F--L---
	Laribacter hongkongensis	226941725	Y--------L--FLA---CGY-GDTGNPA	DFR	WG-LTA-V--L--V
	Methylophilales bacterium	118594916	YSF--V---M-AF-A--LIGYVGEVIDWH		YG-VLAAV---MG-

Figure S2

Partial sequence alignment of a conserved region in the amino acid/peptide transporter showing a 7 aa insert that is specific for all Xanthomonadales.
