# Supplementary material for: Phylogenomics and Molecular Signatures for Species from the Plant Pathogen-Containing Order Xanthomonadales
Source: PLoS One. 2013 Feb 8;8(2):e55216. doi: 10.1371/journal.pone.0055216 (PMC3568101; doi:10.1371/journal.pone.0055216)
Supplement: Figure S3 — Partial sequence alignment of large-conductance mechanosensitive channel protein showing the presence of a 5 aa insert that is commonly shared by Xanthomonadales. (PDF) [file pone.0055216.s003.pdf]

Group	Species	ID	40	Insert	85
Xanthomonadales	Xanthomonas fuscans	294667079	IIMPPIGWAIGNVDFSRLAWVLK	PAGVD	ATGKEIPAVAIGYGDFIN
	Xanthomonas axonopodis	21244033	-----------------------	-----	------------------
	Xanthomonas vesicatoria	325916668	-----------------------	-----	------------------
	Xanthomonas gardneri	325919139	-----------------------	-----	------------------
	Xanthomonas oryzae	58583004	-----------------------	-----	----D-------------
	Xanthomonas perforans	325928477	-----------------------	-----	------------------
	Xanthomonas campestris	289665119	-----------------------	--S--	------------------
	Xanthomonas albilineans	285019030	-------LLV-GM---HW-LT--	A-T--	-A--PVQ--V--I---L-
	Stenotrophomonas sp. SKA14	254525001	-----L-LL--K----Q---T-S	--SIG	-D-------V--------
	Stenotrophomonas maltophilia	194366968	-----L-ML--K----Q---T-S	--SIG	-D-------V--------
	Xylella fastidiosa	15836644	--S-L--VMV-GI---K-SLT--	A-T--	-A---V---V------L-
	Rhodanobacter sp. 2APBS1	352090462	--------LT-GI---AMK----	-GDDS	DPKHK-AE---Q--A---
	Pseudoxanthomonas suwonensis	319786092	-----L--L-------D---T-S	--R-A	-D-S-----V----E-L-
	Pseudoxanthomonas spadix	357416396	L------MLV---------IT--	E-S--	-A---V---VVA--S---
Other Bacteria	Actinobacillus minor	257465685	VV------L--G---KD--IEIA		PAKEGAE--MLK--A--Q
	Aeromonas hydrophila	117621104	V------LIL-G----D--VT--		-AEGST---V-A--K--Q
	Aeromonas salmonicida	145298068	V------LIL-G----D--VT--		-AEGAT---V-A--K--Q
	Azotobacter vinelandii	226946144	V----L-LL--G----D--IT--		QAQGDM---VLA--R--Q
	Candidatus Hamiltonella	238897728	-------LL--G--LKQFSF--R		EARNDV---IMN--S-L
	Citrobacter koseri	157148865	-----L-LL--GI--KQF-FT-R		EAQGD----VMH--V--Q
	Cronobacter turicensis	260599610	-----L-LL--GI--KQF-LT-R		PAVGDT---IMH--V--Q
	Dickeya dadantii	307132810	-----L-LL--G---KQFH---R		EAQGN-A--S-N--V--Q
	Dickeya zeae	251788000	-----L-LL--G---KQFH---R		EAQGNLA--S-N--T--Q
	Edwardsiella tarda	294638025	V---VL-LL--G---KQFH---R		EAANGA---VVN--Q--Q
	Enterobacter cloacae	296104998	-----L-LL--GI--KQF-FT-R		EAQGD----VMH--V--Q
	Erwinia tasmaniensis	188535247	-----L-LL--G---KQFS-I--		PADGAA---VME--V-LQ
	Escherichia albertii	170769538	-----L-LL--GI--KQF-VT-R		DAQGDV---VMH--V--Q
	Escherichia coli	15803818	-----L-LL--GI--KQF-VT-R		DAQGD----VMH--V--Q
	Haemophilus ducreyi	33152991	V-------L--G---KD--IQIA		PAKEGAE--MLK--A--Q
	Mannheimia haemolytica	261493883	VV------L--G---KD--IEIA		PAKEGAE--MLK--A--Q
	Pantoea ananatis	291619146	-----L-LL--G---KSFS----		PAVGNA---VME--V-LQ
	Pasteurella multocida	15603429	VV--VL-ILT-G---KD-SI---		EAAG-V---TLN--A--Q
	Pectobacterium atrosepticum	50122923	-----L-LL--G---KQ-SLI-R		DAQG-----VMN--A--Q
	Proteus mirabilis	197287097	V----L-LL--GI--KQFSL--R		EAHGD----ILN--A--Q
	Providencia alcalifaciens	212709008	V----L-LI--G---KQFSL--R		EAQGD----VMN--M--Q
	Pseudomonas syringae	28871773	V----L-LL--G----D--VT-R		PAQGTA---LLA--K--Q
	Salmonella enterica	8650506	-----L-LL--GI--KQF-VT-R		DAQGD----VMH--V--Q
	Serratia odorifera	270264344	-----L-LL--G---KQFHL--R		EAQGAV---VMN--S--Q
	Shewanella sp. W3-18-1	120597433	V------IIL-G-N--D-SI--Q		-AQGDA-S-V-A--K--Q
	Shigella sonnei	74313809	-----L-LL--GI--KQF-VT-R		DAQGD----VMH--V--Q
	Sodalis glossinidius	85060228	V---QL-LL--GI--KQFS----		PAQGDT---VMK--I-LQ
	Vibrio cholerae	153830945	-------IIL-G-N--D-SF--L		-AQGDA---V-A--K--Q
	Yersinia pestis	22127889	-----L-LLL-G---KQFHF--R		-AEGT----VMN--T--Q

Figure S3

Partial sequence alignment of Large-conductance mechanosensitive channel protein showing the presence of a 5 aa insert that is commonly shared by Xanthomonadales.
