# Supplementary material for: Phylogenomics and Molecular Signatures for Species from the Plant Pathogen-Containing Order Xanthomonadales
Source: PLoS One. 2013 Feb 8;8(2):e55216. doi: 10.1371/journal.pone.0055216 (PMC3568101; doi:10.1371/journal.pone.0055216)
Supplement: Figure S4 — Partial sequence alignment of lysyl-tRNA synthetase showing a 3 aa insert that is uniquely shared by all members of Xanthomonadales. (PDF) [file pone.0055216.s004.pdf]

			34		85
Xanthomonadales	Stenotrophomonas maltophilia	194365604	PNDFRREDFAGRLQEEFADA	EQW	TAEALEGNGRQVKMAGRLMAKRIMGKASF
	Stenotrophomonas sp. SKA14	254524293	-----------Q--------	G--	-----------------------------
	Xylella fastidiosa	15837714	---VK--HY-ADV-VA--NV	-T-	------AS-DR-R---------L------
	Xanthomonas oryzae	58582495	----V--H---D--A-----	DT-	-PD---AS--T----------V------
	Xanthomonas axonopodis	21242620	----V--H---D--A-----	DT-	-P----AS--T-R---------V------
	Xanthomonas fuscans	294624918	----V--H---D--A-----	DT-	-P----AS--T-R---------V------
	Xanthomonas campestris	188991535	----V-TH---D--A---E-	-T-	------AAD-S--L--------V------
	Xanthomonas albilineans	285018128	------S----DV-A----L	-R-	--D---AQ--SLRL----L---V------
	Xanthomonas perforans	325925183	----V--H---D--A-----	DT-	-P----AS--T-R---------V------
	Xanthomonas vesicatoria	325916712	----V--H---D--A-----	DT-	-P----AS--T-----------V------
	Xanthomonas gardneri	325922146	----V--Q---D--A-----	-T-	-P----AS--T-R---------V------
	Pseudoxanthomonas suwonensis	319787301	----K-A-Y--D--A-----	G--	-G----ALP-T-AI--------V------
	Pseudoxanthomonas spadix	357417570	------HAL--D--A-----	DTF	--GV---LD---RL--------V------
	Rhodanobacter sp. 2APBS1	352081997	----KVDS---D--T----K	DVH	----V-AAV-R------IVL--VQ--V--
Other γ-Proteobacteria	Actinobacillus minor	240949401	--K---DAY-KD-HAKYD-V		DG---KEQDI---V---I-L--V------
	Aeromonas hydrophila	117617708	------DSLS-D-HA--G-K		S-DE-AAL-KR--I---I-TR--------
	Arsenophonus nasoniae	284008433	-------YLSSD-HGKY--Q		E--Q-IDLDID-SV---MITR--------
	Candidatus Regiella	304414310	------DTLSHE-H-QYDNK		DNDD-SKLDIK--V---M-TR--------
	Citrobacter freundii	30961858	------DHTSDQ-HAD-DAK		EN-E--ALNIE-SV---M-TR--------
	Cronobacter sakazakii	156932659	------DHTSDQ-HAD-DGK		EN-E--ALNIE-CV---M-TR--------
	Dickeya dadantii	307129434	------DSISD--HASYDEK		DN-E--AL-LE-NV---M-TR--------
	Edwardsiella ictaluri	238921195	------DTTSDK-HSLYDGK		SK-E--ALNVE-SV---M-TR--------
	Enterobacter cancerogenus	261342292	------DHTSDQ-HAD-DAK		EN-E--ALNVE-AV---M-TR--------
	Erwinia amylovora	292487147	------DRTSDQ-HAA-DGK		EN-E-VEL-IE-SV---M-TR--------
	Escherichia albertii	170766125	------DHTSDQ-HA--DGK		EN-E--ALNIE-AV---M-TR--------
	Escherichia coli	146689	------DHTSDQ-H---DAK		DNQE--SLNIE-SV---M-TR--------
	Haemophilus ducreyi	33152397	--R---DAY-YA--A-Y-EM		AGD--KDADV---VV--I-L--V------
	Halorhodospira halophila	121996858	------DAL-AD-H-RYGPL		DS----QE-AR-TVG--M----V------
	Idiomarina baltica	85712972	------DSL-KD-HA-YDE-		DK-S--QQ-VR--V---M-TR--------
	Mannheimia haemolytica	254360791	--T---DAL-KD-HAQYDAV		EG-T-KEQNI---V---I-L--V------
	Nitrosococcus halophilus	292492473	--N---NVM--E--A-YHHR		S--E--KRPLR--V---M-TR--------
	Pantoea ananatis	291618680	------DTTSD--HASYDEK		SN-E--DL-IE-SV---M-TR--------
	Pectobacterium atrosepticum	50119719	------DSISDH-HA--DAK		EN-E--EL-IE-TV---M-TR--------
	Photorhabdus asymbiotica	253988707	----H--NMSEE-HAKYD-K		-Q-E--ALNIE-TVG--M-TR--------
	Proteus mirabilis	197285859	------DAMSDE-HQKYDAM		S--E-DAANVE-AI---M-TR--------
	Providencia alcalifaciens	212710039	-------NVSDK-HA--GEK		---E--DLNVE-TI---M-TR--------
	Psychromonas sp. CNPT3	90409171	------KNISDE-H-KYD-K		-K-E-DELAVD-SI---M-TR--------
	Salmonella enterica	161506412	------DRTSDQ-HA--DAK		E--E--ALNIE-SV---M-TR--------
	Serratia proteamaculans	157372123	------DTTSDK-HAAYG-K		DN-E--AL-VE-TV---M-TR--------
	Shigella boydii	82545487	------DHTSDQ-HA--DGK		EN-E--ALNIE-AV---M-TR--------
	Sodalis glossinidius	85059967	------DAVSDQ-HARYGEK		SN-E--ALNIE-SI---M-TR--------
	Tolumonas auensis	237807750	------DQISNE-HAA-D-K		SQDE-AAEKHY--I---I-TR--------
	Xenorhabdus bovienii	290476398	-------NISED-HAKYD-K		-Q-E--ALNIE-TV---M-TR--------
	Yersinia pestis	167470510	-------NLSDQ-HA--DSK		EN-E--ALNID-TV---M-TR--------
β-Proteobacteria	Thiobacillus denitrificans	74316911	----E-R-Y----AH-HG-K		SK----AEAVD-QL---M-L--V------
	Burkholderia ubonensis	167586750	----QPTHH-AD--AK---S		DKA---A-PVE-AV---M-L--V------
	Pusillimonas sp. T7-7	332284054	----QPDNV-AA-HAAYDEL		DKD--AEV-K---V---M-L--V------
	Nitrosospira multiformis	82703393	----P--H-SAD-HNTYSEH		ST-T--AEPVP-RV---MVL--V------
	Cupriavidus metallidurans	94309979	----QPTHQ--A-HAQYGET		DQAT--ASPVE-SI---M-L--V------

Figure S4

Partial sequence alignment of Lysyl-tRNA synthetase showing a 3 aa insert that is uniquely shared by all members of Xanthomonadales.
