# Supplementary material for: Phylogenomics and Molecular Signatures for Species from the Plant Pathogen-Containing Order Xanthomonadales
Source: PLoS One. 2013 Feb 8;8(2):e55216. doi: 10.1371/journal.pone.0055216 (PMC3568101; doi:10.1371/journal.pone.0055216)
Supplement: Figure S5 — Partial sequence alignment of lipoyl synthase showing a 2 aa insert that is commonly shared by Xanthomonadales. (PDF) [file pone.0055216.s005.pdf]

| Group | Species | GI | 156 | | 209 |
|---|---|---|---|---|---|
| Xanthomonadales | Xanthomonas oryzae | 58583575 | DGGAQHFVDCISAIRTSSPNTRIEILTPDFR | GK | GRMDRALDILALSPPDVFNHN |
| | Xanthomonas axonopodis | 21241439 | ------------------------------- | -- | -------E------------- |
| | Xanthomonas campestris | 78046285 | ---------------A--------------- | -- | -------E------------- |
| | Xanthomonas albilineans | 285017612 | -------A---A---VAA-A----------- | -- | ---E---E---TH-------- |
| | Xanthomonas vesicatoria | 325915038 | -----------A---A--------------- | -- | -------E------------- |
| | Xanthomonas gardneri | 325920078 | ---------------V---K----------- | -- | -------E------------- |
| | Pseudoxanthomonas spadix | 357418483 | -------A-------SH--G--V-------- | -- | -------E---T--------- |
| | Pseudoxanthomonas suwonensis | 319785906 | -----------A---ERA-G----------- | -- | ---E---G---QN-------- |
| | Rhodanobacter sp. 2APBS1 | 352079802 | ----E--AA--R-T-HA---I---------- | -- | ---E---EV-KEF-------- |
| | Xylella fastidiosa | 71274929 | -----------A---Q-A-Q----------- | -- | -----------AC-------- |
| | Stenotrophomonas sp. SKA14 | 254521222 | ---------------EK--G----V------ | -- | ---E---E---QN-------- |
| | Stenotrophomonas maltophilia | 194367214 | ---------------EK--G----V------ | -- | ---E---E---QN-------- |
| Other γ-Proteobacteria | Acinetobacter lwoffii | 262376030 | -----------KEA-AL----LL---V---- | | ----I--R-MTEC-------- |
| | Acinetobacter radioresistens | 255320490 | -----------QE--KCC--------V---- | | --L-L--S--SQ--------- |
| | Acinetobacter sp. ADP1 | 50084218 | -----------K---ATN-H-L----V---- | | --LEI--K---S--------- |
| | Aeromonas hydrophila | 117620976 | -------A---KQ--EH--Q----------- | | ---EQ--EVFRET-------- |
| | Aeromonas salmonicida | 145298084 | -------A---KQ--EH--Q----------- | | ---EQ--EVFRET-------- |
| | Aliivibrio salmonicida | 209694542 | -------A--NRE--ELN-EI---T-V---- | | --------AMHSN-------- |
| | Alteromonas macleodii | 239996597 | -----------N---EH--T-T--V-V---- | | -------E-FKNGV------- |
| | Arsenophonus nasoniae | 284009244 | -------A---T---KKN--IK--T-V---- | | -------T---TA-------- |
| | Candidatus Regiella | 304413537 | -------A---A---AEN-GIK--T-V---- | | ----------VNP-------- |
| | Citrobacter koseri | 157146756 | -------A---T---EK--AIK--T-V---- | | -------E--NAT-------- |
| | Colwellia psychrerythraea | 71282264 | -----Q-A--VKE-GEQA---KV---V---- | | -------E--NQN--H----- |
| | Dickeya dadantii | 307130038 | -------A-------RK---I---T-V---- | | ----------TVT-------- |
| | Edwardsiella ictaluri | 238920822 | -------A---H---AK--QI---T-V---- | | -------E--HDN-------- |
| | Erwinia amylovora | 292487598 | -------A-------EKN-SIK--T-V---- | | -------E--TAT-------- |
| | Escherichia coli | 146617 | -------A---T---EK--QIK--T-V---- | | ----------TAT-------- |
| | Klebsiella pneumoniae | 152969231 | -------A-------EKN-SIK--T-V---- | | ----------TVT-------- |
| | Marinomonas sp. MED121 | 87119255 | -------I---NET-KE---IE--T-V---- | | ----K-----E-A-------- |
| | Methylophaga thiooxidans | 254491589 | ----R--IR--DE--QQ--E-N----V---- | | ----V--E--TQ----I---- |
| | Moritella sp. PE36 | 149909673 | -----------RE--LLN-EIK----V---K | | ----K--E-FKDT-------- |
| | Nitrosococcus halophilus | 292490711 | ----A--AR--Q-L--Q--Q-C--V-V---- | | -------EA--VA---I---- |
| | Pantoea ananatis | 291616671 | -------A---T---EK--SIK--T-V---- | | -------E--TAT-------- |
| | Pectobacterium wasabiae | 261822399 | -------A-------RKN-QI---T-V---- | | -------E--TAT-------- |
| | Photobacterium profundum | 54310021 | -----------RE--EK--EIH--T-V---- | | ----------QGT--N----- |
| | Photorhabdus luminescens | 37525258 | -------A-------EKN-SIK--T-V---- | | -------E--TAT-------- |
| | Proteus mirabilis | 197284320 | -------A---T---EKN--I---T-V---- | | ----K--E--TDT-------- |
| | Providencia stuartii | 183598101 | -------A-------EK--SIK--T-V---- | | -------E--TET-------- |
| | Pseudoalteromonas atlantica | 109897874 | -----------N---EH--T-K--V-V---- | | ----K--E--KNGV------- |
| | Pseudomonas fluorescens | 77461184 | -------A---RE--KL---VQL-T-V--Y- | | ----I--E-T-AE-------- |
| | Salmonella enterica | 161504193 | -------A---T---AK--EIK--T-V---- | | -------E--NAT-------- |
| | Serratia proteamaculans | 157369438 | -------A---A---AKN-TIK--T-V---- | | -------E--TET-------- |
| | Shewanella baltica | 153001833 | -------A---RE--KLN-DIK--T-V---- | | --I-A-----STE-------- |
| | Shigella boydii | 187731162 | -------A---T---EK--QIK--T-V---- | | ----------TAT-------- |
| | Sodalis glossinidius | 85058770 | -------A-------AKN--I---T-V---- | | -------E-INAA-------- |
| | Teredinibacter turnerae | 254784821 | -------A---RES-AL--ELQV-------- | | ----I------QE-------- |
| | Tolumonas auensis | 237809575 | -------A---RE--LA-------T------ | | ----K---VFRET-------- |
| | Vibrio orientalis | 261253679 | -------A--NRE--ALN--I---T-V---- | | ----V---LMKDN-------- |
| | Yersinia pestis | 22125074 | -------A-------AKN-TIK--T-V---- | | ----------TAT-------- |
| α-Proteobacteria | Rickettsia conorii | 15893068 | ----S--AE---E--K-----T--------L | | -KEG-AE-I-NAK------- |
| | Stappia aggregata | 118589899 | -------A-V-A---KTA-S-T--V-----L | | -K-G--E-VVAAK------- |
| | Bartonella rochalimae | 319404091 | -------AKV-Y---QKA-T-T--V------ | | NK-G--E-VVAAK------- |
| | Mesorhizobium sp. BNC1 | 110633976 | -------A-V-Q---AAT-Q-T--------L | | -K-G--ELVVAAK------- |
| | Bartonella sp. 1-1C | 319407103 | -------AKV-Y---QKA-T-T--V------ | | NK-G--E-VVAAK------- |
| | Ehrlichia ruminantium | 258551496 | ----G---E--EE--KRDS-VT--------L | | NKHD-I-KI-KAF---Y--- |
| | Rhizobium etli | 190891635 | ----E--EKV-W---AA--A-T--------L | | KKPG--ERVVAAK------- |
| β-Proteobacteria | Simonsiella muelleri | 294789909 | -------A---N---ET----K----V---- | | --L-I--E---KN----M--- |
| | Kingella oralis | 238022577 | -------A---NE--KT----K----V---- | | --L-I------KN----M--- |
| | Rubrivivax benzoatilyticus | 332527501 | -----------T---EA--A----V-V---- | | --L-K--A--KTA----M--- |
| | Neisseria meningitidis | 319410466 | -------A---K---ET----K----V---- | | --L-I--K---ET----M--- |
| | Cupriavidus metallidurans | 94309006 | ------Y-----QT-EL-------V-V---- | | --L-------QAC----M--- |
| | Burkholderia dolosa | 254251309 | ----G---E--REV-AQ--E----------- | | --L----S--NAA----M--- |
| | Lutiella nitroferrum | 224825269 | -------A----E--KL--S-Q--V-V---- | | --L-I-----TQT----M--- |

**Figure S5**

Partial sequence alignment of Lipoyl synthase showing a 2 aa insert that is commonly shared by all members of Xanthomonadales.
