# Supplementary material for: Phylogenomics and Molecular Signatures for Species from the Plant Pathogen-Containing Order Xanthomonadales
Source: PLoS One. 2013 Feb 8;8(2):e55216. doi: 10.1371/journal.pone.0055216 (PMC3568101; doi:10.1371/journal.pone.0055216)
Supplement: Figure S6 — Partial sequence alignment of a conserved region in the queuine tRNA-ribosyltransferase showing a 1 aa insert that is specific for Xanthomonadales. (PDF) [file pone.0055216.s006.pdf]

| | | | 289 | | 339 |
|---|---|---|---|---|---|
| Xanthomonadales | Stenotrophomonas maltophilia | 194365393 | IRNSQYARDMDPIEPGCGCVACT | G | GYTRSYLRHLDRCNEMLAPMLGTLHNL |
| | Stenotrophomonas sp. SKA14 | 254524329 | ----------------------- | - | --------------------------- |
| | Xanthomonas albilineans | 285018698 | ---AK-E--L---------Y--S | S | ----A---------------------- |
| | Xanthomonas oryzae | 58582107 | ---AK-E--L-T-------H--S | S | --------------------------- |
| | Xanthomonas fuscans | 294624101 | ---AK-E--L-T-------H--S | S | ----A---------------------- |
| | Xanthomonas axonopodis | 21243246 | ---AK-E--L-T-------H--S | S | ----A---------------------- |
| | Xanthomonas perforans | 325927083 | ---AK-E--L-T-------H--S | S | ----A---------------------- |
| | Xanthomonas campestris | 21231815 | ---AK-E--L-T-------H--S | S | ----A---------------------- |
| | Xanthomonas gardneri | 325919631 | ---AK-E--L-T-------H--S | S | --------------------------- |
| | Xanthomonas vesicatoria | 325915106 | ---AK-E--L-T-------H--S | S | --------------------------- |
| | Pseudoxanthomonas spadix | 357416954 | ---AR-EK-L---------H--R | N | -F------------------------- |
| | Xylella fastidiosa | 28198110 | ---AC----V----Q----P--V | - | ----A------------S---S---- |
| | Rhodanobacter sp. 2APBS1 | 352086362 | ---AK--S-TAV--E--D-Y--S | H | -FS-A---------I-GSQ-A-M--- |
| Other γ-Proteobacteria | Acinetobacter lwoffii | 262377442 | ----K-RH-QS-LD-H-D-YT-Q | | NF--A--F--EK-G---GS----I--- |
| | Aeromonas salmonicida | 145299555 | ---AK-RS-TSTLDAD-D-YT-K | | N------Y---K---I-GAR-N-I--- |
| | Aggregatibacter aphrophilus | 251793023 | ---AK-KD-TS-LD-H-D-YT-K | | H--KA--Y---K-G-I-GAR-N-I--- |
| | Alcanivorax borkumensis | 110833362 | ---ARHRH-LA-L-AE-D-YT-Q | | NFS----H---------GSQ-N-I--- |
| | Alteromonas macleodii | 196156164 | ---AKHRN-TS-LDEN-D-YT-K | | N-S----H---K---I-GAR-N-I--- |
| | Arsenophonus nasoniae | 284006392 | ---AK-KS-TS-LDAH-D-YT-S | | N-S-A--H-------I-GAR-N----- |
| | Buchnera aphidicola | 219681508 | ---KK-KK-LSCLDKT-V-YT-R | | Y-S----H---A---I-GAR-N-I--- |
| | Candidatus Regiella | 304413741 | ---AKHKE-DSTLDKN-D-YT-C | | H-S-A--H---------GAR-N-I--- |
| | Cardiobacterium hominis | 258544914 | L--TIHK--LR---E--D-YT-R | | H-S-A--H---K-----GAR-N-I--- |
| | Citrobacter rodentium | 283784224 | ---AK-KS-TS-LDAE-D-YT-R | | N-S-A--H-------I-GAR-N-I--- |
| | Colwellia psychrerythraea | 71278113 | ---AAHKT-VA-LDDT-D-YT-K | | N-S-A--H---K-K-I-GSQ-N----- |
| | Cronobacter sakazakii | 156935045 | ---AK-KS-TG-LD-E-D-YT-R | | N-S-A--H-------I-GAR-N-I--- |
| | Dichelobacter nodosus | 146328961 | L---R-KE-LR--DET-N-YT-Q | | R-S----H---K---I-GAR-N-I--- |
| | Edwardsiella ictaluri | 238918972 | ---A--KE-TTALD-E-D-YT-R | | N-S-A--H-------I-GAR-N-I--- |
| | Enhydrobacter aerosaccus | 257455335 | -K-AVHRF-QS-LDSA-D-YT-Q | | NFS-A--H--FK-----SAQ---I--- |
| | Enterobacter cancerogenus | 261341000 | ---AKHKS-TS-LDSE-D-YT-R | | N-S-A--H-------I-GAR-N-I--- |
| | Erwinia tasmaniensis | 188534664 | ---AK-KD-IS-LDAE-D-YT-R | | N-S-A--Y-------I-GAR-N-I--- |
| | Escherichia coli | 147966 | ---AK-KS-TG-LD-E-D-YT-R | | N-S-A--H-------I-GAR-N-I--- |
| | Francisella philomiragia | 167627730 | ---AKHKD-VSLLD-N-D-YT-Q | | NFS----H---KTQ-I-GSR-N-I--- |
| | Francisella tularensis | 254369198 | ----K-KD-TS-LD-N-D-YT-K | | NF-K---H---KTK-I-GSR-N-I--- |
| | Grimontia hollisae | 262274807 | ---AK-KN-TTALD-E-D-YT-R | | N-SKA--H-------I-GAR-N-I--- |
| | Haemophilus ducreyi | 33152439 | ---AK-KN-TT-LDAE-D-YT-K | | N--KA--Y---K-G-I-GAR-N-I--- |
| | Haemophilus influenzae | 53732897 | ---AK-RD-TN-LD-E-D-YT-K | | N--KA--Y---K-G-I-GAR-N-I--- |
| | Kangiella koreensis | 256822292 | L---HNKT-TG-LDEA-D-YT-- | | N-S-A--H---K---I-GAK-N-I--- |
| | Mannheimia haemolytica | 261493102 | ---AK-RN-IT-LD-E-D-YT-K | | N--KA--Y---K-G-I-GAR-N-I--- |
| | Methylococcus capsulatus | 53805144 | ---AR-RD-LR--DEE---YT-R | | H-S-A--------G-I-GAR-N-I--- |
| | Methylophaga thiooxidans | 254490694 | ----RFKH-TG-LD-Y-D-YT-Q | | N-S-A--H----TG---G-R-N-I--- |
| | Oceanobacter sp. RED65 | 94501956 | L--AANKT-HG--D-E-D-YT-Q | | NFS-A--H---K-K-IQGCT-N-I--- |
| | Pantoea ananatis | 291616516 | ---AKHKD-VS-LDAE-D-YT-R | | N-S-A--H-------I-GAR-N-I--- |
| | Pasteurella multocida | 15602094 | ---AK-RE-TS-LD-E-D-YT-K | | H--KA--Y---K-G-I-GAR-N-I--- |
| | Pectobacterium carotovorum | 253687414 | ---AK-KD-VS-LDEH-D-YT-R | | N-S-A--H-------I-GAR-N-I--- |
| | Photobacterium damselae | 269103294 | ---ASHKT-TT-LD-Q-D-YT-R | | N-SKA--H---K---I-GAR-N-I--- |
| | Providencia stuartii | 183601033 | ---AK-KS-TSTLD-E-D-YT-R | | H-S-A--H-------I-GAR-N-I--- |
| | Pseudomonas entomophila | 104780220 | ---AFHRH-DS-LD-T-D-YT-- | | NFS-A--H---K-G---SS--N-I--- |
| | Saccharophagus degradans | 90021053 | ---AKHRH-TG-LD-T-D-YT-Q | | NFS-A--H---K-G-I-GAQ-N-I--- |
| | Salmonella enterica | 62179017 | ---AKHKS-TS-LDAE-D-YT-R | | N-S-A--H-------I-GAR-N-I--- |
| | Serratia odorifera | 270263541 | ---AKHKD-TS-LDKD-D-YT-R | | N-S-A--H-------I-GAR-N-I--- |
| | Shewanella sediminis | 157376027 | ---ARHRD-TS-LDDK-D-YT-K | | N-S-A--Y-------I-GAR-N-I--- |
| | Shigella flexneri | 24111784 | ---AK-KS-TG-LD-E-D-YT-R | | N-S-A--H-------I-GAR-N-I--- |
| | Sodalis glossinidius | 85058624 | ---AR-KD-VA-LDAE-D-YT-R | | N-S-A--H-------I-GAR-N-I--- |
| | Tolumonas auensis | 237808775 | ---AK-KD-VTSLDAE-D-YT-K | | N--K---H---------GAR-N-I--- |
| | Vibrio cholerae | 15640760 | ---AAHKT-TT-LD-H-D-YT-K | | N-SK---H-------I-GAR-N-I--- |
| | Xenorhabdus nematophila | 300721774 | ---AKHKE-TSSLDEH-D-YT-R | | N-S-A--H-------I-GAR-N-I--- |
| β-Proteobacteria | Marinomonas posidonica | 333907799 | ---A--RH-I--LDKE-D-YT-K | | NFS-A--H---K-Q--VGAQ-N-I--- |
| | Methylobacter tundripaludum | 307822489 | ------EF-TG-LDES---YT-R | | N-S-A------K-K---GSR-N-I--- |
| | Thiomonas sp. 3As | 294338724 | ---ARFKQ-ER-LDDT-T-AT-R | | QFS-A--H-----G---FG--A-T--- |
| | Achromobacter xylosoxidans | 311108311 | ---AK-RD-TR-LD-S---HT-A | | NFS-A--H--Q-A--ITGAR-N----- |
| | Bordetella avium 197N | 187477349 | ---AK-RD-TR-LD-S-A-HT-G | | HFS-A--H--Q-A--ITGAR-N----- |
| | Polynucleobacter necessarius | 171464095 | L---G-KD-DR-VD-T-A-YT-Q | | NF-----N--QKA--I-GSQ-N-I--- |
| | Neisseria meningitidis | 218767841 | -K-AKHKL-KR--DES-T-Y--Q | | NFS-A--H--H-AG-I-GAQ-N-I--- |
| α-Proteobacteria | Rickettsia prowazekii | 15604558 | ----K--D-KE-L-HD-K-P--- | | N--KA--H--V-IR-I-G---M-W--- |
| | Acidiphilium cryptum | 148260343 | L--ARF-AEDR-LD-A-P-L--A | | RHS-A--H--F--D---G---L-T--- |
| | Sphingobium chlorophenolicum | 334344808 | ---ARF-E--G-LD-R---PV-S | | TWS-A--H--VKAG---GA--M-Q--I |
| | Granulibacter bethesdensis | 114326954 | M--AR--D-GR--D-A-D-P--- | | RHS-A--H--F-AG---G---L-W--- |

**Figure S6**

Partial sequence alignment of a conserved region in the queuine tRNA-ribosyltransferase showing a 1 aa insert that is specific for all Xanthomonadales.
