# Supplementary material for: Phylogenomics and Molecular Signatures for Species from the Plant Pathogen-Containing Order Xanthomonadales
Source: PLoS One. 2013 Feb 8;8(2):e55216. doi: 10.1371/journal.pone.0055216 (PMC3568101; doi:10.1371/journal.pone.0055216)
Supplement: Figure S7 — Partial sequence alignment of a conserved region in the acyl-(acyl-carrier-protein)–UDP-N-acetylglucosamine O-acyltransferase showing a 1 aa insert that is commonly shared by Xanthomonadales. (PDF) [file pone.0055216.s007.pdf]

| Group | Species | Accession | 164 | | 210 |
| --- | --- | --- | --- | --- | --- |
| Xanthomonadales | Xylella fastidiosa | 71275623 | HQFCRIGRYAFIGMGTLINGDVPPFTLIGSDT | L | GRPRGINNEGLKRR |
| | Xanthomonas campestris | 78047022 | -------AH--L---A-T--------MV---S | - | -------S------ |
| | Xanthomonas fuscans | 294623975 | -------AH--L---A-T--------MV--ES | - | -------S------ |
| | Xanthomonas axonopodis | 21242162 | -------AH--L---A-T--------MV--ES | - | -------S------ |
| | Xanthomonas oryzae | 166712744 | -------AH--L---A-T--------MV-RES | - | -------S------ |
| | Xanthomonas albilineans | 285018802 | -------DH--L---A---------IMV-GNS | - | -------S------ |
| | Xanthomonas vesicatoria | 325916629 | -------AH--L---A-T--------MV--ES | - | -------S------ |
| | Xanthomonas gardneri | 325923966 | -------AH--L---A-T--------MV--ES | - | -------S------ |
| | Xanthomonas perforans | 325929588 | -------AH--L---A-T--------MV---S | - | -------S------ |
| | Pseudoxanthomonas spadix | 357417818 | -------AH------A-VG---T---MVAA-E | H | -------S---R-- |
| | Rhodanobacter sp. 2APBS1 | 352086327 | ----KV-AH------C-VGH-----VMMANEQ | Q | -------S------ |
| | Stenotrophomonas maltophilia | 190573491 | -------AH--L---A-T--------MV-T-S | - | -------S------ |
| | Stenotrophomonas sp. SKA14 | 254524358 | -------AH--L---A-T--------MV-T-S | - | -------S------ |
| Other γ-Proteobacteria | Aeromonas hydrophila | 117618038 | ---G-V-SH--V-GCAAL-K----YVMAAGNY | | AK-F-V-S---R-- |
| | Aeromonas salmonicida | 145300049 | ---G-V-SH----GCAAL-K----YVMAAGNY | | AK-F-V-S---R-- |
| | Aliivibrio salmonicida | 209695839 | ----TV-AHS---GASVVVQ-----VMAQGNH | | CK-F---I------ |
| | Alkalilimnicola ehrlichii | 114321003 | -------A---C-F-SV--R--L---TVSGHM | | AQ-H---VV--R-H |
| | Allochromatium vinosum | 288941770 | -------AH--CA--SVLTR----YVTV-GHP | | AE-H---S------ |
| | Alteromonadales bacterium | 119471155 | ----K--AH-----YSGV-K-----VTTIGMP | | AG-AA--K--M--- |
| | Alteromonas macleodii | 239992968 | ----K--AHS-L-A-GI-LR-----VMVSGQK | | NI-Q---S------ |
| | Azotobacter vinelandii | 226945929 | --H-Q--AHS-V---SGVSK---A-VTVLGSP | | AQA-SM-F--MR-- |
| | Cellvibrio japonicus | 192362392 | ----K--AHS-S---SA-GK---AYVMVNGSP | | AEAKN--A---R-- |
| | Citrobacter koseri | 157147387 | ----T--AHVMV-GCSGVAQ----YVIAQGNH | | AT-F-V-I------ |
| | Colwellia psychrerythraea | 71279846 | ----H--AHS--AGNA--LK---AYVMASGQP | | AK-F-L-S------ |
| | Congregibacter litoralis | 88706744 | -------QHS-S--Q-A-GK---AYVTVSGSP | | AEAKT--T---R-- |
| | Enterobacter cancerogenus | 288549317 | ----I--AHVMV-GCSGVAQ----YVIAQGNH | | AT-F-V-I------ |
| | Halorhodospira halophila | 121998238 | -------P---C-F-SG--R-----VTVSGQM | | AV-H---SV--R-H |
| | Idiomarina baltica | 85712983 | ----H--SH--AAVNSIVVQ-I---IMAQGHN | | A---T--S------ |
| | Klebsiella pneumoniae | 152968775 | ----V--SHVMV-GCSGVAQ-----VIAQGNH | | AT-F-V-I------ |
| | Methylococcus capsulatus | 53803395 | -------Q-S-SA--SV-SR----YVMV-GRP | | TK-H---AV--E-N |
| | Nitrococcus mobilis | 88812389 | ---S-L--HC-LAF-AHVDR----YVMAAGQR | | AT-----IK--Q-H |
| | Oceanospirillum sp. MED92 | 89092100 | ----K--SHVMC-TS-VVLK-I-AYVMANGN- | | AT-H---T------ |
| | Photobacterium profundum | 54310072 | ----TV-AHC---G-SIVVK----YVMAQGNH | | AK-F---I------ |
| | Photorhabdus asymbiotica | 253988137 | ----Q--SH-MV-GCSGVVQ----YVIAQGNH | | AT-F---V------ |
| | Proteus penneri | 226327037 | ----Q--SHVMV-GCSGVAQ-----VIAQGNH | | AT-Y-L-I------ |
| | Providencia alcalifaciens | 212710385 | ----Q--AHVMV-GCSGVAQ----YVIAQGNH | | AT-F-L-L------ |
| | Pseudomonas aeruginosa | 15598840 | --Y----AHS-S---SA-GK---AYVTVFGNP | | AEA-SM-F--MR-- |
| | Shewanella amazonensis | 119774288 | ---VH--DH--TAGCS-LLQ-----VMAAGQP | | AI---L-S------ |
| | Shigella flexneri | 110804233 | ----I--AHVMV-GCSGVAQ----YVIAQGNH | | AT-F-V-T------ |
| | Thiomicrospira crunogena | 78485618 | ----N--EHS-C---SV--Q---N-VTVSGNL | | AG---L-V------ |
| | Tolumonas auensis | 237808844 | ---G-V-SH---AGMAAL-K----YVMAAGHY | | AT-F---S---R-- |
| | Vibrio cholerae | 254225763 | ----H--DHCML-G-SIVVQ----YVMAQGNH | | CA-F---V------ |
| α-Proteobacteria | Asticcacaulis excentricus | 315499841 | ---A-----S-V-GAAMVTK--I-YGSVWGNH | | A-LE-L-LV----- |
| | Caulobacter segnis | 295689586 | ---S-----S---GLAAVTK--I-YGSVWGNH | | AHLE-L-LV----- |
| | Hyphomonas neptunium | 114799420 | ---S----N----G-AIVVE--I--GSVVGNH | | AKLS-L-IV----- |
| | Parvibaculum lavamentivorans | 154253623 | ---G-V-KH--V-GMSAVEN--I-YG-VVGNR | | AHLM-L-LI----- |
| | Nitrobacter hamburgensis | 92117252 | ---A----Q-M--GL-G-R---I-YGFVNGQH | | -HLE-L-VV-MR-- |
| | Rhodospirillum rubrum | 83592932 | ---V---KH-MV-GLSAVES--I--GSVIGNR | | AKLA-L-IV-M--- |
| | Methylocystis sp. ATCC 49242 | 323137315 | --NV---AHV---GLAGVE---I--G-A-GNR | | AHLF-V-LV-VR-- |
| | Phenylobacterium zucineum | 197105231 | ---T-V--H-M--GLAAVVK--I-YGSVWGNH | | AHLE-L-LV----- |
| β-Proteobacteria | Dechloromonas aromatica | 71907384 | -------AHVMTAVS-V-LQ----YLMAAGN- | | AQ-Y---V------ |
| | Gallionella capsiferriformans | 302878992 | ---V---AHIIT----ILLQ-----V-VSGNP | | SA-H---S------ |
| | Aromatoleum aromaticum | 56478863 | ---V-V-AHS-C-V--VLLQ-L---VTVAGNP | | AK-H---S---R-- |
| | Thiomonas intermedia | 296136567 | ---V---AHVMT-ISSV-LQ----Y--VAGNP | | AK-H---A---R-- |
| | Laribacter hongkongensis | 226941198 | ----T--AH-MVAG-SI-VQ----YVMAAGNH | | AS-V---S------ |
| | Polynucleobacter necessarius | 145589620 | ---V---QH-ML-GASALVQ-I---VIAAG-K | | AS-H---V------ |
| | Oxalobacteraceae bacterium | 329912024 | ----K--AH-MV--S-SLTQ-----VMLSGNP | | AAAH---V------ |
| | Burkholderia ubonensis | 167586867 | ---V---AH-ML-GASALVQ-----VIAAGNK | | AE-H---V---R-- |

**Figure S7**

Partial sequence alignment of a conserved region in the acyl-(acyl-carrier-protein)--UDP-N-acetylglucosamine O-acyltransferase showing a 1 aa insert that is commonly shared by all Xanthomonadales.
