# Supplementary material for: Phylogenomics and Molecular Signatures for Species from the Plant Pathogen-Containing Order Xanthomonadales
Source: PLoS One. 2013 Feb 8;8(2):e55216. doi: 10.1371/journal.pone.0055216 (PMC3568101; doi:10.1371/journal.pone.0055216)
Supplement: Figure S8 — Partial sequence alignment of a conserved region in the TolQ protein showing a 1 aa insert that is commonly shared by Xanthomonadales. (PDF) [file pone.0055216.s008.pdf]

Group	Organism	ID	177		217
Xanthomonadales	Xanthomonas campestris	21232451	GTVFGIMVTMHDM	V	SSGEQAGIAAVAPGISEALFATAIGLF
	Xanthomonas axonopodis	21243871	-------------	-	---------------------------
	Xanthomonas oryzae	166712986	-------------	-	---------------------------
	Xanthomonas albilineans	285019186	-------------	I	N--Q-----------------------
	Xanthomonas gardneri	325923400	-------------	-	---------------------------
	Xanthomonas vesicatoria	325917787	-------------	-	---------------------------
	Pseudoxanthomonas spadix	357416650	-------------	L	N--------------------------
	Pseudoxanthomonas suwonensis	319786282	-------------	-	-Q-Q-V---S-----------------
	Stenotrophomonas maltophilia	194366894	-------------	I	---A-----------------------
	Stenotrophomonas sp. SKA14	254521584	-------------	I	---A-----------------------
	Xylella fastidiosa	71275799	-------------	I	---D-------------------V---
	Rhodanobacter sp. 2APBS1	352080106	---W---GAFQGL		GEMKDVT--V---H-----I---M---
Other γ-Proteobacteria	Acidithiobacillus caldus	255020127	---W---T-FMNI		GAAQ--TL-T---PVA---I--LA---
	Alcanivorax borkumensis	110833616	---W---NSFMAL		ANVK--TLSV-----A---I-------
	Alcanivorax sp. DG881	254427665	---W---NSFMAL		ANVK--TLSV-----A---I-------
	Aliivibrio salmonicida	209695362	---W---HSFIAL		GAVK--TL-M-----A---V---M---
	Alkalilimnicola ehrlichii	114319407	---W---NSFRAL		GEMQ--TL-T-----A---I---L---
	Allochromatium vinosum	288940687	---W---QSF-AL		GNV---TL-L---------V-------
	Alteromonadales bacterium	119472452	---W---NAFIAL		GEVK--TLQM-----A---I---M---
	Azotobacter vinelandii	226945716	---W---NSFRGL		AQVQ--TLST-----A---I-------
	Cellvibrio japonicus	192362359	---W----SFLNI		ANAG-PSL-T-----A---I---M---
	Colwellia psychrerythraea	71279709	---W---NSFIAL		GAVK--TL-M-----A---I---M---
	Coxiella burnetii	29654865	---W---TSFRAL		GAVQ-VT--M---------I---V---
	Grimontia hollisae	262276106	---W---HAFIAL		GAVK--TL-M-----A---V---M---
	Hahella chejuensis	83647599	---W---NSFRGL		AHLQ--T-ST---------I---M---
	Idiomarina loihiensis	56460191	---W---NAFIAL		GAVQ--TL-M-----A---I---M---
	Legionella drancourtii	254499314	---W---TSFQAL		GHAQ--T--M---------V---L---
	Marinobacter algicola	149375114	---W---NSFRGL		AQVQ--TL-T---------I---M---
	Marinobacter aquaeolei	120554622	---W---NSFRGL		AQVQ--TL-T---------I---M---
	Marinomonas sp. MED121	87118955	---W---HSFIGL		AEVQ--TL-T-----A---I------L
	Methylococcus capsulatus	53804467	---W---NSFRSL		G-VK--TL-M---------V---M---
	Moritella sp. PE36	149910562	---W---SSFIAL		GAVQ--TL-M-----A---I---M---
	Nitrosococcus halophilus	292490338	---W---NSFRAL		GNAH--TL-M-----A---I---M---
	Oceanospirillum sp. MED92	89094840	---W---NSFRGL		ANVH--TL-S---------I-------
	Photobacterium profundum	54309714	---W---HAFIAL		GAVK--TL-M-----A---V---M---
	Pseudomonas aeruginosa	15596166	---W---NSFRGL		ATVQ--TL-T-----A---I-------
	Psychromonas ingrahamii	119944495	---W---NSFIAL		GEVQ--TLTM-----A---I---M---
	Shewanella baltica	126173972	---W---NSFIAI		G-M-N-TL-M-----A---I---M---
	Teredinibacter turnerae	254787338	---W---NSFRGL		ANVH--TL-T---------V---M---
	Vibrio cholerae B33	153824285	---W---HAFIAL		GEVK--TL-M-----A---I-------
β-Proteobacteria	Nitrosomonas eutropha	114330313	---W---NAFREL		-NVG--T--H-----A---I---M---
	Sutterella wadsworthensis	319941768	---W---NAFTGL		--L-N-SL-V-----A---V-------
	Herminiimonas arsenicoxydans	134095581	---W---NSFRGL		ANVQ--TL-------A---I-------
	Dechloromonas aromatica	71909665	---W---HAFRGL		-NVG--TL-S-----A---V-------
	Janthinobacterium sp. Marseill	152980246	---W---NAFRGL		ANVQ--TL-------A---I-------
	Herbaspirillum seropedicae	300310552	---W---NAFRGL		ANVQ--TL-------A---I-------
	Oxalobacteraceae bacterium	329907985	---W---NAFRGL		ANVQ--TLS------A---V-------
	Burkholderia multivorans	161525753	---W---NSFRGL		ANVQ--TL-N-----A---V-------
	Achromobacter xylosoxidans	338781342	---W---HAFIGL		-NMQ--TL-S-----A---I-------
	Sideroxydans lithotrophicus	291612987	---W---NAFRGL		-NVG--TL-Q-----A---V---M---
α-Proteobacteria	Methylobacterium radiotolerans	170747181	---W---TAFTSI		AASKNTSL-V-----A-----------
	Rickettsia massiliae	157964385	---W---HSFQSI		ATSNNTSL-V-----A---L-------
	Methylobacterium populi	188584488	---W---TAFTSI		AASKNTSL-V-----A-----------

Figure S8

Partial sequence alignment of a conserved region in the TolQ protein showing a 1 aa insert that is commonly shared by all Xanthomonadales
