# Supplementary material for: Phylogenomics and Molecular Signatures for Species from the Plant Pathogen-Containing Order Xanthomonadales
Source: PLoS One. 2013 Feb 8;8(2):e55216. doi: 10.1371/journal.pone.0055216 (PMC3568101; doi:10.1371/journal.pone.0055216)
Supplement: Figure S9 — Partial sequence alignment of a conserved region in alpha-2-macroglobulin domain-containing protein showing a 13 aa deletion that is uniquely present in Xanthomonadales. (PDF) [file pone.0055216.s009.pdf]

Group	Species	ID	607		661
Xanthomonadales	Stenotrophomonas maltophilia	194366795	WPANALVGVRPLFNPEDGADSNGNAR		FELMRVDAAGKPQPANGLKITLVRELRDY
	Stenotrophomonas sp. SKA14	254522704	---------------D----------		----------N----K------------
	Xylella fastidiosa	71900852	---D---------DVAS---A--M--		---T--GVD----S-K---A---------
	Xanthomonas fuscans	294627964	---K---------DD-----A--T--		--VT----D------K---V---------
	Xanthomonas campestris	289667502	---K---------DDA----A--T--		--VT----D------K---V---------
	Xanthomonas axonopodis	21241899	---K---------DDG----A--T--		--VT----D------K---V---------
	Xanthomonas perforans	325925285	---K---------DDA----A--T--		--VT----D------K---V---------
	Xanthomonas vesicatoria	325915165	---K---------DDK----A--T--		---T----D------K---A---------
	Xanthomonas gardneri	325922888	---K---------DDK----A--T--		--IT----D------K---A---------
	Pseudoxanthomonas spadix	357417253	---AV--------DDKQ-S-A--T-G		--IA-M--T--RLSGQ---V-----R---
	Pseudoxanthomonas suwonensis	319786934	---P---------E-A---PAES--G		---V----Q------R--EL---------
	Rhodanobacter sp. 2APBS1	352080521	---D---------D-KQ--E-EAP-G		--IV-AN-N-ERVAGAH--VR-Q-----F
Other Bacteria	Citrobacter koseri	157144543	--TDT-P-I--Q-ASKAVY-YRTD-T	VNQPIVDEGGNAA	-DIVYA--Q-NKKA-S--QVR-I--R---
	Cronobacter sakazakii	156932934	---D----I--Q-GEKAVY-YRTDDT	VSQPMVDENGNAG	-DIVYAN-Q-EKVAVK---VR----R---
	Enterobacter cancerogenus	261340828	---A--P-I--Q-ASKAVY-YRTDTT	VNQPIVDENGNAS	-DIVYA--T-AKKAVS--QVR----R---
	Escherichia coli	293415788	---D--P-L--Q-ASKSVY-YRTDST	VKQPIVDEGSNAA	-DIVYS--Q-VKKAVS--QVR-I--R---
	Klebsiella pneumoniae	206580715	---G--P-I--Q-TLK-VY-YRTDTT	VKQPVVDENSNAA	-DIVYA--K-EKKAVS--QVR-I--R---
	Photorhabdus asymbiotica	253988861	---EK---I----GQKQVYNYRTGRD	QSRYNVDENSLAE	---VYA--N-EKYA-KD-TAR--Y-R---
	Proteus mirabilis	227356217	---TH-A-I--V-AKKEVY-YRNDKY	KSGYSVDENSMAE	--IVYT-QE--KL--DD--VRFIY-RY--
	Providencia alcalifaciens	212710621	---KK---I----EKKEIY-YRTDRY	AKRYNVDENSLAE	--IVMS-M--HKLA-EN-TAR--Y-R---
	Pseudomonas syringae	302185183	---EH-P---A--GSSTSS-DYDDEA	KGEAQTNGDGPAD	--IVMA----NKLA-DNV-VR----R---
	Salmonella enterica	161502313	---DT-P-I--Q-TAKAVY-YRTD-T	VNQPIVDEDSNAA	-DIVYAN-Q-EKKAVS--QVR-I--R---
	Serratia proteamaculans	157371850	---D--P-I-----KQQIY-YRS-SY	QAQPVVDENTQAG	-DIVYAN---EKLAVS---AR----R---
	Shigella boydii	187733068	---D--P-L--Q-ALKSVY-YRTDST	VKQPIVDEGSNAA	-DIVYS--Q-VKKAVS--QVR-I--R---
	Xenorhabdus bovienii	290476010	---EK-A-I-----KKSIYNYRTGHD	ENRYNVDENSLAE	-DVVYA--S--KYAT-D-RTR-IY-R---
	Xenorhabdus nematophila	300724160	---KQ---------KKTIYNYSTGKD	ENRYNVDENSQAE	--IAYA--K--KYATSE-RAR-IY-R---
	Yersinia aldovae	238758827	---D--A-I-----KQQVY-YRSDSY	KSQAMVDQDTTAD	--IIYAN-S--KLA-D---VK----R---
	Yersinia mollaretii	238797957	---D--A-I-----KQQVY-YRSDSY	KSQAMVDQDTQAD	--IVYAN-E-EK-AVE---VK-I--R---

Figure S9

Partial sequence alignment of a conserved region in alpha-2-macroglobulin domain-containing protein showing a 13 aa deletion that is uniquely present in all Xanthomonadales.

Alpha and beta have 1-5 aa deletion at the same position
