# Supplementary material for: Phylogenomics and Molecular Signatures for Species from the Plant Pathogen-Containing Order Xanthomonadales
Source: PLoS One. 2013 Feb 8;8(2):e55216. doi: 10.1371/journal.pone.0055216 (PMC3568101; doi:10.1371/journal.pone.0055216)
Supplement: Figure S10 — Partial sequence alignment of a conserved region of DNA topoisomerase IV subunit B showing a 1 aa deletion that is commonly specifically found in Xanthomonadales. (PDF) [file pone.0055216.s010.pdf]

```
                                                                               282                                   326
                 Xanthomonas oryzae              84624476     FVGSLKKDTEIVDWAAGWVPE   GELVQESYVNLIPTAQHGTHVNGL
                 Xanthomonas campestris          289662651    ----------------A----   ------------------------
                 Xanthomonas fuscans             294663738    ----------------A----   ------------------------
                 Xanthomonas axonopodis          21242463     ----------------A----   ------------------------
                 Xanthomonas gardneri            325924529    -A--------------A----   ------------------------
                 Xanthomonas vesicatoria         325916059    -A--------------A----   ------------------------
Xanthomonadales  Xanthomonas albilineans         285017938    --------R-------A--VD   -D----------------------
                 Pseudoxanthomonas spadix        357417807    ---G---Q-------LA----   --------------P---------
                 Pseudoxanthomonas suwonensis    319786497    -S-A---E--V----VA-I--   ------------------------
                 Stenotrophomonas maltophilia    190573692    ---N------V----VA-L--   ------------------------
                 Stenotrophomonas sp. SKA14      254524349    ---N------V----VA-L--   ------------------------
                 Xylella fastidiosa              15837887     -I-C------V----VA----   ---T----------P---------
                 Rhodanobacter sp. 2APBS1        352081276    --HQMAR-I-GMEV-LA-L--   ----------------G-------
                 Aeromonas hydrophila            117618295    ---AFSTEQSA----LC-L-- G --CLA-----------G-------
                 Aeromonas salmonicida           145297604    ---AFSTEQSA----LC-L-- G --CLA---------P-G-------
                 Aggregatibacter aphrophilus     251792940    -I-EF-GEK-A-S--LL-L-- G ---IG----------LG-------
                 Alcanivorax borkumensis         110835355    -N-AMSAE--A----VT-L-- G ------A---------G-------
                 Alcanivorax sp. DG881           254427031    -N-AMSAE--A----VT-L-- G ---I--A---------G-------
                 Alkalilimnicola ehrlichii       114320108    -I-RMSS-H-AAE--VT-L-- G --AIT-----------G-------
                 Alteromonas macleodii           196158482    ---NFTGN--AA---VM-L-- G -D--T-----------G-------
                 Arsenophonus nasoniae           284008571    ---NFAGQL-M----LL-L-- G ---LT-----------G-------
                 Azotobacter vinelandii          226946371    -T--FAASR-A-E--LL-L-- G --FRS---------D-G-------
                 Candidatus Regiella             304413731    -I-TFSAA--AI---LL-L-- G ---LT---------I-G-------
                 Cellvibrio japonicus            192360165    ---DF-AE--AIS--VQ-L-- G ----------------G-------
                 Chromohalobacter salexigens     92114662     -L-HFEDEDQG----IQ-L-- G ---L----------PLG-------
                 Congregibacter litoralis        88705315     -T--FSGS--A----VQ-L-- G ---IT-----------G-------
                 Cronobacter sakazakii           156932596    -I-NFSGE--A----LL-L-- G ---LT---------M-G-------
                 Dickeya dadantii                242241013    -T-TISG---A-E--LL-L-- G ---LT---------P-G-------
                 Edwardsiella ictaluri           238918166    -T-TFAS---A----LL-L-- G ---LT---------P-G-------
                 Enterobacter cloacae            296104690    ---NFTG---A----LL-L-- G ---LT---------MLG-------
                 Erwinia tasmaniensis            188532591    ---TFSG-V-A----LL-L-- G ---LT---------M-G-------
                 Escherichia albertii            170765612    -I-NFNGE--A----LL-L-- G ---LT---------M-G-------
                 Grimontia hollisae              262274994    ---EFSASN-AA---VI-L-- G ---IT-----------G-------
                 Haemophilus influenzae          145629698    ---EF-GAN-A-S--LL-L-- G ---IG---------I-G-------
                 Idiomarina loihiensis           56461054     -D--FSSQ--G----VT-L-- G --G-S---------TLG-------
Other Bacteria   Kangiella koreensis             256823223    -T---QGEA-A----FM-L-- G --CLA---------P-G-------
                 Mannheimia haemolytica          254362507    -I-DVTAE--A-S--LT-L-- G ----A-----------G-------
                 Nitrosococcus oceani            77165746     -T-EMQGN--E-C--FT-L-- G --V-T---------P-G-------
                 Oceanobacter sp. RED65          94500377     -E--MSGN--A----VQ-L-- G --QLM-----------G-------
                 Pantoea ananatis                291618921    ---TFAG-I-A----LL-L-- G ---LT---------M-G-------
                 Pasteurella multocida           15602235     -I-DF-G---A-S--LL-L-- G -D-LS---------I-G-------
                 Pectobacterium carotovorum      227112782    -L--ITG---A----LL-L-- G ---LT---------PMG-------
                 Photobacterium damselae         269103508    -T--FSGQ--AA---LL-L-- G ---IT-----------G-------
                 Photorhabdus asymbiotica        253991001    ---AFSGE--A----LL-L-- G ---LA---------M-G-------
                 Proteus mirabilis               197286191    -T-KFEG-N-A-E-SML-L-- G -D-LT---------V-G-------
                 Providencia stuartii            183597858    -T-EFSGES-AAE--LL-L-- G ---LT-----------G-------
                 Pseudomonas aeruginosa          4176381      -C-N-EGSK-A-S--LL-L-- G --S-------------G-------
                 Reinekea sp. MED297             88797038     -T--FSS-E-A----VQ-L-- G --GLF---------P-G-------
                 Saccharophagus degradans        90020023     ----FTGT--AA---VQ-L-- G --VT------------G-------
                 Salmonella enterica             161506253    -I-NFNGE--A----LL-L-- G ---LT---------M-G-------
                 Serratia odorifera              270263149    -I-NFAG---A----LL-L-- G ---LT---------M-G-------
                 Shewanella benthica             163749330    -I---QGNK-A----IT-L-- G --YLN---------PLG------F
                 Shigella flexneri               30064380     -I-NFAG---A----LL-L-- G ---LT---------M-G-------
                 Shigella sonnei                 74313568     -I-NFAG---A----LL-L-- G ---LT---------M-G-------
                 Sodalis glossinidius            85058249     ---AVSG---T---VLL-L-- G ---LT---------P-G-------
                 Teredinibacter turnerae         254787783    ----FSAA--AA---VQ-L-- G --IT------------G-------
                 Thioalkalivibrio sp. K90mix     289209261    -M--VSGA--AA---VT-L-- G --V-S---------T-G-------
                 Tolumonas auensis               237807171    -T-QFSA-NSA----LC-L-- G ---IS---------V-G-------
                 Yersinia kristensenii           238762440    ----FAG---AI---LL-L-- G ---LT---------M-G-------
```

Figure S10

Partial sequence alignment of a conserved region of DNA topoisomerase IV subunit B showing a 1 aa deletion that is commonly present in all Xanthomonadales.
