# Supplementary material for: Phylogenomics and Molecular Signatures for Species from the Plant Pathogen-Containing Order Xanthomonadales
Source: PLoS One. 2013 Feb 8;8(2):e55216. doi: 10.1371/journal.pone.0055216 (PMC3568101; doi:10.1371/journal.pone.0055216)
Supplement: Figure S11 — Partial sequence alignment of DNA polymerase I showing a 1 aa deletion that is uniquely shared by all members of Xanthomonadales. (PDF) [file pone.0055216.s011.pdf]

			28		65
Xanthomonadales	Stenotrophomonas maltophilia	194367713	GEPTGALFGVVNMLRSTLKE		RPAYVAFVVDAPGKTFRD
	Stenotrophomonas sp. SKA14	254524679	--------------------		------------------
	Xanthomonas campestris	78049756	---------------A----		------------------
	Xanthomonas fuscans	294626790	---------------A----		----I-------------
	Xanthomonas axonopodis	21244827	---------------A----		----I-------------
	Xanthomonas albilineans	285016940	---------------A----		--E---------------
	Xanthomonas oryzae	58583863	---------------A----		----I-------------
	Xanthomonas vesicatoria	325917393	---------------A----		------------------
	Xanthomonas gardneri	325923784	---------------A----		------------------
	Xanthomonas perforans	325924860	---------------A----		----I-------------
	Pseudoxanthomonas spadix	357415770	---------------A----		--E-I---I---------
	Pseudoxanthomonas suwonensis	319788387	---------------A----		K-D---------------
	Rhodanobacter sp. 2APBS1	352086477	--A------------A---A		K-D-L---S----P----
	Xylella fastidiosa	71276059	---------------T----		--D-A---I---------
Other γ-Proteobacteria	Actinobacillus succinogenes	152977709	------M---L---K-LISQ	V	--SHI-V-F--K------
	Aggregatibacter aphrophilus	251793585	------MY--L---K-LISQ	V	Q-SHI-V-F--K------
	Alcanivorax borkumensis	110835520	-Q----VR--AS---KLI-D	Y	D-Q-M-VIF--K------
	Aliivibrio salmonicida	209693839	------VY--I-----L--Q	F	ETNRI-VIF--K-----N
	Alkalilimnicola ehrlichii	114322007	------MY--L--V-KL-DD	Y	--ERI-V-F----R----
	Alteromonas macleodii	196154841	-QD---IY------K-LI-Q	Y	N-THM-VIF--K------
	Arsenophonus nasoniae	284009008	------MY--L-----LIMQ	Y	K-SHI-V-F--K------
	Cellvibrio japonicus	192361143	-Q----VK-------RLR-D	Y	PQSPI-V-F--K------
	Chromohalobacter salexigens	92112681	-N----VK--L---K-LI-Q	Y	PQSPM-V-F--K------
	Citrobacter rodentium	283787470	------MY--L-----LIMQ	Y	Q-THA-V-F--K------
	Congregibacter litoralis	88705360	-AA---VK--I--I-RLQ-D	Y	PESTI-V-F--K------
	Cronobacter sakazakii	156936143	------MY--L-----LILQ	Y	Q-THA-V-F--K------
	Dickeya dadantii	307128781	------MY--L-----L-QQ	Y	H-SH--V-F--K------
	Edwardsiella tarda	269140843	------MY--L-----L-MQ	Y	Q-SH--V-F--K------
	Enterobacter cancerogenus	261342936	------MY--L-----LILQ	Y	Q-THA-V-F--K------
	Erwinia tasmaniensis	188532189	------MY--L-----L-LQ	Y	K-SH--V-F--K------
	Escherichia coli	300925951	------MY--L-----LIMQ	Y	K-THA-V-F--K------
	Grimontia hollisae	262273193	------VY--------LV-Q	Y	PQSH--VIF--K------
	Haemophilus ducreyi	33152330	------MY--L-----LVNQ	V	N-SHI-V-F--K------
	Halorhodospira halophila	121999199	-------Y------HKL-A-	E	PE-RF-V-F---------
	Idiomarina loihiensis	56459137	------IY--------L--R	Y	K-SHM-V-F--K-----S
	Mannheimia haemolytica	254360483	------MY--L---K-LISQ	V	N-SHI-V-F--K------
	Methylococcus capsulatus	53803295	------VY--I----KL-QT	Y	DG-HI-V-F----RN---
	Pasteurella dagmatis	260912940	N-----MY--L---K-LISQ	V	Q-SHI-V-F--K------
	Pectobacterium wasabiae	261823708	--A---MY--L-----L-QQ	Y	S-SH--V-F--K------
	Photobacterium profundum	90414265	------VY--------L-RQ	F	STEHI-VIF--K------
	Photorhabdus asymbiotica	253987782	------MY--L-----LVMQ	Y	K-SH--V-F--K------
	Proteus penneri	226327657	------MY--L-----LIIQ	Y	K-SH--V-F--K------
	Providencia alcalifaciens	212711548	------MY--L-----LIMQ	Y	T-SH--V-F--K------
	Rickettsiella grylli	160872753	------IY--IS---KLM--	N	D-E-IVV-F--K-----E
	Saccharophagus degradans	90019722	-Q----VR-------KLA-D	Y	PESPI-VIF--K------
	Salmonella enterica	161505500	------MY--L-----LIMQ	Y	Q-THA-V-F--K------
	Serratia proteamaculans	157373118	------MY--L-----L-LQ	Y	Q-SH--V-F--K------
	Shewanella oneidensis	24376141	--A---VY--------L-SR	Y	Q-SHI-V-F--K-----N
	Shigella dysenteriae	194433216	------MY--L-----LIMQ	Y	K-THA-V-F--K------
	Sodalis glossinidius	85060213	------MY--L---K-L-VQ	Y	--SH--V-F--K------
	Teredinibacter turnerae	254784333	-Q----IK-------RLE-D	Y	PESPI-V-F--K------
	Xenorhabdus bovienii	290477056	------MY--L-----LIIQ	Y	K-SH--V-F--K------
	Yersinia pestis	262364150	------MY--L-----L-LQ	Y	--SH--V-F--K------
α-Proteobacteria	Rickettsia canadensis str. McK	157804146	-K-V---Y-FTS--LKL-SD	F	K-KH--V-F-SG--N--H
	Anaplasma marginale str. Missi	254995381	-A-I-GVY-FI-I-LKH-LT	H	NAD-I-V-F-TGS-N--H
	Candidatus Puniceispirillum	294084835	-T-VN-VY-FTS--MKLVDD	M	M-DH--V-F-TSRQ---S
β-Proteobacteria	Achromobacter xylosoxidans	311105001	-------Y-------KLVSD	H	KAE-A-CIF--R------
	Nitrosomonas europaea	30249439	N-----IY--L----RLH--	Y	--D-S-C-F--K------
	Achromobacter xylosoxidans	317402839	-------Y-------KLVSD	Y	KAE-A-CIF--R------
	Bordetella petrii	163857439	-------Y-------KLVSD	H	KAD-A-CIF--R------
	Methylobacillus flagellatus	91774714	------MY--L----RLH-D	Y	PAD-S-C-F--K------
	Bordetella bronchiseptica	33602215	-------Y-------KLVQD	H	KAE-AVC-F--R------

Figure S11

Partial sequence alignment of a conserved region of DNA polymerase I showing a 1 aa deletion that is uniquelly shared by all members of Xanthomonadales.
