# Supplementary material for: Phylogenomics and Molecular Signatures for Species from the Plant Pathogen-Containing Order Xanthomonadales
Source: PLoS One. 2013 Feb 8;8(2):e55216. doi: 10.1371/journal.pone.0055216 (PMC3568101; doi:10.1371/journal.pone.0055216)
Supplement: Figure S12 — Partial sequence alignment of a conserved region of aromatic amino acid aminotransferase showing a 1 aa deletion that is uniquely shared by Xanthomonadales. (PDF) [file pone.0055216.s012.pdf]

			306		354
Xanthomonadales	Xylella fastidiosa	28197970	LRTLWEQELTQMRERIHGLRAGLVARLKALGAPEFD		FIQRQAGMFSYSG
	Xanthomonas campestris	21229575	--DV------E------A-------K-A-------E		-------------
	Xanthomonas axonopodis	21240899	--D-------E------S------EK-AT-------		-------------
	Xanthomonas fuscans	294625499	--D-------D------S------EK-AT-------		-------------
	Xanthomonas oryzae	58424233	--DV------E------A------EK-AT------E		-------------
	Xanthomonas albilineans	285016839	--AM------E------A----M-QK-A-------G		---Q---------
	Xanthomonas vesicatoria	325918790	--DV------E------A------EK-AT------A		-------------
	Xanthomonas perforans	325926773	--D-------E------S------EK-AT-------		-------------
	Xanthomonas gardneri	325920984	--D-------E------A-------K-AT-------		-------------
	Rhodanobacter sp. 2APBS1	352086152	--AR--E--GG--G---AM---F-EK-A-----D-G		--N----------
	Pseudoxanthomonas suwonensis	319785658	--G---D--R-------A-------K-AEQ-R---A		--SQ---------
	Pseudoxanthomonas spadix	357415998	--AD--T---A------A------QK-ADA--GD-S		--A----------
	Stenotrophomonas maltophilia	194363799	--AM------E------A--Q---EK-A-A---Q-G		--NE---------
	Stenotrophomonas sp. SKA14	254524792	--AM------E------A--Q---EK-A-A---Q-G		--NE---------
Other γ-Proteobacteria	Aliivibrio salmonicida	209695181	---E----VQE--A--QEM-TLF-TT--EQ-VDANF	S	--E--N----F--
	Azotobacter vinelandii	226944117	--A---S--AE--G--QSM-QAM-EQ-A-Q--KRDF	S	-VA--R-------
	Chromohalobacter salexigens	92113446	-A-V-RE---E--D--N---RDF-EA--PY-LD-RF	A	--AE-R-----T-
	Citrobacter koseri	157146380	--AI------D--Q--QRM-LLF-NT-QEK--DRDF	S	--AK-N----F--
	Cronobacter turicensis	260597328	---M------D--Q--QRM-QLF-NT-AEK--NRDF	S	--TK-N----F--
	Dickeya dadantii	307131493	--NI------A-----QRM-HLF-ST-QEK--NQDF	S	--IN-N----F--
	Edwardsiella tarda	294635623	---M------D--Q--QRM-QLF-NT-QEK--EQDF	S	--TQ-N----F--
	Erwinia billingiae	300716099	--AI-----AD--Q---RM-QLF-NT-QEK--KGDF	S	--IK-N----F--
	Escherichia albertii	170769330	--AI------D--Q--QRM-QLF-NT-QEK--NRDF	S	--TK-N----F--
	Escherichia coli	297520301	--AI------D--Q--QRM-QLF-NT-QEK--NRDF	S	--IK-N----F--
	Klebsiella pneumoniae	262040972	--AI------D--Q--QRM-LLF-NT-QEK--SRDF	S	--SQ-N----F--
	Klebsiella variicola	288936274	--AI------D--Q--QRM-LLF-NT-QEK--SRDF	S	--SQ-N----F--
	Marinomonas sp. MED121	87121047	--S---M--ES--K---EM-SLF-ST-R-N-VEQDF	S	--S--Q----F--
	Pantoea sp. At-9b	258636143	---I------D--Q--QRM-QLF-NT-ASS--EQDF	S	--IK-N----F--
	Photobacterium damselae	269102889	--AE----VAE--D--QEM-TLF-DT--S--IDRDF	S	--EQ-N----F--
	Photorhabdus asymbiotica	253990243	-KAV------T-----QRM-QLF-NT-QEK--NQDF	S	--IN-N----F--
	Pseudoalteromonas tunicata	88860457	-TA--H---AE--D--N---TLI-DS-N-Q-IDQDF	S	--KH-F----FL-
	Pseudomonas aeruginosa	20559868	--A------GE--D--RDM-LAM-EQ-A-H--KRDF	S	-VG--R-------
	Reinekea sp. MED297	88797991	-KAK-I--VAD--QH-----KLM-ET--D--VEKDF	S	---A-N----F--
	Salmonella enterica	161503873	--AI------D--Q--QRM-QLF-NT-QEK--NRDF	S	--IK-N----F--
	Serratia odorifera	270261211	--A-------D--Q---RM-QLF-NT-QEK--EQDF	S	--IN-N----F--
	Shewanella woodyi	170726758	-KA---R--KE-----AKM-TLF-ET--SE-VTQDF	S	--S--N----F--
	Shigella flexneri	281600288	--AI------D--Q--QRM-QLF-NT-QEK--NRDF	S	--IK-N----F--
	Sodalis glossinidius	85060109	-N---RE-VAA--Q--LAM-HS---A--VALPDHQF	D	YLL--R-----T-
	Vibrio alginolyticus	91227221	M-AE--A-VAE--D--QEM-ELF--T---E-VDADF	S	--E--N----F--
	Vibrio cholerae	229515668	--AE--A-VAE-----QEM-VLF-NT--EV-VDADF	S	--E--N----F--
	Yersinia pestis	108806701	--AI------D--Q--QRM-QLF-NT-QEK--QQDF	S	--IN-N----F--
	Yersinia ruckeri	238754324	--AI------D--Q---RM-QLF-KT-QEK--KQDF	S	--IN-N----F--
α-Proteobacteria	Beijerinckia indica	182680498	-TA--HD--AL--D--KAM-E--AE--RVQVPDRDF	S	--T--R-------
	Maritimibacter alkaliphilus	84684244	---E--T--EEV-NTML---TQ-AEA-Q-RTGSDRF	G	-LAEHR----RL-
	Sagittula stellata	126730888	--AD--E-VREI--GVL---EQ-A-E-QR-TGSDRF	G	--AQHR----RL-
β-Proteobacteria	Methyloversatilis universalis	334129888	--SM--E--GE-----RAM-T---DK-S-R-VARDF	S	-VV--R-----T-
	Burkholderia xenovorans	91782671	--AT--T--AE--D--RAM-N---E----S-VDRDF	S	-VNA-R-------
	Verminephrobacter eiseniae	121609327	--A---R--A---L--KAM-QQ---G---A-VRQDM	D	--N--I-------

Figure S12

Partial sequence alignment of a conserved region of aromatic amino acid aminotransferase showing a 1 aa deletion that is uniquelly shared by all Xanthomonadales.
