# Supplementary material for: Phylogenomics and Molecular Signatures for Species from the Plant Pathogen-Containing Order Xanthomonadales
Source: PLoS One. 2013 Feb 8;8(2):e55216. doi: 10.1371/journal.pone.0055216 (PMC3568101; doi:10.1371/journal.pone.0055216)
Supplement: Figure S13 — Partial sequence alignment of a conserved region of DNA polymerase III subunit beta showing a 1 aa deletion that is present in Xanthomonadales. The CSI has also been found to be shared by Marinomonas sp. MWYL1 and Thioalkalivibrio sp. HL-EbGR7. (PDF) [file pone.0055216.s013.pdf]

Group	Species	GI	44		81
Xanthomonadales	Stenotrophomonas maltophilia	194363780	LTGTDLEVEMVSRIAVED		AQDGETTIPARKLFEIIRAL
	Xanthomonas albilineans	285016823	----------------D-		----------------V---
	Xanthomonas campestris	21229480	----------I--TM---		--------------D-L---
	Xanthomonas oryzae	166709897	----------I--TM---		--------------D-L---
	Xanthomonas fuscans	294665123	----------I--TL---		--------------D-L---
	Xanthomonas axonopodis	21240776	----------I--TL---		--------------D-L---
	Xanthomonas vesicatoria	325915700	----------I--TM---		--------------D-L---
	Xanthomonas gardneri	325922760	----------I--TM---		--------------D-L---
	Pseudoxanthomonas suwonensis	319785622	----------IA-A----		-V---I----------V---
	Pseudoxanthomonas spadix	357415873	----------IA-S----		----------------V---
	Rhodanobacter sp. 2APBS1	352080695	-----------ATTEA-K		LV---V--------D-V---
	Xylella fastidiosa Dixon	71275533	----------I-KTII--		-ES--I------IY--V---
	Xylella fastidiosa 9a5c	15836607	----------I-KTII--		-ES--I------IY--V---
	Xylella fastidiosa Temecula1	28197947	----------I-KTKI--		PES--I------IY--V---
→	Marinomonas sp. MWYL1	152994045	---S-----L-GHVPLDE		CEE-RI-V-----MD-CKS-
	Thioalkalivibrio sp. HL-EbGR7	220933195	V--------L-AEASEVS		-SP--A-V-----LD-C---
Other Bacteria	Aeromonas hydrophila	117619320	----------IGQVYLNS	D	S---R--V-----LD-C-G-
	Alteromonas macleodii	196154828	---------LI-SVKL-G	E	FTE--I-V--K---D---GI
	Idiomarina loihiensis	56459114	---------L--AVPL-S	A	DM-AAV-V--K--LD-V-S-
	Marinobacter aquaeolei	120552946	----NM---L-A-V-PVH	V	D-P-RI-V-----AD-C---
	Psychromonas ingrahamii	119947309	---------LIAV--L-N	P	SEN-VI-V-----LD-C-G-
	Shewanella amazonensis	119773156	M--------L-GEAT-HG	D	V-E-R--V--K--LD-VKS-
	Teredinibacter turnerae	254784275	---------IIG-VQL-S	A	PGN--I-V--K-FLD-C-S-
	Azotobacter vinelandii	226942172	---------L-G-VPL-E	N	-EP--I-V-----MD-CKS-
	Cellvibrio japonicus	192362292	---------IIG-LTL-Q	A	G-S--I-V-----VD-C-S-
	Pseudomonas aeruginosa	15595200	---------L-G-VVL--	A	-EP--I-V-----MD-CKS-
	Arsenophonus nasoniae	284007064	-----------AKVTLTQ	P	GES-SI-V----F-D-W-G-
	Citrobacter koseri	157144343	-------M---A-V-LIQ	P	HEP-A--V----F-D-C-G-
	Cronobacter sakazakii	156936081	-------M---A-V-LVQ	P	HEP-A--V----F-D-C-G-
	Dickeya zeae	251787654	-------M---AHL-LTQ	P	HEP-A--V------D-C-G-
	Edwardsiella ictaluri	238917985	-------M---A-VPLVQ	P	HEA-A--V----FLD-C-G-
	Enterobacter cloacae	296100373	-------M--IA-VTLTQ	P	HDA-A--V----F-D-C-G-
	Erwinia amylovora	292490135	-------M---A-V-LAQ	D	H-P-A--V----F-D-C-G-
	Escherichia coli	284923784	-------M---A-V-LVQ	S	HEP-A--V----F-D-C-G-
	Klebsiella pneumoniae	152972608	-------M---A-V-LVQ	P	HEA-A--V----F-D-C-G-
	Morganella morganii	88697472	-------M--MAAVPLDQ	P	HEN----V----FLD-W-G-
	Pantoea ananatis	291615642	-------M---A-V-LTQ	P	HET-A--V----FLD-C-G-
	Pectobacterium carotovorum	253686411	-------M---AKV-LTQ	P	HEP-A--V------D-C-G-
	Photorhabdus asymbiotica	253987485	-------M--MA-VTLSL	P	HEE-A--V----F-D-W-G-
	Proteus mirabilis	197286951	-------M--MA-VSLSQ	S	HEI-A--V----F-D-W-G-
	Providencia rettgeri	268593489	-------M--MANV-LTG	E	HEI-A--V----F-D-W-G-
	Salmonella enterica	16762488	-------M---A-VTLSQ	P	HEP-A--V----F-D-C-G-
	Serratia odorifera	270264120	-------M---A-V-LSQ	P	HEA-A--V----F-D-C-G-
	Shigella flexneri	30065009	-------M---A-V-LVQ	P	HEP-A--V----F-D-C-G-
	Sodalis glossinidius	85057980	-------M---AQV-LSA	A	HEP-A--V----F-D-C-G-
	Xenorhabdus nematophila	300721091	-------T--KA-VTLTQ	P	HEQ-A-------F-D-W-G-
	Yersinia aldovae	238759585	-------M---A-V-LSH	P	HEQ-A--V----F-D-W-G-
	Yersinia pestis	22127980	-------M---ACV-LSQ	S	HEP-A--V----F-D-W-G-
	Alcanivorax borkumensis	110832863	M------L-L-A-LPL-G	E	LEP----V-----VD-GKS-
	Oceanospirillum sp. MED92	89092265	M--------L-G-VTLDE	P	-EA-SV-V-----MD-CKS-
	Vibrio furnissii	260771048	M-A------L---VTL-G	D	FEA-T--V----FLD-C-G-

Figure S13

Partial sequence alignment of a conserved region of DNA polymerase III subunit beta showing a 1 aa deletion that is present in all Xanthomonadales. The CSI has also been found to be shared by *Marinomonas sp. MWYL1* and *Thioalkalivibrio sp. HL-EbGR7*.
