# Supplementary material for: Phylogenomics and Molecular Signatures for Species from the Plant Pathogen-Containing Order Xanthomonadales
Source: PLoS One. 2013 Feb 8;8(2):e55216. doi: 10.1371/journal.pone.0055216 (PMC3568101; doi:10.1371/journal.pone.0055216)
Supplement: Figure S14 — Partial sequence alignment of a conserved region of lipid-A-disaccharide synthase a 2 aa insert that is present in Xanthomonadales. The CSI has also been found to be shared by Cardiobacterium hominis, Allochromatium vinosum and Alteromonadales bacterium. (PDF) [file pone.0055216.s014.pdf]

Group	Species	Accession	317		358
Xanthomonadales	Stenotrophomonas maltophilia	190573490	PMVVGYRVNELTYRLVKA	LG	LIKVDRFALPNILAGQDLAPEL
	Stenotrophomonas sp. SKA14	254521713	------------------	--	----------------------
	Xanthomonas albilineans	285018803	------K-AP----I---	--	-L----Y--------H------
	Xanthomonas oryzae	166712745	------K-AP----I--L	--	----N-Y-------ND------
	Xanthomonas axonopodis	21242161	------K-AP----I--L	--	-L--N-Y-------ND------
	Xanthomonas campestris	289662894	------K-AP----I--L	--	-L--N-Y-------ND------
	Xanthomonas fuscans	294625961	------K-AP----I--L	--	-L--N-Y-------ND------
	Xanthomonas vesicatoria	325916628	------K-AP----I--T	--	-L--N-Y-------ND------
	Xanthomonas gardneri	325923965	------K-AP----I--T	--	-L--N-Y-------ND------
	Pseudoxanthomonas spadix	357417819	--------AP-SAWIART	--	-L--E------V----------
	Pseudoxanthomonas suwonensis	319786396	--------AP--------	--	-L----Y--------K------
	Xylella fastidiosa	15837644	----A-K-AP----I--T	-K	-L-IN----------E------
	Rhodanobacter sp. 2APBS1	352086326	--------AP-S--IAL-	-K	ML-T-IY-------
	Cardiobacterium hominis	258546185	----A---HPVSAAIAR-	-R	-L-IN--S---L---A-IV--C
	Allochromatium vinosum	288941771	----T--LHP---HV--W	-K	-V--PYV-MA-L---RA----F
	Alteromonadales bacterium	119471148	------KIKP-S-WIF-T	-F	TFNIKY-S---L--DEE-V--F
Other Bacteria	Acinetobacter radioresistens	255318109	---TF-KL-W---IIA-L		-V-IPYYS----I--KKVIA--
	Actinobacillus minor	257464952	------KMKPM--W-A-K		-V-T-YIS---L--NEP-V---
	Actinobacillus succinogenes	152979548	-------MKPF--F-A-R		-V-TNYIS---L--NEM-V--M
	Aeromonas hydrophila	117617831	------KLKPFS-W-AQR		-V-TEFVS---L---RM-V---
	Aeromonas salmonicida	145300048	------KLKPFS-W-AQR		-V-TAYVS---L--D-M-V---
	Aggregatibacter aphrophilus	251793244	-------MKPF--F-A-R		-V-TKYVS---L--DEM-V---
	Citrobacter rodentium	283783966	-------MKPF-FW-A-R		-V-T-YVS---L---RE-VK--
	Cronobacter turicensis	260596602	-------MKPF-FW-A-R		-V-TEFVS---L---RE-VK--
	Dickeya zeae Ech1591	251790732	-------MKPF--W-A-R		-V-TPWVS---L---RE-VS--
	Edwardsiella ictaluri	238918788	----A--MKPF-FW-AQR		-V-TEFVS---L---RE-V---
	Enterobacter cancerogenus	261338820	-------MKPF-FW-A-R		-V-T-YVS---L---RE-VK--
	Erwinia amylovora	292489214	-------MKPF-FW-A-R		-V-T-YVS---L---RE-V---
	Escherichia coli	170681777	-------MKPF-FW-A-R		-V-TEYVS---L---RE-VK--
	Grimontia hollisae	262276514	------K--AI-AWIARR		ML-TEFVS-------RE-V--R
	Haemophilus influenzae	260581886	-------MKP---F-A-R		-V-T-YIS---L--NEM-V--M
	Hahella chejuensis	83647903	----A-KLAT-S-WIMRR		-L-AKYIS---L--DKA-V---
	Kangiella koreensis	256823114	-T--A-K-GGFS-QIF-R		-LII-T--I--L--KKP-I---
	Klebsiella pneumoniae	206580293	-------MKPF-FW-A-R		-V-T-YVS---L---RE-VK--
	Mannheimia haemolytica	254361154	-------MKP---W-A-K		-V-T-YIS---L--QAP-V---
	Pantoea ananatis	291616359	-------MKPF-FW-A-R		-V-T-YVS---L---RE-VK--
	Pasteurella dagmatis	260913167	-------MKST--F-A-R		-V-T-YIS---L--NEM-V--M
	Providencia rettgeri	268590519	-------MKPF-FW-A-R		-V-TPYVS---L---KEIVK--
	Psychromonas ingrahamii	119946584	----A-K--L---VIA--		-V--KYTS---LI-DKEIVK--
	Saccharophagus degradans	90022229	---IA-HMAAFS-W-LSK		-V-SKFVG---L--DKE-V---
	Salmonella enterica	56412502	-------MKSF-FW-A-R		-V-TEYVS---L---RE-VK--
	Serratia odorifera	270264807	-------MKPF-FW-AQR		-V-TPYVS---L---REIVT--
	Shewanella denitrificans	91792924	----A---SPI--AIA-R		MMSISHYS---L---TE-V---
	Shigella dysenteriae	82775572	-------MKPF-FW-A-L		-V-T-YVS---L---RE-VK--
	Vibrio cholerae	153831006	---------AF-AF-A-R		-L-TPYVS-------EE-VK--

Figure S14

Partial sequence alignment of a conserved region of lipid-A-disaccharide synthase a 2 aa insert that is present in all Xanthomonadales. The CSI has also been found to be shared by *Cardiobacterium hominis, Allochromatium vinosum and Alteromonadales bacterium.*
