# Supplementary material for: Phylogenomics and Molecular Signatures for Species from the Plant Pathogen-Containing Order Xanthomonadales
Source: PLoS One. 2013 Feb 8;8(2):e55216. doi: 10.1371/journal.pone.0055216 (PMC3568101; doi:10.1371/journal.pone.0055216)
Supplement: Figure S15 — Partial sequence alignment of carbamoyl phosphate synthase large subunit a 1 aa insert that is present in Xanthomonadales. The CSI has also been found to be shared by Marinobacter sp. ELB17. (PDF) [file pone.0055216.s015.pdf]

			403		457
	Xanthomonas oryzae	166711938	ESLQKALRGLETGKIGLDPT	G	LDLSSEDDIAALKRELKAPGPERLFYVADAFRAG
	Xanthomonas fuscans	294666705	--------------------	-	----------T-----------------------
	Xanthomonas campestris	289668839	-------------------S	-	----------T-----------------------
	Xanthomonas axonopodis	161485715	--------------------	-	----------T-----------------------
	Xanthomonas albilineans	285018123	--------------------	-	---RN---L---RH--------------------
	Xanthomonas gardneri	325922458	--------------------	-	----------------------------------
	Xanthomonas perforans	325925176	--------------------	-	----------T-----------------------
Xanthomonadales	Xanthomonas vesicatoria	325915492	--------------------	-	---G--E-M-------------------------
	Pseudoxanthomonas spadix	357417417	--------------V-----	-	---G----M-S-R--------------G------
	Pseudoxanthomonas suwonensis	319787363	--V-----------V-F---	-	---AD-E-L-T-R---------------------
	Rhodanobacter sp. 2APBS1	352082139	-----------I--T--N--	-	---GTD-GL-V-----RE-R-D-V-HL-------
	Stenotrophomonas sp. SKA14	254524347	--V-----------V-F---	-	---AN-E-LQT-R------------F--------
	Stenotrophomonas maltophilia	194365589	--M-----------V-F---	-	---TN---LQT-R------------F--------
	Xylella fastidiosa	15837709	------V-------V--E--	-	--------LVV---------A-------------
→	Marinobacter sp. ELB17	126664879	-----------V-SD-F-EM	L	SEFD--ESRQT-T---NV--A--IW-IG------
	Aeromonas hydrophila	117618944	---H----------T-F--M		V--NAP-SLTRIRH--QDA-A--I--I-------
	Aeromonas salmonicida	145298639	---H----S-----T-F--M		V--NAP-SLTRIRH--QDA-CD-IW-I-------
	Arsenophonus nasoniae	284006380	--M--------VAAT-F--K		V--DEF-SLTKIR----DA-A--IW-I-------
	Azotobacter vinelandii	226946317	--V--------V-VS-F--K		--PGNPEAGSI-----TV--A--IW---------
	Candidatus Hamiltonella	238897353	-----------V-AT----K		VS-NDPEA-TSIR----DA-G--IW-I-------
	Candidatus Regiella	304414251	-----------V-AT----K		VN-DDPQALSKIR----EA-A--IW-I-------
	Cellvibrio japonicus	192362421	-----------V-SA-FESK		V-YTT-EGA-KVR---TTA-A--IW--G----M-
	Citrobacter youngae	283835095	-----------V-AT-F--K		VS-DDPEALTKIR----DA-A--IW-I-------
	Cronobacter sakazakii	156935438	--M--------V-AT-F--K		VS-DDPEALTKIR----DA-A--IW-I-------
	Dickeya dadantii	307132640	-----------V-AT-F--K		V--DDPEALTKIR----DA-GD-IW-L-------
	Edwardsiella ictaluri	238918633	-----------V-AS-F--K		VN-DDPQALTKIR----DA-A--IW-I-------
	Enterobacter cancerogenus	261338962	-----------V-AT-F--K		VS-DDPEALTKIR----DA-A--IW-I-------
	Erwinia tasmaniensis	188532866	--M--------V-AN-F--K		V--NDPEALTTIR----DA-SD-IW-I-------
	Escherichia coli	110640245	-----------V-AT-F--K		VS-DDPEALTKIR----DA-A--IW-I-------
	Idiomarina loihiensis	56460088	--M--------L-AS-F--I		V--EAK-ARSKVI----E--A--IW-I-----L-
	Klebsiella pneumoniae	206579070	-----------V-AT-F--K		VS-DDPEALTKIR----DA-A--IW-I-------
	Marinobacter algicola	149375766	------M----V-SD-FEEK		V--NTT-GQET-TQ--NV--A---W-IG------
	Marinomonas sp. MWYL1	152998030	-----------V-SD-FN-Q		--FAE-NSKEK-AY--QS--SD-IW-IG----S-
Other Bacteria	Moritella sp. PE36	149908713	--M---I----I--N-F--E		VN-ND-SAA-KIRQ--TEA-A--I--I---Y-I-
	Nitrosococcus oceani	77166030	--------S----TD-FNEK		V--AA--VVET-RYQ-RV-SAD-IY-L-------
	Oceanospirillum sp. MED92	89095225	-----------V-AT-F--I		I-IT---ARSTIV--M-E--A--IW-LG----M-
	Pantoea ananatis	291616228	--V--------V-AN-F--K		VH-DDAEALTRIR----DA-SD-IW-I-------
	Pectobacterium carotovorum	227327742	-----------V-AT-F--K		V--DDPEALTKIR----DA-A--IW-I-------
	Photorhabdus asymbiotica	253988043	-----------V-AT-F--K		V--DDPEALTKIR----DA-G--IW-I-------
	Proteus mirabilis	227358194	--M--------V-AT-F--K		VN-DDPEALTKIR----EA-SD-IW-I-------
	Providencia stuartii	183601001	--M--------F-AT-F--K		VSQDDPEALTRIR----DA-A--IW-I-------
	Pseudomonas aeruginosa	15599950	--V--------V-AT-F--K		---NDPEADSI-----TV-SA--VW---------
	Saccharophagus degradans	90022373	-----------V-SA-FE-R		V--TGDNALELIR-D-ST--A--IW---------
	Salmonella enterica	161504813	-----------V-AT-F--K		VS-DDPEALTKIR----DA-A--IW-I-------
	Serratia odorifera	270263912	-----------V-AT-F--K		VN-DDPEALTKIR----DA-CD-IW-I-------
	Serratia proteamaculans	157368959	-----------V-AT-F--K		VS-DDPEALTKIR----DA-SD-IW-I-------
	Shewanella amazonensis	119775635	--V--------VSRN-F--I		V---DA-ALQKIRH---E--AD-IW-IG------
	Shigella sonnei	74310651	-----------V-AT-F--K		VS-DDPEALTKIR----DA-A--IW-I-------
	Sodalis glossinidius	85058398	-----------V-AS-F--K		VN-DDPEALTT-R----DA-G--IW---------
	Teredinibacter turnerae	254787220	-----------V-SA-FE-R		---T-D-A-DRIRHD-AT--A--IW-IG------
	Vibrio shilonii	149190314	-----------V-AT-F-EM		V--DAP-ALSKIRH---EA-A--IW-I-------
	Xenorhabdus bovienii	290474746	--M--------V-VT-F--K		VN-DDPQSLTKIRS---SA-A--IW---------
	Yersinia enterocolitica	123440986	-----------V-AT-F--K		VS-DDPEALTKIR----EA-SD-IW-I-------

Figure S15

Partial sequence alignment of a conserved region of carbamoyl phosphate synthase large subunit a 1 aa insert that is present in all Xanthomonadales. The CSI has also been found to be shared by *Marinobacter* sp. ELB17.
