# Supplementary material for: Phylogenomics and Molecular Signatures for Species from the Plant Pathogen-Containing Order Xanthomonadales
Source: PLoS One. 2013 Feb 8;8(2):e55216. doi: 10.1371/journal.pone.0055216 (PMC3568101; doi:10.1371/journal.pone.0055216)
Supplement: Figure S16 — Partial sequence alignment of putative secreted protein showing a 1 aa insert that is present in Xanthomonadales. The CSI has also been found to be shared by Teredinibacter turnerae. (PDF) [file pone.0055216.s016.pdf]

Group	Species	ID	1285		1318
Xanthomonadales	Xanthomonas campestris	188992701	VLARVNRAPLGTLRALYD	N	QRDKALTGLSLVHLG
	Xanthomonas fuscans	294627964	------------------	-	---------------
	Xanthomonas axonopodis	21241899	------------------	-	---------------
	Xylella fastidiosa	15837853	--------------T---	-	E-G--VS--------
	Xanthomonas perforans	325925285	------------------	-	---------------
	Xanthomonas vesicatoria	325915165	------------------	-	--A------------
	Pseudoxanthomonas spadix	357417253	-----G------------	-	D-V-SM---P-----
	Pseudoxanthomonas suwonensis	319786934	------------------	Q	EKGRS----P--R--
	Rhodanobacter sp. 2APBS1	352080521	---------------IF-	-	E-Q-LVAP-P-----
	Stenotrophomonas sp. SKA14	254522704	--------------T---	-	E-S--VG--------
	Stenotrophomonas maltophilia	194366795	--------------T---	-	E-S--VG--------
→	Teredinibacter turnerae	254784961	--SGLK-----V--N--R	R	DFNA-QS-YAQL---
Other Bacteria	Citrobacter koseri	157144543	----QQK----A--EIWE		R-SQ-AS--P-LQ--
	Cronobacter sakazakii	156932934	----QQK----A--D-WE		H-AQ-GS--P--Q--
	Cronobacter turicensis	260598906	----QQK----A--D-WE		H-AQ-GG--P--Q--
	Enterobacter cancerogenus	261340828	----QQK----A--EIW-		RHSQ-AS--P-MQ--
	Erwinia billingiae	300717957	----QQK----A----F-		KQGQ-K------Q--
	Escherichia coli	300920676	----QQK----A--EIWE		H-AD-AS--P-LQ--
	Klebsiella pneumoniae	206580715	----QQK----A--EIWS		RH-Q-RS--P-LQ--
	Pantoea ananatis	291618417	----QQ-----A-----E		K----KS--A-MQ--
	Photorhabdus asymbiotica	253988861	---SQQK----A--QV-E		R-NQ-GN-----Q--
	Photorhabdus luminescens	37525326	---NQQK----A--QV-E		R-QQ-GN-----Q--
	Proteus mirabilis	227356217	---NQQK----E--R--L		E-NQ-GS--A--Q--
	Providencia alcalifaciens	212710621	---TQQK----A--QI-G		-ANN-TS--A--Q--
	Pseudomonas entomophila	104783782	----SQQ----A--S---		R-AD-RS--P--Q-A
	Salmonella enterica	16765852	----QQK-------EIWE		R-SQ-AS--P-MQ--
	Serratia odorifera	270264736	----QQQ----A--Q--E		R-AD-RS--P--Q--
	Shigella boydii	187733068	----QQK----A--EIWE		H-AD-AS--P-LQ--
	Xenorhabdus bovienii	290476010	---SQQK---SG--Q--E		RHQQ-GN-----Q--
	Xenorhabdus nematophila	300724160	---GQQK---SG--Q--E		RHTQ-ES-----Q--
	Yersinia aldovae	238758827	---QQQQ-S--A--Q--T		RSNE-RS--P--Q--

Figure S16

Partial sequence alignment of a conserved region of putative secreted protein showing a 1 aa insert that is present in all Xanthomonadales. The CSI has also been found to be shared by *Teredinibacter turnerae*.
