# Supplementary material for: Phylogenomics and Molecular Signatures for Species from the Plant Pathogen-Containing Order Xanthomonadales
Source: PLoS One. 2013 Feb 8;8(2):e55216. doi: 10.1371/journal.pone.0055216 (PMC3568101; doi:10.1371/journal.pone.0055216)
Supplement: Figure S17 — Partial sequence alignment of a conserved region of aminopeptidase P, showing a 1 aa deletion that is commonly shared by Xanthomonadales. The CSI has also been found to be shared by Thioalkalivibrio sp. HL-EbGR7 and Alkalilimnicola ehrlichii. (PDF) [file pone.0055216.s017.pdf]

Group	Species	GI	211		246
Xanthomonadales	Xanthomonas fuscans	294627124	EYELQAEVEREFRAADAW		PAYGSIVGTGSNACVLHY
	Xanthomonas axonopodis	21244130	------------------		------------------
	Xanthomonas campestris	289662144	------------------		------------------
	Xanthomonas oryzae	166713279	------------------		--------S---------
	Xanthomonas albilineans	285017584	--Q----I-HA---G--C		--------S---------
	Xanthomonas vesicatoria	325918107	--A---------------		------------------
	Xanthomonas gardneri	325922293	---F--------------		------------------
	Pseudoxanthomonas suwonensis	319788224	----------V-------		---A----A-G-G-----
	Pseudoxanthomonas spadix	357418373	--Q---LM-WV--SQ---		--------A-A--VT---
	Stenotrophomonas maltophilia	194367049	-------L--V---S--V		---C----A-R-G-I---
	Stenotrophomonas sp. SKA14	254521029	-------L--V---N--V		---C----A-R-G-I---
	Xylella fastidiosa	71275757	--V----L--V-----SC		---T---AA-A-------
	Rhodanobacter sp. 2APBS1	352089789	---VE--LL-VV-GQG-V		--FPP--AG-A----M--
	Thioalkalivibrio sp. HL-EbGR7	220935926	---IE--LLY---R-GTE		---P----G-A-G-I---
	Alkalilimnicola ehrlichii	114321689	----E--FLAA--R-GGE		---P----G-G-G-----
Other γ-Proteobacteria	Alteromonas macleodii	239995906	--Q-E--IHH--AM-G-R	S	---ST---S-D---I---
	Azotobacter vinelandii	226946740	--H-E--L-Y---KGG-R	M	-------AG-R---I---
	Candidatus Regiella	304414301	--Q-EG-IQH--IQNG-R	F	---NT---S-A-G-I---
	Citrobacter koseri	157148448	--Q-EG-IHH--NRHG-R	Y	-S-NT---S-E-G-I---
	Congregibacter litoralis	88706374	-F--E--LLH--ARHG-R	H	A--P---AG-----TM--
	Dickeya zeae	251791161	--Q-EG-IHH--NRHG-R	Y	-S-NT---S-E---I---
	Enterobacter cloacae	295097444	--Q-EG-IHH--NRHG-R	F	-S-NT---G-E-G-I---
	Erwinia pyrifoliae	259909571	--Q-EG-IQH--NRHG-R	F	-S-NT---A-E---I---
	Escherichia coli	110643056	--H-EG-IHH--NRHG-R	Y	-S-NT---S-E-G-I---
	Klebsiella pneumoniae	206579369	--Q-EG-ILH--NRHG-R	F	-S-NT---G-E-G-I---
	Pantoea ananatis	291618745	--H-EG-IHH--NRHG-R	F	-S-NT---S-E-G-I---
	Pectobacterium wasabiae	261819870	--Q-EG-IHH--TRHG-R	Y	-S-NT---S-D---I---
	Photorhabdus asymbiotica	253988637	--Q-E--IHH--THQG-R	Y	---NT-I-S-E-S-I---
	Photorhabdus luminescens	37527469	--Q-E--IHH--THQG-R	Y	---NT---S-E-S-I---
	Proteus mirabilis	197285875	--Q--G-I-H--ISHG-R	F	-S-N----S-E-G-I---
	Providencia stuartii	183599852	--Q-CG-L-H--TRHG-R	Y	-S-N----S-E---I---
	Pseudomonas aeruginosa	152986578	--H-E--L-Y---KGG-K	M	-------AA-R---I---
	Psychromonas ingrahamii	119944379	--Q-E--IRY--AQQG-T	N	V--N---AG-HH--I---
	Salmonella enterica	16761839	--Q-EG-IHH--NRHG-R	Y	-S-NT---S-E-G-I---
	Serratia odorifera	270264936	--Q-E--ILH--TRLG-R	Y	-S-NT---G-E---I---
	Shigella sonnei	74313466	--H-EG-IHH--NRHG-R	Y	-S-NT---S-E-G-I---
	Sodalis glossinidius	85059983	--Q-EG-IQH--NRHG-R	F	-S-ST-A-S-E-G-I---
	Xenorhabdus bovienii	290476416	--Q-E--IHH--TRQG-R	Y	---NT---A-E---I---
	Yersinia pestis	22127173	--Q-EG-ILH--TRHG-R	Y	---NT---G-E-G-I---
β-Proteobacteria	Oxalobacteraceae bacterium	329903498	--QID--LLH---NHGSD	F	---T---A--A-------
	Methyloversatilis universalis	334133010	---IE--ILH---R-GSE	A	-------AG-A-------
	Limnobacter sp. MED105	149927953	-F-VE--LLYQ--KNGSE	A	-------AS-A-------
	Herbaspirillum seropedicae	300310037	--H-E--LLH---RNGSQ	Y	-------A--A-S-----
	Burkholderia glumae	238026252	---IE--LLH---RHG-A	G	-------AA-A-------
	Burkholderia gladioli	330815566	---IE--LLH---RHGSA	G	-------AA-A-------
	Chromobacterium violaceum	34498577	--Q-E--LLHV-VGHG-R	Q	---E---AA-A---T---
α-Proteobacteria	Sphingobium chlorophenolicum	334345114	-RQ-K-IL-DG---GGGE	G	L--D---A--R--AS---
	Sphingobium japonicum	294011039	-RQ-K-IL-DG---GGGE	G	L--D---A--R--AS---
	Novosphingobium aromaticivoran	87198178	-RQ-T-VL-DG--QGGGE	G	LS-D---A--R--AS---

Figure S17

Partial sequence alignment of a conserved region of aminopeptidase P showing a 1 aa deletion that is present in all Xanthomonadales. The CSI has also been found to be shared by *Thioalkalivibrio* sp. HL-EbGR7 and *Alkalilimnicola ehrlichii*.
