# Supplementary material for: Phylogenomics and Molecular Signatures for Species from the Plant Pathogen-Containing Order Xanthomonadales
Source: PLoS One. 2013 Feb 8;8(2):e55216. doi: 10.1371/journal.pone.0055216 (PMC3568101; doi:10.1371/journal.pone.0055216)
Supplement: Figure S18 — Partial sequence alignment of protoheme IX farnesyltransferase showing a 4 aa insert that is uniquely shared by subclade of Xanthomonadales after the divergence of Rhodanobacter sp. 2APBS1. (PDF) [file pone.0055216.s018.pdf]

			150		192
Xanthomonadales	Xylella fastidiosa	15837961	GAMPPMLGWAAVTGLSTAADW	INAS	LLVAIIFVWTPPHFWALA
	Xanthomonas campestris	21233252	--T------------P-S---	----	---L---I----------
	Xanthomonas oryzae	84625568	--T------------P-S---	----	---L---I----------
	Xanthomonas fuscans	294666751	--T------------P-S---	----	---L---I----------
	Xanthomonas axonopodis	21244603	--T------------P-S---	----	---L---I----------
	Xanthomonas vesicatoria	325918187	--T------------P-T---	----	I--L---I----------
	Xanthomonas perforans	325928849	--T------------P-S---	----	---L---I----------
	Xanthomonas albilineans	285017221	--A--L-----I--MQGQW--	AY--	---L--------------
	Pseudoxanthomonas suwonensis	319785801	--A--L-------NMQGSW--	QH-L	---L--------------
	Pseudoxanthomonas spadix	357418726	--T--L--------MQGPW--	GHSL	---L--------------
	Stenotrophomonas maltophilia	194364097	--------------MQGSS--	AYS-	---L---I----------
	Stenotrophomonas sp. SKA14	254525124	--------------MQGSS--	AYS-	---L---I----------
	Rhodanobacter sp. 2APBS1	352086526	--I--V---T----ALHPYAL		Q-CL--------------
Other γ-Proteobacteria	Alcanivorax borkumensis	110834767	--I--L---T----EAHPYA-		---L--------------
	Alkalilimnicola ehrlichii	114319463	--A--L---T----SVD-HAL		--FL-V------------
	Alteromonadales bacterium	119471373	-----L---VSE-NQMA--P-		---M---T----------
	Alteromonas macleodii	239993291	--A--L---T----EIH-HAL		---L---T----------
	Cellvibrio japonicus	192359423	-----L---T-I--EIHSHAL		---M---T----------
	Colwellia psychrerythraea	71278210	--I--L---T-M-NEVVPNAL		---L---T----------
	Congregibacter litoralis	88705362	--A--L---T----EVHGHAL		---L---A----------
	Grimontia hollisae	262273569	-----L---V----TLDSHAL		---ML--T----------
	Hahella chejuensis	83642966	--A--L---TS---TVDPHAL		---L---A----------
	Idiomarina loihiensis	56459364	--A--L---TS--NEIN-PAV		---M---T----------
	Kangiella koreensis	256823546	--I--L---TS---SADPHA-		---L---T---------S
	Legionella drancourtii	254495813	--A--L---T---NHLDPQAL		---L---T----------
	Legionella pneumophila	54293406	--A--L---T---DQLDPQAL		---L---T----------
	Marinobacter algicola	149375586	-----L---T----QVEGHAL		---L---A----------
	Marinomonas sp. MED121	87121367	-----L---VSI--TLEPNSL		---L---A----------
	Methylophaga thiooxidans	254491347	-----L---T----QVDPNAL		---L---A----------
	Nitrococcus mobilis	88811917	--A------T----SIDPNSL		--FL--------------
	Oceanospirillum sp. MED92	89093448	--A--L---T----TIEPNAL		---L---A---------C
	Psychromonas ingrahamii	119946339	-----L---ISE--QLA-QP-		I--M---T----------
	Reinekea sp. MED297	88798510	-----L---T----SI-GLPL		---L---A----------
	Rickettsiella grylli	160871949	--T--L---T----NIDSEGL		---L---I-------S--
	Saccharophagus degradans	90019693	--A--L---V----EVHGHAL		---L---A----------
	Shewanella baltica	126172401	-----L---T---NEFHGHAL		---I---T----------
	Teredinibacter turnerae	254784350	--A--L---T----ELDGHGL		---L---A----------
	Vibrio alginolyticus	91227004	-----L---T----QLH-NA-		---M---I----------
α-Proteobacteria	Phenylobacterium zucineum	197104010	--L--VI----A--SAPLNA-		--C----M---------S
	Caulobacter segnis	295687730	--L--AI----A--HAPLNA-		-M-----F---------S
	Parvularcula bermudensis	304320207	--L--VV----I--DAPL-A-		V-FS--------------
	Rhodospirillum centenum	209966878	--F---I-------DV-L-SV		I-F----F---------S
	Roseomonas cervicalis	296536351	--F---I-------HVSVEAV		VMF----F---------S
	Anaplasma phagocytophilum	88607088	--F--VI--TS---IPSIESL		--FM---L---------S
	Caulobacter sp. K31	167648459	--L--AI----A--HAPLNA-		-M-L---L---------S
	Oceanicaulis alexandrii	83945306	--F---I----A--DISLNSV		--F----L-----S----
	Fulvimarina pelagi	114708030	--F---I-------DVSIEGF		V-FL---L----------
	Roseobacter sp. AzwK-3b	149915656	--F---I----A--TVSIESV		-MF-L--M----------
	Beijerinckia indica	182677721	--F---V-Y--A--HFSLSSF		I-F----I----------
	Stappia aggregata	118590728	--F---I-------SVSLESF		V-FL---M----------
β-Proteobacteria	Nitrosospira multiformis	82701325	-----V--------EVS-DAL		--FL---A----------
	Thauera sp. MZ1T	217969470	--L--V---T-M--TVG-EA-		--MM-V-T----------
	Polynucleobacter necessarius	145590110	-----A-------NGLS-EA-		---L--------------
	Burkholderia ambifaria	115352945	-----A--------AVPGDA-		I--L-----------V--
	Nitrosomonas europaea	30249013	-----V--------EIS-DAL		--FL---A----------
	Aromatoleum aromaticum	56477833	-----V--------EVS-DAL		--FL---A----------
	Ralstonia eutropha	113866382	-----A------A-EVP-EA-		F--L---T----------
	Thiomonas intermedia	296137015	-----V------S-NVSVDAM		--FL---L----------

Figure S18

Partial sequence alignment of protoheme IX farnesyltransferase showing a 4 aa insert in conserved region that is uniquely shared by subclade of Xanthomonadales after the divergence of *Rhodanobacter* sp. 2APBS1.
