# Supplementary material for: Phylogenomics and Molecular Signatures for Species from the Plant Pathogen-Containing Order Xanthomonadales
Source: PLoS One. 2013 Feb 8;8(2):e55216. doi: 10.1371/journal.pone.0055216 (PMC3568101; doi:10.1371/journal.pone.0055216)
Supplement: Figure S19 — Partial sequence alignment of DNA polymerase III subunit alpha, showing a 1 aa insert that is uniquely present in Xanthomonadales except Rhodanobacter sp. 2APBS1. (PDF) [file pone.0055216.s019.pdf]

			583		638
Xanthomonadales	Xanthomonas albilineans	285018805	LTIIDWAVKAINVRHARAGIPAVEITAIPLDDVPT	Y	KDIFASGSTGAVFQFESSGM
	Xanthomonas axonopodis	21242159	--------------------DP-D-----------	-	-GV----N------------
	Xanthomonas fuscans	294666388	--------------------DP-D-----------	-	-GV----N------------
	Xanthomonas campestris	78047019	--------------------DP-D-----------	-	-GV----N------------
	Xanthomonas oryzae	166712747	--------------------DP-D--T--------	-	-GV----N------------
	Xanthomonas gardneri	325923963	--------------------DP-D--TL-------	-	-GV----N------------
	Xanthomonas vesicatoria	325916626	--------------------DP-D--T--------	-	-GV----N------------
	Xanthomonas perforans	325929585	--------------------DP-D-----------	-	-GV----N------------
	Xylella fastidiosa	71898629	------------K-----C-DP-D---L----I--	-	--V----N-S----------
	Stenotrophomonas maltophilia	194365029	------------K--E-----P-D-A------T--	-	-----N-N------------
	Stenotrophomonas sp. SKA14	254524078	------------K--E-----P-D-A------T--	-	-----N-N------------
	Pseudoxanthomonas suwonensis	319787804	------------R--E-----PLD-A-L----AA-	-	-------N------------
	Rhodanobacter sp. 2APBS1	352086322	------------A-R-K--EALL--S-L----PAS		YELLKKAQ-V----L-----
Other Bacteria	Alcanivorax borkumensis	110834019	----------A--KR--E-LDDLV-DH-----S-S		F-LLKR-D-T----L--Q--
	Alcanivorax sp. DG881	254428613	----------A--KRG-E-LDDLV-DH-----G-S		F-LLKR-D-T----L--Q--
	Alkalilimnicola ehrlichii	114321000	------T---V-ALREQR-ETPLD-AR-----RA-		F-LLKRCQ-T----L--R--
	Alteromonadales bacterium	119471146	---LQ---DMT---MQ-E-KDP-D-NT-----KKS		IELLLRAE-T----L--R--
	Cardiobacterium hominis	258546181	--V----LGH--A-RT-E-Q-PLD-ATL----PQ-		YALLQ-TR-T----L-----
	Chromohalobacter salexigens	92112711	-------LEMVDTVR--E-QGPLD-AS--------		F-MLKKAE-T----L--R--
	Citrobacter koseri	157147384	----N--LEM--A-REKN-E-PLD-A------KKS		F-MLQRSE-T----L--R--
	Cronobacter turicensis	260596604	----N--LDM--A-R-KQ-LEPID-A------KKS		F-MLQRSE-T----L--R--
	Dickeya zeae Ech1591	251790730	----N--LEM--A-REKQ-LEP-D-A------KNS		FGMLQRSE-T----L--R--
	Edwardsiella ictaluri	238918790	----N--LEM--A-R-CQ-LEP-D-A------QKS		F--LQRAE-T----L--R--
	Enterobacter cancerogenus	261338818	-------L-M--P-REKQ-LEPID-A------KKS		F-MLQRSE-T----L--R--
	Erwinia amylovora	292489212	-------LAM--AKR-KS-EAPID-A----E-KKS		F-MLQRSE-T----L--R--
	Escherichia coli	19548812	----N--LEM--K-R-KN-E-PLD-A------KKS		F-MLQRSE-T----L--R--
	Haemophilus influenzae	301169460	----K--LDM----MV-E-K-R-D-A------PES		FELLKRSE-T----L--R--
	Hahella chejuensis	83644688	---V----DI--RQ--EK-Q-PLD-NF-----A--		FNLLKH-E-T----L--R--
	Klebsiella pneumoniae	206579408	----N--LEM--K-R-KN-EGPLD-A------KKS		F-MLQRSE-T----L--R--
	Legionella longbeachae	270156895	-------LATV-RQRG-E-L-PID-SQ--T--PAS		F-LLKACQ-T----L--R--
	Marinobacter algicola	149377463	-------L-M--P-LEKQ-K--LD-NE-------S		F-MLKKAE-T----L--R--
	Methylococcus capsulatus	53804349	-------LAD--AKRRA--EEP-D-NR-----PK-		YELLGRCA-T----L--R--
	Pantoea ananatis LMG	291616361	-------L-M--P-REKQ-L-PID-AT---E-KKS		F-MLQRAE-T----L--R--
	Pasteurella multocida	15601899	----K--LDM--T-L-KE-K-P-D-AT-----PAS		FELLKRSE-T----L--R--
	Pectobacterium atrosepticum	50119986	-------LEM--A-R-KL-QEPID-AT---G-KKS		F-MLQRSE-T----L--R--
	Photorhabdus asymbiotica	253988140	----N--LEM--A-R-KK-LEPID-A----N-NKS		F-MLQRAE-T----L--R--
	Proteus mirabilis	227357235	----N--LEM--A-R-KKSLEP-D-S--S-T-QRS		F-MLQRSE-T----L--R--
	Providencia stuartii	188025561	----N--LEM--A-R-KKNLEP-D-A------QKS		F-MLQRSE-T----L--R--
	Pseudomonas aeruginosa	152987648	----K--MEI--REQ-KK-LEP-N-DF-----K--		YALLQKAE-T----L--R--
	Salmonella enterica	213609772	----N--LEM--K-R-KN-E-PLD-A------KKS		F-MLQRSE-T----L--R--
	Serratia odorifera	270264805	-------LGM--A-R-KS-LEPID-A----E-KKS		F-MLQRSE-T----L--R--
	Shewanella amazonensis	119774291	-------LGM--P-L-KL-K-P-D-A------SKS		FSVLKRYE-T----L--R--
	Shigella flexneri	41817835	----N--LEM--K-R-KN-E-PLD-A------KKS		F-MLQRSE-T----L--R--
	Teredinibacter turnerae	254785234	--------TM-DQQRT-L-E-PLD-S------PT-		F-MLKKAE-T----L--R--
	Tolumonas auensis	237808841	----K--LDM--P-LKTE-K-P-D-A------PTS		FK-LKNSE-T----L--R--
	Vibrio cholerae	940886	-------LGLV-P-LKK--K-P-R-E------ARS		FRNLQDAK-T----L--R--
	Xenorhabdus bovienii	290473672	----N--LDMV-A-RTHKNLEPID--------QIS		F-MLQRSE-T----L--R--
	Yersinia intermedia	238792741	----N--LEM--AKR-KT-LEPID-AS---E-KKS		F-MLQRSE-T----L--R--

Figure S19

Partial sequence alignment DNA polymerase III subunit alpha showing a 1 aa insert that is uniquely present in a subclade of Xanthomonadales except *Rhodanobacter* sp. 2APBS1.

Alpha and beta have 3 aa deletion at the same position
