# Supplementary material for: Phylogenomics and Molecular Signatures for Species from the Plant Pathogen-Containing Order Xanthomonadales
Source: PLoS One. 2013 Feb 8;8(2):e55216. doi: 10.1371/journal.pone.0055216 (PMC3568101; doi:10.1371/journal.pone.0055216)
Supplement: Figure S20 — Partial sequence alignment of a conserved region in the DEAD box helicase domain-containing protein showing a 1 aa insert that is specific for Xanthomonadales except Rhodanobacter sp. 2APBS1. (PDF) [file pone.0055216.s020.pdf]

Group	Species	ID	155		200
Xanthomonadales	Stenotrophomonas maltophilia	194364258	LDEADRMLDMGFLPSIKRILAKLPKQ	N	RQTLLFSATFEDNIRQLA
	Stenotrophomonas sp. SKA14	254524903	--------------------------	-	------------------
	Xanthomonas albilineans	285019480	--------------------T-----	-	-----------EG-K---
	Xanthomonas oryzae	166713488	--------------------T---R-	D	-----------E--K---
	Xanthomonas campestris	78049289	--------------------T---R-	D	-----------E--K---
	Xanthomonas axonopodis	21244335	--------------------T---R-	D	-----------E--K---
	Xanthomonas fuscans	294625538	--------------------T---R-	D	-----------E--K---
	Xanthomonas vesicatoria	325915753	--------------------T---R-	D	-----------E--K---
	Xanthomonas gardneri	325921307	--------------------T---R-	D	-----------E--K---
	Pseudoxanthomonas suwonensis	319788058	--------------------------	-	----------PES-K---
	Pseudoxanthomonas spadix	357418532	--------------------------	-	----------D---KL--
	Xylella fastidiosa	28198088	--------------------G---R-	-	----------AEP-K---
	Rhodanobacter sp. 2APBS1	352081056	--------------AL----QSV--K		----------APA-KT--
Other γ-Proteobacteria	Aeromonas hydrophila	117621566	------------IRD-R----L---K		--N-------A-E--E--
	Aliivibrio salmonicida	209808966	------------IHD--K--N----N		--N-------S-E-----
	Alkalilimnicola ehrlichii	114320766	------------IRD-R-VI-AV---		----M-----SKE-----
	Alteromonadales bacterium	119471733	------------IHD---LI--M-AK		--N-M-----S-D--A--
	Cellvibrio japonicus Ueda	192362464	------------IHD--KV--L----		--N-------S-D-KA--
	Citrobacter koseri	157146569	------------IHD-R-V-----PK		--N-------S-D-KA--
	Cronobacter sakazakii	156934713	------------IHD-R-V-----PK		--N-------S-D-KS--
	Dickeya zeae	251790108	------------IHD-R-V-----PK		--N-------S-E-KE--
	Enterobacter cloacae	296103277	------------IHD-R-V-----PR		--N-------S-D-KA--
	Erwinia amylovora	292487709	------------IHD-R-V-----AK		--N-------S-E-KT--
	Erwinia billingiae	300715940	------------IHD-R-V-----AK		--N-------S-E-KG--
	Hahella chejuensis	83645104	------------I-DV---VRAT-RE		----------SED-KN--
	Kangiella koreensis	256821317	-------------------IG----K		----------SES-KAIS
	Klebsiella pneumoniae	206579391	------------IHD-R-V-----AR		--N-------S-E-KG--
	Marinomonas sp. MWYL1	152995016	------------IHD--K---I---K		--N-------SPE-----
	Moritella sp. PE36	149908418	-------------RD-RK---V--QK		--N-------S-D-----
	Pantoea ananatis	291616785	------------IHD-R-V-----AK		--N-------S-E-KT--
	Pectobacterium wasabiae	261822185	------------IHD-R-V-----AK		--N-------S-E-KA--
	Providencia stuartii	183598226	------------IHD-R-VIS----K		--N-------SNE-K---
	Psychromonas ingrahamii	119946865	------------IHD------V----		--N-------SPD--R--
	Salmonella enterica	161504014	------------IHD-R-V-----PK		--N-------S-D-KA--
	Serratia proteamaculans	157369574	------------IHD-R-V-----AK		--N-------S-D-KG--
	Shewanella denitrificans	91794376	------------IRD-RKV--M----		--N-M-----S-D--E--
	Thiomicrospira crunogena	78485124	------------IHD-----KA---N		--N-------SNE-----
	Vibrio alginolyticus	91224087	------------IRD-RK---L----		--N-------S-E--D--
	Yersinia pestis	16611843	------------IHD-R-V-----AK		--N-------S-E-KG--
α-Proteobacteria	Acetobacter pasteurianus	258542730	I-----------I-D-EK-VGL--PN		---------MAPT--K--
	Granulibacter bethesdensis	114327981	I-----------I-DVE--VSL--HN		----F----MAPE--R--
	Hoeflea phototrophica	163758551	-----Q---I--M-A------MT-AT		---------MPKE----S
	Rhodospirillum rubrum	83594050	I-----------I-DVE--V-L---I		----F----MDKE--R--
	Acetobacter tropicalis	339018830	I-----------I-D-EK-VGL--AN		----F----MAPA-----
	Magnetospirillum gryphiswalden	144901078	I-----------I-DVQ--VSM---M		----F----LGPE--K--
β-Proteobacteria	Thauera sp. MZ1T	217970063	------------I-D------L----		--S-------S-E-KR--
	Nitrosospira multiformis	82703770	--------------D------L--S-		--S-M-----S-E-KK--
	Methylotenera mobilis	253996648	------------M-DL-----L----		--N-M-----SNE-KK--
	Burkholderia oklahomensis	167568882	------------IHD---V------K		--N-------S-E-KS--
	Neisseria meningitidis	325198557	------------IDD-RK-MQM----		----------SAP--K--

Figure S20

Partial sequence alignment of a conserved region in the DEAD box helicase domain-containing protein showing a 1 aa insert that is specific for Xanthomonadales except *Rhodanobacter* sp. 2APBS1.
