# Supplementary material for: Phylogenomics and Molecular Signatures for Species from the Plant Pathogen-Containing Order Xanthomonadales
Source: PLoS One. 2013 Feb 8;8(2):e55216. doi: 10.1371/journal.pone.0055216 (PMC3568101; doi:10.1371/journal.pone.0055216)
Supplement: Figure S21 — Partial sequence alignment of a conserved region in ribose-5-phosphate isomerase A, showing a 1 aa insert that is commonly shared by Xanthomonadales except Rhodanobacter sp. 2APBS1. (PDF) [file pone.0055216.s021.pdf]

Group	Species	Accession	127		169
Xanthomonadales	Stenotrophomonas maltophilia	194367055	FPLPVEVIPMARSLIARQIRDM	T	GGQPTWREGVVTDNGNQILD
	Stenotrophomonas sp. SKA14	254522924	--------------V-------	-	--------------------
	Xanthomonas oryzae	84622680	--------------V----LAL	-	----V--D--------VV--
	Xanthomonas campestris	289668028	--------------V----LAL	-	----V--D--------VV--
	Xanthomonas axonopodis	21109766	--------------V----LAL	-	----V--D--------VV--
	Xanthomonas fuscans	294627128	--------------V----LAL	-	----V--D--------VV--
	Xanthomonas albilineans	285017577	----I---------V--E-LAR	S	----V--D--I-----V---
	Xanthomonas gardneri	325922299	--------------V----LAL	-	----V--DN-I-----VV--
	Xanthomonas perforans	325927881	--------------V----LAL	-	----V--D--------VV--
	Xanthomonas vesicatoria	325917033	--------------V----LA-	-	----V--D--------VV--
	Xylella fastidiosa	71897970	----I---------V----MTL	-	D---V-----I-----V---
	Pseudoxanthomonas spadix	357418380	--------------V--E-LTL	-	----V--D--T-----L---
	Pseudoxanthomonas suwonensis	319788230	--------------V--R-LAL	-	----V--D--------W---
	Rhodanobacter sp. 2APBS1	352089795	--V-I----------G-E-VKR		--H-V--D--------W-I-
Other γ-Proteobacteria	Aeromonas hydrophila	117620736	------------EYV--E--KL		--N-V-----------H---
	Alcanivorax borkumensis	110835467	----I--------YV--KLVAL		----QY---FL-----I---
	Alcanivorax sp. DG881	254429859	-------------YV--KLVAL		----EY---F------I---
	Aliivibrio salmonicida	209695960	-------------YV--ELVKL		--D-AY----------M---
	Azotobacter vinelandii	226946833	-------------HV---LVML		--D-VY----------V---
	Beggiatoa sp. PS	153877568	-------------YV--ELVK-		--K-V---AFT-----V---
	Candidatus Regiella	304414795	-------------F---ELIKL		--M-KY-QN-L-------I-
	Cellvibrio japonicus	192361815	-------------FV--ELVKL		--A-VY----I-----V---
	Citrobacter koseri	157148454	-------------AV---LVKL		--R-EY-Q--------V---
	Cronobacter turicensis	260599255	-------------AV---LVKL		--R-EY-Q--------V---
	Dickeya zeae	251788159	------------AYV--ELVKL		----VY-D--------I---
	Edwardsiella ictaluri	238921219	------------AYV--ELVKL		----VY-Q--L-----V---
	Enterobacter cloacae	296104576	-------------AV--ELVKL		--R-EY-Q--------V---
	Erwinia pyrifoliae	283479656	-------------YV--ELVKL		--L-EY-QD-------I---
	Escherichia coli	188494875	-------------AV---LVKL		--R-EY-Q--------V---
	Grimontia hollisae	262273693	-------------YV--ELVKL		--D-CY---CI-----V---
	Klebsiella pneumoniae	206578185	-------------AV---LVKL		--R-EY-Q--------V---
	Marinomonas sp. MED121	87123015	----------S--YV--ELVKL		--D-VY--------------
	Nitrosococcus watsoni	300113160	-------------YV----VKL		--E-VY---FI---------
	Pantoea ananatis	291618750	-------------YV--ELVKL		--L-EY-QN-------I---
	Pectobacterium wasabiae	261823110	-------------YV--ELAKL		----VY-Q--------V---
	Photobacterium profundum	90412060	-------------F-G-ELVKL		--D-EY-Q--------I-I-
	Photorhabdus asymbiotica	253988632	-------------YV--ELVKL		--V-KY-QN-------V---
	Proteus mirabilis	197285883	-------------YV--ELVKL		--L-EY--N-------V---
	Providencia stuartii	183599858	-------------YV--ELVKL		--V-EY-QN-------V---
	Pseudomonas putida	167036190	-------------HV---LVKL		--D-VY----------V---
	Salmonella enterica	161506390	-------------AV---LVKL		--R-EY-Q--------V---
	Shewanella violacea	294139516	-------------YV---LVKL		--D-VY---CL-----I---
	Shigella dysenteriae	194431760	-------------AV---LVKL		--R-EY-Q--------V---
	Sodalis glossinidius	85059988	------------AWV--ELVRL		--A-VY-Q--------I---
	Tolumonas auensis	237808778	---------L--EYV--EL-KL		--N-VY----I---QC----
	Vibrio cholerae	15642476	-------------YV--ELVKL		--D-VY----I-----V---
	Xenorhabdus nematophila	300722096	------------AYV--ELVKL		--T-VY--N-------D---
β-Proteobacteria	Nitrosomonas eutropha	114331805	-------------YV--E-ALL		----A--QDFT-----V---
	Sideroxydans lithotrophicus	291615188	----I--------Y---ELVKL		----KL---FT-----V---
	Thiobacillus denitrificans	74318382	-------------HV--ELVKL		----RL---FT-----V---
	Laribacter hongkongensis	226939813	--------------V--KLVKL		--H-E---NF------L---
	Chromobacterium violaceum	34496715	----I--------YV--ELVKL		--H-EL-Q--T-----V---
	Gallionella capsiferriformans	302879703	----I--------SV---LVAL		----ML---FT-----I-I-
	Thiomonas intermedia	296136750	-------V----E-V--RL-AL		--D-RE-S-Y------L---
	Nitrosospira multiformis	82701570	------------GHV--E-VRL		----ML-Q-FT-----V---

Figure S21

Partial sequence alignment of a conserved region in ribose-5-phosphate isomerase A showing a 1 aa insert that is commonly shared by Xanthomonadales except *Rhodanobacter* sp. 2APBS1.

Alpha has longer insert about 8-10 aa long
