# Supplementary material for: Phylogenomics and Molecular Signatures for Species from the Plant Pathogen-Containing Order Xanthomonadales
Source: PLoS One. 2013 Feb 8;8(2):e55216. doi: 10.1371/journal.pone.0055216 (PMC3568101; doi:10.1371/journal.pone.0055216)
Supplement: Figure S22 — Partial sequence alignment of a conserved region in DNA polymerase I, showing a 1 aa insert that is uniquely shared by a subclade of Xanthomonadales except Rhodanobacter sp. 2APBS1. (PDF) [file pone.0055216.s022.pdf]

```
                                                    136                                              180
Xanthomonadales
Xanthomonas axonopodis         21244827     AQLVRPGIELVNTMSGSRMDSDA A VIAKFGVRPEQIVDLLALMGD
Xanthomonas fuscans            294664756    ----------------------- - -V-------------------
Xanthomonas oryzae             166710273    ----------------------- - ---------D-----------
Xanthomonas campestris         78049756     ----------------------- - ---------D-----------
Xanthomonas albilineans        285016940    -------VQ-------------- - -ME----L-H-----------
Xanthomonas vesicatoria        325917393    ----------------------- - ---------N-----------
Xanthomonas perforans          325924860    ----------------------- - ---------D-----------
Xanthomonas gardneri           325923784    ----------------------E - ---------D-----------
Stenotrophomonas maltophilia   190576375    --------------T-------- - -ME-----AD--I--------
Stenotrophomonas sp. SKA14     254524679    --------------T-------- - -MD-----AD--I--------
Xylella fastidiosa             71276059     -------V------T------AM - --N----A-D--I-F------
Pseudoxanthomonas spadix       357415770    -------V--------------- - -MD-------------S----
Rhodanobacter sp. 2APBS1       352086477    ----GSHVT------NTVL--AG   -ME----M-A--I-F---T--
Other γ-Proteobacteria
Azotobacter vinelandii         226942232    ----DGH-T-----T--VL-HAG   -AE----G--L-I-Y------
Candidatus Hamiltonella        238898050    ----S-H-T-I---NDKTL-P-G   -CE----P-DL----------
Citrobacter koseri             157147357    ----T-N-T-I---TNTILGP-E   -VN-Y--P--L-I-F------
Enterobacter cloacae           296105444    ----T---T-I---TNTILGPEE   -V--Y--P--L-I-F------
Escherichia coli               195940271    ----T-N-T-I---TNTILGPEE   -VN-Y--P--L-I-F------
Haemophilus influenzae         145640969    ----DDN-M-I---NN-LL-REG   --E-Y-IP--L-I-Y------
Haemophilus parainfluenzae     301154826    ----DDN-M-I---NNTLL-R--   --E-Y-IP--L-I-Y------
Haemophilus somnus             170717348    ----DDN-M-I---NN-LL-R-G   --D-Y-IP--L---Y------
Klebsiella pneumoniae          238892277    ----T---T-I---TNTILGP-E   -VT-Y--P--L-I-F------
Legionella drancourtii         254497331    ----NEHVT-I----NYT--VAG   -KE----E-A--I-Y-T-V--
Marinobacter algicola          149378378    ----SDHVT-I---TDT---R-G   -VE---IG-D----Y---V--
Methylophaga thiooxidans       254492177    ----NQHVT-----TETTL-P-G   -KE---LP--L-I-F------
Photobacterium damselae        269103720    ----DEN-T-I---TDVV--PAG   -VE---IG--L-I-Y------
Pseudomonas aeruginosa         152985854    ----DGH-T-----T---L-V-G   -KE----G--L-I-F------
Pseudomonas entomophila        104779389    ----DGHVT-----T--VL-VAG   -HE----G--H-I-F------
Rickettsiella grylli           160872753    ----CEE-T-----TNT-L-RQG   --D----A----T-Y-S-I--
Salmonella enterica            161505500    ----T-N-T-I---TNTILGP-E   -VN-Y--P--L-I-F------
Shigella flexneri              110807452    ----T-N-T-I---TNTILGPEE   -VN-Y--P--L-I-F------
Teredinibacter turnerae        254784333    ----NEH-T-----TDTV--IEG   -K----IP--L-I-Y------
Vibrio alginolyticus           91224944     ----DDN-T-I---TNVV--REG   -VE---IP--L-I-Y------
α-Proteobacteria
Rhodobacteraceae bacterium     254511729    M---GD-V-MLDA-KNK-I-R-G   -FE----G--RV--VQ--A--
Ruegeria sp. R11               254474520    M---GG-V-MLDA-KNK-I---G   -FE----G-DRV--VQ--A--
Azospirillum sp. B510          288961988    M-------GMFDP-KNKAIGP-E   -FE----P--KV--VQ--A--
Rhodomicrobium vannielii       312115891    M------VTMLD--KNKVIGEAE   --E----P-SKV--VQS-A--
Roseobacter litoralis          338759679    M---GD-V-MLDA-KNK-I-R-G   -FE----Y--RV--VQ--A--
Silicibacter sp. TrichCH4B     259417724    M---GD-V-MLDA-KNK-I---G   -RE----G-DRV--VQ--A--
Phaeobacter gallaeciensis      163742064    M---GG-V-MLDA-KNK-I-T-G   -FE----G-DRV--VQ--A--
Granulibacter bethesdensis     114328629    M--IQ--V-MQDPIKQKTIGPAE   -ME----T--KMI-VQ-----
Sagittula stellata             126732262    M---GD-V-MLDP-KNK-I-REG   -ME----G--RV--VQ--A--
β-Proteobacteria
Kingella kingae                333376435    ----S-R-T-----KNEML-H-G   -V-----K----R-F------
Thauera sp. MZ1T               237654465    T------VRW-----EEVL-EAG   -A-----P--R---Y---V--
Azoarcus sp. BH72              119899889    T------VRW-----EEVL-EAG   -T-----P--R---Y---V--
Neisseria bacilliformis        329119683    ----NERVT-------ETL-T-G   -K-------D--R-Y------
Simonsiella muelleri           294789122    ----NTQ-T-----KNETL--AG   -MQ----K----R-F------
Aromatoleum aromaticum         56477692     T------VRW-----DEVL-EAG   -A-----P-AL---Y---V--
Burkholderia rhizoxinica       312795716    ----NERVT-I---TNE-L-R-G   -------P--R---Y-T-I--
Neisseria mucosa               261364888    ----NERVT-------ETL-IEG   -K-------D--R-Y------
```

Figure S22

Partial sequence alignment of a conserved region in DNA polymerase I showing a 1 aa insert that is uniquely shared by a subclade of Xanthomonadales except *Rhodanobacter* sp. 2APBS1.
