# Supplementary material for: Phylogenomics and Molecular Signatures for Species from the Plant Pathogen-Containing Order Xanthomonadales
Source: PLoS One. 2013 Feb 8;8(2):e55216. doi: 10.1371/journal.pone.0055216 (PMC3568101; doi:10.1371/journal.pone.0055216)
Supplement: Figure S23 — Partial sequence alignment of aspartyl-tRNA synthetase, showing a 4 aa deletion that is commonly shared by all Xanthomonadales except Rhodanobacter sp. 2APBS1. (PDF) [file pone.0055216.s023.pdf]

Group	Species	Accession	343		391
Xanthomonadales	Stenotrophomonas maltophilia	194366904	YGAKGLAYIKIADNGE		VSSPIQKFFSEESFAALVAHVGAGNGDIVFFGA
	Stenotrophomonas sp. SKA14	254524468	----------------		-----------A---------------------
	Xanthomonas axonopodis	21243880	----------LSET--		-----A-----------LK--------------
	Xanthomonas fuscans	294665399	----------LSET--		I----A-----------LK--------------
	Xanthomonas oryzae	166712997	----------LSET--		-----A-----------LK----S---------
	Xanthomonas campestris	289661892	----------LSET--		-----A------A----IK--------------
	Xanthomonas albilineans	285019195	--------V----S--		I--------DGAA-D--L------R--------
	Xanthomonas vesicatoria	325918028	--S-------LS-T--		-----A-----DA----LK--------------
	Xanthomonas gardneri	325922682	----------LSET--		-----A------A----L------------L--
	Xanthomonas perforans	325929372	----------LSET--		-----A-----------LK--------------
	Pseudoxanthomonas suwonensis	319786198	F----------DEA-A		I----------DA----LK--------------
	Pseudoxanthomonas spadix	357416639	--S-------LSEA--		-A---A---AQ-RL---I---------------
	Xylella fastidiosa	71901605	---T----A-L-PT--		IT---A-----DA----LS-I--EK--------
	Rhodanobacter sp. 2APBS1	352080098	-------WL-VE-LAK	GRDG	IN--VA--LDDAALDGVLKAT--QS--------
Other γ-Proteobacteria	Actinobacillus minor	257465380	-------WA--N-VNA	GMEG	IQ--VA--LN--V-K--IERTS-Q----L----
	Actinobacillus succinogenes	152979462	-------WL-VN-VNA	GLEG	-Q---A--L---KIK-IFDRTS-QT---L----
	Aeromonas hydrophila	117618858	-------WM-VNEAAN	GIEG	-Q--VA--L-DAIVREIL-RT--AD---I----
	Aggregatibacter aphrophilus	251793073	-------WA-VN-INA	GLEG	-Q---A--LN-NVWK--AER-K-QT---L----
	Alteromonas macleodii	196156536	-------W--VN-A-A	GLEG	LQ---L--LT-DITN-ILERT--QS---LL---
	Arsenophonus nasoniae	284007618	-------WM-VNSLA-	NQAD	IQ---A--LPQHVIDN-L-RTQ-KA---LL---
	Citrobacter koseri	157145355	----------VTERAK	GLEG	IN--VA--LNA-IVE-ILDRTA-QD--MI----
	Congregibacter litoralis	88705602	---R---W--VN-LAA	GVEG	LQ---L--MP-DVV-SMMERL-VA---------
	Cronobacter sakazakii	156933556	----------VNERAK	GLDG	IN--VA--LNA-IVE-ILERT--QD--MI----
	Dickeya zeae	251789862	----------VNEKAK	GLEG	IQ--VA--LNDGIL-D-L-RTA-DD---L----
	Edwardsiella ictaluri	238919498	-------WM-VN-RAA	GMDG	-Q---A--LNA-VLEGIL-RS--QS---I----
	Enterobacter cloacae	296101795	----------VTERAK	GLEG	IT--VA--LNA-IVE-ILERT--QD--MI----
	Erwinia tasmaniensis	188533627	----------VNERAK	GIEG	IT--VA--LNA-IVESILQRTA-AD--LI----
	Escherichia	284921786	----------VNERAK	GLEG	IN--VA--LNA-IIE-ILDRTA-QD--MI----
	Grimontia hollisae	262276116	-------WV-VN-RAA	GMEG	IQ--VA--LT-DVVN-ILERT--ES---IL---
	Haemophilus ducreyi	33151787	-------WA--N-INA	GMDG	IQ--VA--LNA-I-N--IERTA-Q----L----
	Halorhodospira halophila	121999005	----------VN-PAQ	GVEG	LQ---V--LT--AVRGILERT--QA--V-----
	Klebsiella pneumoniae	206581095	----------VTERAK	GMDG	IN--VA--LTA-IVE-ILERT--QD--MI----
	Marinobacter algicola	149375108	----------VN-TSK	GVEG	LQ---I--LGDDVAQ-VIERS--ED--------
	Nitrosococcus watsoni	300113111	----------VIERSR	GREG	LQ---L--LPD-VIG-MLERI--E---------
	Pantoea ananatis	291617759	-------WM-VNERAK	GLEG	-Q--VA--LNADIVET-LSRTS-QD---I----
	Pectobacterium wasabiae	261821375	----------VNERAK	GLEG	-Q--VA--L--DVLS--LDRTA-QD---L----
	Photobacterium profundum	90411660	-------WM-VN-REA	GFAG	-Q--VA--LN--VV-T-LDRTQ-QT---IL---
	Photorhabdus asymbiotica	253989953	-------W--VNERAA	GLEG	-Q---A--LNA-VIDG-LSRT--QD---L----
	Proteus mirabilis	197284982	-------WM-VNEKAK	GIEG	-Q---A--LTNDVVNSIL-ATN-TD--LI----
	Providencia stuartii	188025998	-------WM-VNERAK	GLEG	IQ--VA--LN-DVINQ-LERTE-AD---LL---
	Pseudomonas syringae	289650939	----------VNERAK	GPEG	LQ---V--IP-DNLNVILDR---VD--------
	Salmonella enterica	161502985	----------VN-RAK	GLDG	IT--VA--L-T-IVE-IL-RT--Q---MI----
	Serratia proteamaculans	157371021	-------WL-VN-RAA	GMEG	-Q---A--L-A-VLE-IL-RTN-QS---L----
	Shewanella amazonensis	119774978	---R---WM-VNNLAA	GVEG	IQ--VA--LN-DIIKEII-RTK-AD---I----
	Shigella boydii	82543705	----------VNERAK	GLEG	IN--VA--LNA-IIE-ILDRTA-QD--MI----
	Vibrio cholerae	121730021	-------WL-VN-LAA	GMEG	IQ--VA--LT--IIQ-IIERTQ-QT---IL---
	Xenorhabdus bovienii	290475273	-------WM-VNNRAA	GLEG	-Q--VA--L-A-VVEG-LSRTN-QD---L----
	Yersinia pestis	22126144	-------WL-VN-RAA	GLEG	-Q---A--L-A-VLD-ILVATQ-ES---L----
β-Proteobacteria	Bordetella petrii	163859168	----------VN-ASK	GREG	LQ---V-NLHDAAL-E-LKRS--QS---I----
	Polaromonas naphthalenivorans	121603698	-------W--VN-VSK	GREG	LQ---V-NIHDAAV-EILKRT--Q----L----
	Achromobacter xylosoxidans	317401441	----------VNEVAK	GRDG	LQ---V-NLHDAAL-E--KRT--QD---I----
	Herminiimonas arsenicoxydans	134096004	----------VNEKAK	GRDG	LQ---V-NIHD-AL-KIIEAT--QD--LI----
	Achromobacter xylosoxidans	338782313	----------VNEVAK	GRDG	LQ---V-NLHDAAL-E--KRT--QD---I----
	Alicycliphilus denitrificans	330823547	-------W--VNEAAK	GRDG	LQ---V-NIHDAAI-EILKRT--QD--L-----

Figure S23

Partial sequence alignment of a conserved region in aspartyl-tRNA synthetase showing a 4 aa deletion that is commonly shared by all Xanthomonadales except *Rhodanobacter* sp. 2APBS1.
