# Supplementary material for: Phylogenomics and Molecular Signatures for Species from the Plant Pathogen-Containing Order Xanthomonadales
Source: PLoS One. 2013 Feb 8;8(2):e55216. doi: 10.1371/journal.pone.0055216 (PMC3568101; doi:10.1371/journal.pone.0055216)
Supplement: Figure S24 — Partial sequence alignment of a conserved region of 2-oxoglutarate-dehydrogenase E1 component, showing a 1 aa deletion that is unique to Xanthomonadales except Rhodanobacter sp. 2APBS1. (PDF) [file pone.0055216.s024.pdf]

Group	Organism	ID	782		830
Xanthomonadales	Stenotrophomonas maltophilia	194366403	PKSLLRHKLAVSTLDELANGEFQHLIGD		ANADAKKVKRVVLCSGKVYYD
	Stenotrophomonas sp. SKA14	254522086	----------------------------		---------------------
	Xylella fastidiosa	9106591	--------------------K---I-P-		DA--P-H---I-M-A------
	Xanthomonas campestris	188992180	--------------E---D-Q-----P-		-K---S---------------
	Xanthomonas fuscans	294625477	------------S-E---D-Q-----P-		-K---A---------------
	Xanthomonas axonopodis	77748592	------------S-E---D-Q-----P-		-K---A--R------------
	Xanthomonas oryzae	188577095	------------S-E---E-------P-		-K---A---------------
	Xanthomonas albilineans	285018444	------------S-K---D-------P-		SK-EP------IA--------
	Xanthomonas vesicatoria	325914050	------------S-E---D-------P-		-K---A---------------
	Xanthomonas gardneri	325919392	------------S-E---E-------P-		-K---G---------------
	Xanthomonas perforans	325925702	------------S-E---D-Q-----P-		-K---A---------------
	Pseudoxanthomonas spadix	357417263	------------S-E-M-E-Q-----P-		PK--P-------A-A------
	Pseudoxanthomonas suwonensis	319787382	--------------------------P-		-S-------------------
	Rhodanobacter sp. 2APBS1	352081385	--------------------S--LV--E	H	RALA-----------------
Other Bacteria	Acinetobacter haemolyticus	294651214	------------S-------S--TV-DE	V	DQINKAD-T-L---G------
	Aeromonas salmonicida	145299313	-------P----K-E---E-T--NA--E	I	DAL-P-G-----F--------
	Aggregatibacter aphrophilus	251792222	-------P----SME--V--T--TV--E	I	DEL-P-------M-A------
	Alcanivorax borkumensis	110834357	--------K-T-S-ED--H-T-HSVLD-	L	-DL-S--I----M--------
	Alteromonas macleodii	239993976	-------PM---S-E---E-K-HNV--E	I	DDI-P-N-----M--------
	Arsenophonus nasoniae	284007862	-------P----S-E--------QV-SE	V	DSI------------------
	Candidatus Regiella	304414842	-------P--T-S-------T--PA--E	I	DPLN---ID--LI--------
	Chromohalobacter salexigens	92113343	--------E-T---ED----R-EMVLP-	Q	GKR--ES--------------
	Citrobacter koseri	157146668	-------P----S-------A--PA--E	V	DEL-PQG-----M--------
	Cronobacter turicensis	260597124	-------P------------A--PA--E	V	DEL-PQG-------A------
	Dickeya dadantii	307130097	-------P--T---------Q--PA--E	I	DEL-P-A--------------
	Edwardsiella tarda	294637434	-------P--I-S-E---Q-S--PA--E	I	DPI-PQQ-----M--------
	Enhydrobacter aerosaccus	257453551	--------------E---Q-Q--TV-PE	I	DQL-NNQ-T-L---G------
	Enterobacter cancerogenus	261341312	-------P----S-------T-LPA--E	I	DEL-P-G-----M--------
	Erwinia tasmaniensis	188534431	-------P----S-E-----A--PA--E	I	DALE--S--------------
	Escherichia coli	43019	-------P----S-E-----T-LPA--E	I	DEL-P-G-----M--------
	Haemophilus influenzae	145633339	-------P----S----I--T--TV--E	I	DELAP-D-----M--------
	Idiomarina loihiensis	56460609	-------P----EM-D--DHG--NA--E	I	DDLNP-------F--------
	Klebsiella pneumoniae	206575964	-------P----SM------T-MPA--E	I	DEL-PQA--------------
	Mannheimia haemolytica	254361862	-------P----S----I--S--NV--E	L	D-I-P-Q-----M--------
	Moritella sp. PE36	149911921	-------P--I-S-S---D-T--NI--E	I	DTL-SAQ-----M------F-
	Nitrococcus mobilis	88811364	---------ST-------Q-T--LV-DE	I	DALS-GQMR-II-------F-
	Pantoea sp. aB	304395707	-------P--I---------S--PA--E	V	DDL-PQG--------------
	Pasteurella dagmatis	260914173	-------P----SM---I--K--NV-PE	V	DAL-P-Q-R---M--------
	Pectobacterium atrosepticum	50120300	-------P--I-S-------S--PA--E	V	EEL-PAA--------------
	Photobacterium damselae	269103075	-------P-CI--M------T--AA-DE	V	DAL-PTQ-----F--------
	Photorhabdus asymbiotica	253990448	-------P----S-------K--TV--E	A	DEL-P-D--------------
	Proteus mirabilis	197284466	-------P----S-----D-K-LPV--E	L	DELNPAD-----M--------
	Providencia alcalifaciens	212712445	-------P----SM------S--PV--E	I	DEINP-D-----M--------
	Pseudomonas aeruginosa	152984422	----------I---ED----S--TV-PE	I	DSL-P---D------------
	Serratia proteamaculans	157369510	-------P--T-S-------T-LPA--E	I	DDL-P-A--------------
	Shigella boydii	82543153	-------P----S-E-----T-LPA--E	I	DEL-P-G-----M--------
	Sodalis glossinidius	85058854	-------P--I-S------SM--PA--E	V	DDI-PQG-----M--------
	Vibrio fischeri	59711430	-------P-CT---E---E-N--SA-PE	I	DAL-P-Q-----F------F-
	Xenorhabdus bovienii	290474111	-------P----S-------K-EPV--E	I	DAL-P-G--------------
	Yersinia pestis	22126943	-------P--T-S-------S-LPA--E	I	DEL-P-GI----M--------

Figure S24

Partial sequence alignment of a conserved region of 2-oxoglutarate-dehydrogenase E1 component showing a 1 aa deletion that is commonly present in subclade of Xanthomonadales except *Rhodanobacter* sp. 2APBS1
