# Supplementary material for: Phylogenomics and Molecular Signatures for Species from the Plant Pathogen-Containing Order Xanthomonadales
Source: PLoS One. 2013 Feb 8;8(2):e55216. doi: 10.1371/journal.pone.0055216 (PMC3568101; doi:10.1371/journal.pone.0055216)
Supplement: Figure S25 — Partial sequence alignment of a conserved region of coproporphyrinogen III oxidase, showing a 1 aa deletion that is unique to Xanthomonadales except Rhodanobacter sp. 2APBS1. (PDF) [file pone.0055216.s025.pdf]

			166		215
Xanthomonadales	Stenotrophomonas maltophilia	194367710	WCDEYFFLRHRNETRGVGGLFFDDLH		GDFERDFDYLRAVGDGFLDAYLPI
	Stenotrophomonas sp. SKA14	254523147	--------------------------		------------------E-----
	Xanthomonas albilineans	285016941	------Y-K-----------------		A------A-Q--------------
	Xanthomonas campestris	21233441	-------------------------G		Q------A-Q--------------
	Xanthomonas fuscans	294626789	-------------------------G		Q------A-Q--------------
	Xanthomonas axonopodis	21244826	-------------------------G		Q------A-Q-----------I--
	Xanthomonas oryzae	166710295	--------C----------------G		KE--H--A-QQ---N------M--
	Xanthomonas gardneri	325923783	-------------------------D		K------A-Q--------------
	Xanthomonas vesicatoria	325917394	-------------------------G		Q------A-QQ---N------M-L
	Pseudoxanthomonas spadix	357415769	---R---I-----------------Q		D--A---G--Q-------------
	Pseudoxanthomonas suwonensis	319788386	---D----K-----------------		E------T----------------
	Xylella fastidiosa	182680587	------V------------------D		K--------Q-----------F--
	Rhodanobacter sp. 2APBS1	352080887	------Y-K--D---------Y---N	E	-G---C-AFT-D--Q---------
Other γ-Proteobacteria	Acinetobacter baumannii	213158863	---D--Y-K--D-Q-----------N	C	W---TC-K-IQ---N-Y-N-I---
	Acinetobacter calcoaceticus	262280253	---D--Y-K--D-Q-----------N	G	W---TC-K-IQ---N-Y-N-I---
	Alteromonadales bacterium	119471687	------Y-K--D-------------N	E	LG--QS-AFMQS--N------I--
	Candidatus Vesicomyosocius	148244605	---D--Y-K----Q--I--------N	E	-G-DKC-AFM-S----YIK--Q--
	Chromohalobacter salexigens	92114980	------T-H--D-------------N	E	WE---C-AFQ-----A--E--V--
	Citrobacter sp. 30_2	237732430	---D----K----Q--I--------N	T	P--DHC-AFMQ---Q-YTE-----
	Colwellia psychrerythraea	71281546	------Y-K--D-------------N	Q	W--DTC---VK---Q--I---V--
	Cronobacter sakazakii	156933010	---D--Y-K--Q-A--I--------N	T	P--DTC-AFMQ-----Y-N-----
	Dickeya dadantii	307129661	---D----K----A-----------N	A	P--DTS-AFM----E--RH-----
	Enterobacter sp. 638	146312598	---D--Y-K--D-Q--I--------N	A	P--DTA-NFMQ---Q-YT------
	Erwinia amylovora	292488970	---D--H-K----Q--I--------N	A	EG--QS-RFMQ---R--S------
	Escherichia coli	284922390	------Y-K----Q--I--------N	T	P--DHC-AFMQ-I-K-YT------
	Grimontia hollisae	262273072	---R----P----------------N	Q	WG-DAS-G-IK---E-Y-------
	Hahella chejuensis	83642947	---D--Y-K--D-A-----------N	A	WG---S-EFM----NSY-P--S--
	Marinobacter aquaeolei	120552990	---D--Y-K--Q-P----------YN	T	---D---AFMQ----SYIE--E--
	Nitrococcus mobilis	88811390	------Y-P----P----------FT	G	AG--HA-GFM-S--N---Q-----
	Pantoea sp. aB	304398331	---D--Y-K--D-Q--I--------N	T	P---QS-SFMQS--R--I---R--
	Pectobacterium atrosepticum	50119816	---D----K----A--I--------N	E	P--ATS-AFI----N----G----
	Photobacterium damselae	269103769	---R----P-------I--------N	Q	WE-DNC-A-IQ---Q-Y-E-----
	Photobacterium profundum	90413786	---K----P----------------N	E	WG--QS-A-TQ---N--I-G----
	Photobacterium sp. SKA34	89074768	---K----P----------------N	Q	WG-DKS-A-MQ---N--IN--M--
	Proteus mirabilis	227356205	---D--Y-K----P-----------N	T	P--DHC-EFVQH--Q-Y-------
	Providencia rettgeri	268593177	--------K----P-------Y---N	Q	-G--SC-NFTQ---N------V--
	Salmonella enterica	161612804	---D----K----Q--I--------N	T	P--DHC--FMQ---N-YTR-----
	Serratia odorifera	270262972	---D---IK----A--I--------N	T	P--DSC-AFT----E---------
	Shewanella amazonensis	119773192	---K----P--G-------------N	H	WE-DKC---MQ---N---T--A--
	Shigella boydii	187731800	---D--Y-K----Q--I--------N	T	P--DHC-AFMQ---K-YT------
	Sodalis glossinidius	85059690	---D----K----A--I--------S	A	PN-NEA-AFC---SQ-Y-------
	Teredinibacter turnerae	254784310	------Y-K--Q-P--I--------N	H	WG-N-S-AFMQS---SYCR-----
	Vibrio alginolyticus	91226312	---K----P----------------N	Q	WE-DKC-A-IK---E-YCQ-----
	Vibrionales bacterium	148978489	---K--Y-P--D-------------N	E	WG--KS-A-MQ---E-YAA-----
	Xenorhabdus bovienii	290475992	---D---IK----P--I----Y---N	T	P---TC-NFTQ---N---A-----
	Yersinia mollaretii	238799202	---D--YIK--H-A--I--------N	S	P--DTC-SFTQ-------------
	Yersinia rohdei	238752038	---D--YIK----A--I-------FN	S	P--DTC-KFTQ-------------
α-Proteobacteria	Brevundimonas subvibrioides	302383839	------M-P----M--I--I-Y-HHD	S	--YDK--AFT-D--MA-I-T-AA-
	Methylobacterium extorquens	254559258	------H-K----P--I--I-Y-YHW	T	--PQA-LA-T-D--GA--K--PE-
	Brevundimonas sp. BAL3	254419937	--------P--Q-P--T--I-Y-HHD	S	--HD---AFT-D--LA---V-PK-
β-Proteobacteria	Lutiella nitroferrum	224823560	--------K--H-A-----------N	A	WG--QS-AF-Q---N-Y-------
	Achromobacter piechaudii	293604472	--------K-------I--V-----N	A	PG-DAS-ALT-S---A---S----
	Candidatus Accumulibacter	257092428	--------K----P-----V-----N	E	-G---C-GLT-----A-AA-----
	Achromobacter xylosoxidans	338780270	--------K-------I--I-----N	A	LG-DAS-ALT-S---A---S----
	Chromobacterium violaceum	34496212	------H-K----A--I--------N	E	WG-DKS-AFT------Y-------
	Burkholderia glumae	238028185	--------K-------I--I-----S	E	PG---S-ALMQS---A--G-----

Figure S25

Partial sequence alignment of a conserved region of coproporphyrinogen III oxidase showing a 1 aa deletion that is commonly shared by all of Xanthomonadales except *Rhodanobacter* sp. 2APBS1.
