# Supplementary material for: Phylogenomics and Molecular Signatures for Species from the Plant Pathogen-Containing Order Xanthomonadales
Source: PLoS One. 2013 Feb 8;8(2):e55216. doi: 10.1371/journal.pone.0055216 (PMC3568101; doi:10.1371/journal.pone.0055216)
Supplement: Figure S26 — Partial sequence alignment of a conserved region in 2-oxoglutarate-dehydrogenase E1 component, showing a 1 aa deletion that is uniquely shared by Xanthomonadales except Rhodanobacter sp. 2APBS1. (PDF) [file pone.0055216.s026.pdf]

Group	Organism	Accession	106		164
Xanthomonadales	Stenotrophomonas maltophilia	194366403	DPLGLAAPVNTPDLGLPFHSLSDGDLNSEFSTG		GVGGQPRMKLRDLLARLKATYTGSIG
	Stenotrophomonas sp. SKA14	254522086	-----TT--------------------------		--------------------------
	Xylella fastidiosa	71274692	------P-S-P---D----N--QA--DN-----		-I---------N---H------DT--
	Xanthomonas albilineans	285018444	-----TP-I-P-----------ES--G------		-L------------------------
	Xanthomonas oryzae	188577095	-----TP---P-----------QA-MD------		----------K----------AST--
	Xanthomonas axonopodis	21107714	-----VP---P---D-------QA--D------		----------K---T------AS---
	Xanthomonas fuscans	294666496	-----VP---P---D-------QA--D------		----------K---T------AS---
	Xanthomonas campestris	289663045	-----TP---P---D-------QA-MD------		----------K---T------AS---
	Xanthomonas perforans	325925702	-----VP---P---D-------QA--D------		----------K---T------AS---
	Xanthomonas vesicatoria	325914050	-----TP---P---D-------QA--D------		----------K----------AS---
	Xanthomonas gardneri	325919392	-----TP---P---D-------QA-MD------		----------K----------AST--
	Pseudoxanthomonas suwonensis	319787382	-----TP-M-P-----E-----ER--DA-----		-LA-----------------------
	Pseudoxanthomonas spadix	357417263	-----SE---P-----EW-T--ER--D------		--A-----V-----------------
	Rhodanobacter sp. 2APBS1	352081385	-----TEKMPA-----A--G---A--DT--D--	N	YA--GQ-L---E--SH-QK--AST--
Other Bacteria	Aeromonas hydrophila	117618342	-----W-RETVAE-DPA--N-QGA-MEAT-NV-	S	YAI-RET-V-S--Y-S--K--C----
	Alcanivorax sp. DG881	254428530	----IMEREAV---E-AH-G--TA--DTV-Q--	N	LFI-K-ETS---IVDC-TS--CS-V-
	Alteromonas macleodii	239993976	-----WKQERVR--E-SH----EQ-FD-V-NV-	S	YAI-KDS-P-GE-FKS-NR--C----
	Candidatus Hamiltonella	238898944	-----WEREPV---N-AY-D-TES-FENS-NI-	S	FAIPKKT-T-A--YTA--K--C----
	Cellvibrio japonicus	192359900	-----MQRDPV---D-S--G-TNA--DTV----	S	LFI-KEEAT--EIVEA-EK--C-H--
	Citrobacter koseri	157146668	-----WEQE-VA--DPS--D-TEA-FQES-NV-	S	FAI-KET---GE-ISA--Q--C-P--
	Colwellia psychrerythraea	71279902	-----WQRDKVR--Q-SH-D--EN-FDK-YNV-	S	FAI--DT---G--YKA-RN--C----
	Cronobacter sakazakii	156934782	-----WQQERVA--DPA--D-TEA-FQET-NV-	S	FAL-KET---G--IEA--Q--C----
	Dickeya dadantii Ech703	242238590	-----WKQERV---D-AY-N-TEQ--DET-NV-	S	FAI-RDT-R-GE-Y-A--Q--C-P--
	Enterobacter cancerogenus	261341312	-----WQQERVA--DPA--D-TEA-FQET-NV-	S	FAI-KDT---GE-IDA--Q--C----
	Erwinia tasmaniensis	188534431	-----WKQEPVA--DPGY-G---A-FQES-NV-	S	FAI-KET---A--Y-A--Q--C----
	Escherichia fergusonii	218549710	-----WQQEKVA--DPS--D-TEA-FQET-NV-	S	FAS-KET---GE--EA--Q--C-P--
	Halomonas elongata	307217726	-----RS-TPV---D-S--Q--QS--DT--Q--	S	FFL-ADKAP-KEIVEA-QQ--CR---
	Idiomarina baltica	85711263	-----WKQEEV---S-A--D-TKD--DR--NV-	S	LAV-SET---K-IHKA-EDI-C-P--
	Klebsiella pneumoniae	206575964	-----WKQERVA--DPAY-D-TEA-FQDSYNV-	S	FAI-KDT---G--I-A--Q--C----
	Oceanospirillum sp. MED92	89095249	-----MQREPV---D-R--E--EA-YDTV-QL-	S	LFF-PEEAP---IV-D--K--CSTV-
	Pantoea sp. aB	304395707	-----WKQDRVA--DPA--D-TEA-FQES-NV-	S	FAI-KDT---A--FQA-TQ--C----
	Photobacterium profundum	90411599	-----WEQERVLE-APE--N-TED-F-ES-NV-	S	FAV--ET---SEIY-A--K--C----
	Photorhabdus asymbiotica	253990448	-----WKQD-V---DPA--N-TEA-FAET-NV-	S	FAI-KET---A--YEA--R--C----
	Proteus mirabilis	197284466	-----WKRESV---DPA--N-TKE-FEET-NV-	S	FAI-KET---G-IYEA--RI-C----
	Providencia alcalifaciens	212712445	-----WKREAV---DPT--N-TKE-FDET-NV-	S	FAI-KET---SE-YEH--RI-C----
	Psychromonas sp. CNPT3	90406987	-----VENAKVK--DPFY-E-EGE-FDAS-NV-	S	Y-A-NE--I-K---TS--S--C-TL-
	Vibrio fischeri	197335361	-----QIHEYVAE-EPA--N-T-E-FSET-NV-	S	FAS--ET---S--YDA-RS--C--V-
	Xenorhabdus bovienii	290474111	-----WKQEPV---DPA--N-TKA-FEET-NV-	S	FAI-KETL--A--YDA--RI-C----
	Yersinia aldovae	238757973	-----WKQESV---DPAY-H-TEA-FQNT-NV-	S	FAI-KET---S--YTA--Q--C----

Figure S26

Partial sequence alignment of a conserved region in 2-oxoglutarate-dehydrogenase E1 component showing a 1 aa deletion that is uniquely shared by all Xanthomonadales except *Rhodanobacter* sp. 2APBS1
