# Supplementary material for: Phylogenomics and Molecular Signatures for Species from the Plant Pathogen-Containing Order Xanthomonadales
Source: PLoS One. 2013 Feb 8;8(2):e55216. doi: 10.1371/journal.pone.0055216 (PMC3568101; doi:10.1371/journal.pone.0055216)
Supplement: Figure S27 — Partial sequence alignment of a conserved region of asparagine synthase b protein that is showing a 4–5 aa insert, unique to Xanthomonadales except Rhodanobacter sp. 2APBS1. (PDF) [file pone.0055216.s027.pdf]

Group	Species	GI number	404		445
Xanthomonadales	Xanthomonas albilineans	285018780	RVPFLDVEFLEVAMRMDAQHKMAGTR	ADGSK	RIEKAVLREAF
	Xanthomonas campestris	289670204	------R---D-------SF--IDKT	SS-AT	-M--G------
	Xanthomonas oryzae	166712717	------R---D-------SF--IDKT	SS-AA	-M--G------
	Xanthomonas fuscans	294665428	------R---D-------RF--IDKT	SS-AT	-M--G------
	Xanthomonas axonopodis	21242186	------R---D-------RF--IDKT	SS-AT	-M--G------
	Xanthomonas perforans	325928403	------R---D-------RF--IDKT	SG-AT	-M--G------
	Xanthomonas campestris	78047046	------R---D-------RF--IDKT	SG-AA	-M--G------
	Xanthomonas gardneri	325921718	------R---D-------SF--IDKT	SG-AM	-M--G------
	Xanthomonas vesicatoria	325913967	------R---D-------SF--IDKT	ST-AT	-M--G---Q--
	Xylella fastidiosa	71275914	------R---D-----------VDKT	SN-PQ	-M--GI--A--
	Pseudoxanthomonas spadix	357417890	------R---D-------S---ITVG	---K-	-----------
	Pseudoxanthomonas suwonensis	319786523	------R---D-------KY--VLKG	S--PQ	-M--GI--Q--
	Stenotrophomonas sp. SKA14	254525303	------R---D----F--A---V-AG	F-GR	-----------
	Stenotrophomonas maltophilia	194365058	------R---D----F--A---V-AG	F-GR	-----------
	Rhodanobacter sp. 2APBS1	352081994	------L--ID---GL--A-----QG		-----------
Other Bacteria (0/500)	Aeromonas hydrophila	117619348	------K--MD----LQPAD--C-KG		K---HI-----
	Aeromonas salmonicida	145299448	------K--MD----LQPAD--C-NG		K---HI-----
	Aliivibrio salmonicida	209694406	------K--ID----LNP-D--C-NG		KM--HI---C-
	Citrobacter youngae	283834038	------KK--D----INP-D--C-NG		KM--H----C-
	Colwellia psychrerythraea	71278785	------KN-MD----INPED-LC-NG		KM---I---S-
	Cronobacter sakazakii	156934825	------KK--D----INP-D--C-NG		KM--HI---C-
	Dickeya dadantii	271499720	------KK--D----INPKD--C-NG		KM--HI---C-
	Edwardsiella tarda	269139968	------KA--D----LNPRD--C-SG		----QL---C-
	Enterobacter cancerogenus	261341276	------KK--D----INP-D--C-NG		KM--HI---C-
	Escherichia albertii	170767928	------KK--D----INP-D--C-NG		KM--HI---C-
	Escherichia coli	110640885	------KK--D----INP-D--C-NG		KM--HI---C-
	Klebsiella pneumoniae	206575898	------KK--D----INP-D--C-NG		KM--H----C-
	Moritella marina	6691648	------K--VDAS--LNPEL--ITGD		----NII----
	Moritella sp. PE36	149909581	------K--IDT---LNPEL--ITGD		----NII----
	Pectobacterium atrosepticum	50120262	------KN--D----INPRD--C-NG		KM--HI---C-
	Photobacterium profundum	54308220	------K--------INPED--C-NG		KM--HI---C-
	Psychromonas ingrahamii	119944862	------K--MD----TNPEL--IKDG		----NI-----
	Salmonella enterica	167553960	------KK--D----INP-D--C-NG		KM--H----C-
	Shewanella sediminis	157374977	------K--MD----INPEA--SKDG		K---HI--Q--
	Shigella sonnei	74311201	------KK--D----INP-D--C-NG		KM--HI---C-
	Sodalis glossinidius	85058791	------KQ--D----INP-D--C-NG		K---H----C-
	Tolumonas auensis	237808953	------K--MD----LNPKD--C-NG		K---HIV--T-
	Vibrio alginolyticus	91225923	------K--ID----LNPAD--C-NG		KM--HI---C-
	Vibrio cholerae	147673054	------K--ID----LNPAD--C-NG		KM--HI---C-
	Yersinia pestis	22125101	------KN--D----INP-D--C-NG		KM--HI---C-

Figure S27

Partial sequence alignment of a conserved region of asparagine synthase b protein that is showing a 4-5 aa insert, uniquely shared by all Xanthomonadales except *Rhodanobacter* sp. 2APBS1. The species belonging to Stenotrophomonas can also be separated from other Xanthomonadales because of having only 4 aa insert at the same position.
