# Supplementary material for: Phylogenomics and Molecular Signatures for Species from the Plant Pathogen-Containing Order Xanthomonadales
Source: PLoS One. 2013 Feb 8;8(2):e55216. doi: 10.1371/journal.pone.0055216 (PMC3568101; doi:10.1371/journal.pone.0055216)
Supplement: Figure S28 — Partial sequence alignment of a conserved region of Asparagine synthase b protein, showing a 1–2 aa insert that is uniquely shared by Xanthomonadales except Rhodanobacter sp. 2APBS1. While genus Stenotrophomonas can be differentiated from other Xanthomonadales because of having 1 aa insert instead of 2 aa. (PDF) [file pone.0055216.s028.pdf]

			96		132
Stenotrophomonas	Stenotrophomonas maltophilia	194365058	LAFQTGSDCEVINALYRQG	GT	PAQWLEQLNGIFAFALWD
	Stenotrophomonas sp. SKA14	254525303	-------------------	-S	------------------
Xanthomonadales	Xanthomonas campestris	21230846	-----------------ED	A	--SY-NR-----------
	Xanthomonas fuscans	294665428	-----------------ED	-	--SY-NR-----------
	Xanthomonas axonopodis	21242186	-----------------ED	-	--SY-NR-----------
	Xanthomonas oryzae	166712717	-----------------ED	A	-DSY-NR-----------
	Xanthomonas albilineans	285018780	-----------------EQ	A	-VAL-DR-----------
	Xylella fastidiosa	15836723	-----H-----------EE	-	-TSF-NR-----------
	Pseudoxanthomonas spadix	357417890	-----------------ED	A	-ESF-NR-----------
	Pseudoxanthomonas suwonensis	319786523	-----A-----------ED	E	--SF-NR-----------
	Rhodanobacter sp. 2APBS1	352081994	---T-------------EQ		GSDFVGK-----------
Other Bacteria	Aeromonas hydrophila	117619348	-----H-----LL---KEK		GPAF-DD-------I-Y-
	Aeromonas salmonicida	145299448	-----H-----LL---KEK		GPAF-DD-------I-Y-
	Aliivibrio salmonicida	209694406	-----D------L----DK		G-DL--D-------I-Y-
	Alteromonas macleodii	239996399	---K-R------LP-FQ-K		GIDFIDE-E-M---I-Y-
	Candidatus Hamiltonella	238899130	-----D------L---QEK		GEPF-DE-Q-M-T-I-Y-
	Citrobacter koseri	157146724	------------L---QEK		GVEF-DD-Q-M-----Y-
	Cronobacter turicensis	260597080	------------L---QEK		GPEF-DE-Q-M---V-Y-
	Dickeya dadantii	271499720	------------L---QEK		GPEF-DE-R-M-----Y-
	Edwardsiella ictaluri	238920794	------------L---QEY		GPDF-D--R-M---I-Y-
	Enterobacter cloacae	296103380	------------L---QEK		GPEF-DD-Q-M-----Y-
	Erwinia tasmaniensis	188534462	-----D------L---QEK		GVDF-DD-Q-M---I-Y-
	Escherichia albertii	170767928	------------L---QEK		GPEF-DD-Q-M-----Y-
	Escherichia coli	110640885	------------L---QEK		GPEF-DD-Q-M-----Y-
	Klebsiella pneumoniae	206575898	------------L---QEK		GPEF-DD-Q-M-----Y-
	Moritella marina	6691648	-----E------L---KEK		GT-F-DD---------Y-
	Moritella sp. PE36	149909581	-----E----I-L---KEK		GT-F-DD---------Y-
	Pantoea sp. At-9b	258639664	------------L---QEK		GVDF-DD-Q-M---I---
	Pectobacterium atrosepticum	50120262	------------L---QEK		GPEF-DE-R-M-----Y-
	Photobacterium profundum	90411576	-----E----I-L---KEK		GPEL-DY-----G-I-Y-
	Pseudoalteromonas atlantica	109899262	---R-K------LP--E-Q		GVEFID--Q-M---V-Y-
	Psychromonas ingrahamii	119944862	---K-K----I-L---EEK		GCDF-DD-------C-Y-
	Salmonella enterica	167553960	------------L---QEK		GPDF-DD-Q-M-----Y-
	Serratia odorifera	270263726	-----A------L---QEK		GIDF-DD-Q-M-----Y-
	Shewanella amazonensis	119775236	--Y--N------L---QEY		GSDF-DK-------V-Y-
	Shigella sonnei	74311201	------------L---QEK		GPEF-DD-Q-M-----Y-
	Tolumonas auensis	237808953	------------L---KEK		GTAF-DD-S-----I-Y-
	Vibrio alginolyticus	91225923	-----D------L---QDM		G-DL--E-------V-Y-
	Vibrio cholerae	297578599	-----D------L----DK		G-DL--E-------V-Y-
	Yersinia enterocolitica	123443198	------------L---QEK		GPEF-DD-Q-M---V-Y-
	Yersinia pestis Angola	162420654	------------L---QEK		GPAF-DD-Q-M---V-Y-

Figure S28

Partial sequence alignment of a conserved region of Asparagine synthase b protein showing a 1-2 aa insert that is uniquelly shared by all Xanthomonadales except *Rhodanobacter* sp. 2APBS1. While genus Stenotrophomonas can be differentiated from other Xanthomonadales because of having 1 aa insert instead of 2 aa.
