# Supplementary material for: Phylogenomics and Molecular Signatures for Species from the Plant Pathogen-Containing Order Xanthomonadales
Source: PLoS One. 2013 Feb 8;8(2):e55216. doi: 10.1371/journal.pone.0055216 (PMC3568101; doi:10.1371/journal.pone.0055216)
Supplement: Figure S29 — Partial sequence alignment of a conserved region DNA polymerase III subunit alpha showing a 4 aa insert that is commonly shared by Xanthomonadales except Rhodanobacter sp. 2APBS1. This CSI was previously identified by [28] as all Xanthomonadales specific signature. (PDF) [file pone.0055216.s029.pdf]

			522		576
Xanthomonadales	Xylella fastidiosa 9a5c	77747494	TRNAGKHAGGVVIAPNPLTEFCPLFAEH	DENG	RGKNPVTQFDKNDVEEVGLVKFD
	Xylella fastidiosa M12	170729407	----------------------------	----	-----------------------
	Xylella fastidiosa Dixon	71276388	----------------------------	----	-----------------------
	Xylella fastidiosa Ann-1	71900814	----------------------------	----	-----------------------
	Xylella fastidiosa Temecula1	28198096	----------------------------	----	-----------------------
	Xanthomonas oryzae	353462904	---------------E--S---------	--D-	-----------D---A-------
	Xanthomonas vesicatoria	325916626	---------------E--S---------	--D-	-----------D---A-------
	Xanthomonas campestris	289662892	---------------E--S---------	--D-	-----------D---A-------
	Xanthomonas axonopodis	346724307	---------------D--S---------	--D-	-----------D---A-------
	Xanthomonas fuscans	294625959	---------------D--S---------	--D-	-----------D---A-------
	Xanthomonas perforans	325929585	---------------D--S---------	--D-	-----------D---A-------
	Xanthomonas gardneri	325923963	---------------E--S---------	--G-	----Q------D---A-------
	Xanthomonas albilineans	285018805	---------------T--SD--------	-VDH	L----------D---Q-------
	Stenotrophomonas sp. SKA14	254524078	-------------G-E--S-----Y---	----	L----------------------
	Stenotrophomonas maltophilia	194365029	-------------G-E--S-----Y---	----	L----------------------
	Pseudoxanthomonas suwonensis	319787804	---------------T--HT--------	-HD-	L-R--------------------
	Pseudoxanthomonas spadix	357417821	---------------S--SD--------	--GT	--R--------D---A-------
	Rhodanobacter sp. 2APBS1	352086322	---------------S---D-A--YC-A		G-GGV---Y--D---A-------
Other Bacteria (0/500)	Alcanivorax borkumensis	110834019	---V-----------GK--D-A--YC-E		D-T-L---Y--D---SA------
	Alteromonas macleodii	196156206	---------------TTI-D-S--YCDD		E-N------------TA------
	Arsenophonus nasoniae	284008505	---------------TKI-D-A--YCDP		E-Q--L---------YA------
	Citrobacter koseri	157147384	---------------TKI-D-A--YCDE		A-QH-----------YA------
	Coxiella burnetii	212212283	---------------TK--D-V--YS-P		GSDHV------D---A-------
	Enterobacter cancerogenus	261338818	---------------TKI-D-A--YCDE		A-HH-----------YA------
	Erwinia tasmaniensis	188533052	---------------TQI-D-A--YCDE		H-NH-----------YA------
	Escherichia coli	19548812	---------------TKI-D-A--YCDE		E--H-------S---YA------
	Idiomarina baltica	85713117	-------------S-TTI-D-AA-YCDD		E--------------SA------
	Klebsiella pneumoniae	262044745	---------------TKI-D-A--YCDE		Q-LH-----------YA------
	Methylophaga thiooxidans	254491862	---V-----------KV---YT--YC--		N-AGL-S----D---S-------
	Nitrosococcus halophilus	292492697	---------------KK---YV-YYC-Q		GASGV------D-I-AI------
	Pantoea ananatis	291616361	---------------TKI-D-A--YCDE		N-QY-----------YA------
	Photorhabdus luminescens	37524688	---------------TKI-D-S--YCDP		E-L------------YA------
	Proteus mirabilis	227357235	-------------S-TKI-D-A--YCDP		E-N------------YA------
	Providencia rettgeri	291326493	---------------TKI-D-A--YCDA		E-N------------YA------
	Salmonella enterica	161504650	---------------TKI-D-A--YCDE		E--H-------S---YA------
	Serratia odorifera	293394718	---------------TKI-D-A--YCDA		E-NH-----------YA------
	Shewanella amazonensis	119774291	---------------TKI-D-S--YCDA		E--------------YA------
	Shigella boydii	82542783	---------------TKI-D-A--YCDE		E--H-------S---YA------
	Sodalis glossinidius	85059906	---------------TKI-D-A--YCDG		E-NH-----------YA------
	Vibrio parahaemolyticus	254508656	-------------S-TTI-D-A-IY-DA		E-HF-----------TA------
	Xenorhabdus nematophila	300724779	---------------TKI-D-A-IYCDP		E-Q------------YA------
	Yersinia pestis	167471052	---------------TKI-D-A--YCDA		E-N------------YA------
	Nitrobacter hamburgensis	92117240	--H-ST--A-I--GDR--S-LV--YRDP		KSDM-----NMKW--PA------
	Bradyrhizobium japonicum	27379977	--H-ST--A-I--GDR--S-LV--YRDP		KSDM-----NMKW--PA------
	Ralstonia pickettii	241663600	---V-M-----L---GK--D----YTQG		-EDAGV-S-Y--D---A-------
	Methyloversatilis universalis	334132599	--GV-M-----L---GK--D----YQQD		-DDAS--S----D---AI------
	Pusillimonas sp. T7-7	332283756	---I-M-----L---GK--D----YCQP		-VDTSA-S----D---AA------
	Bordetella avium	187478729	---I-M-----L---GK--D----YCQP		-QENYA-S----D---AA------
	Thauera sp. MZ1T	217970003	S--V-M-----L---GK--D----YIAD		-DDAT--S----D---A-------
	Aromatoleum aromaticum	56477932	S--V-M-----L---GK--D----YIAD		-DEAT--S----D---A-------
	Chromobacterium violaceum	34496366	--GI-M-----L---GR--D----YIAS		-D-AS--S----D---QI------

Figure S29

Partial sequence alignment of a conserved region DNA polymerase III subunit alpha showing a 4 aa insert that is commonly shared by all Xanthomonadales except *Rhodanobacter* sp. 2APBS1. This signature was previously identified by (Cutino-Jimenez et al., 2010) and was described as all Xanthomonadales specific Signature.
