# Supplementary material for: Phylogenomics and Molecular Signatures for Species from the Plant Pathogen-Containing Order Xanthomonadales
Source: PLoS One. 2013 Feb 8;8(2):e55216. doi: 10.1371/journal.pone.0055216 (PMC3568101; doi:10.1371/journal.pone.0055216)
Supplement: Figure S30 — Partial sequence alignment of a conserved region of RecA showing a 2 aa insert that is commonly shared by Xanthomonadales except Rhodanobacter sp. 2APBS1. This CSI was previously identified by [28] as all Xanthomonadales specific signature. (PDF) [file pone.0055216.s030.pdf]

```
                                                      172                                    238
Xanthomonadales
Xylella fastidiosa 9a5c          15836728    QALRKLTGNIKRSNTLVIFINQLRMKIGVM MP GQSPETTTGGNALKFYASVRLDIRRIGAIKKGDEI
Xylella fastidiosa Dixon         71275910    ----------------------------I- -- -----------------------------------
Xylella fastidiosa M12           170729337   ----------------------------I- -- -----------------------------------
Xylella fastidiosa Temecula1     28198030    ----------------------------I- -- -----------------------------------
Stenotrophomonas maltophilia     190573723   ------------------------------ -- -----------------------------------
Stenotrophomonas sp. SKA14       254524001   ------------------------------ -- -----------------------------------
Xanthomonas albilineans          285017962   -----------------V------------ -- -----------------------------------
Xanthomonas oryzae               2460018     -----------------V------------ -- -----V-----------------------------
Xanthomonas vesicatoria          325916032   -----------------V------------ -- -----V-----------------------------
Xanthomonas campestris           289669642   -----------------V------------ -- -----V-----------------------------
Xanthomonas gardneri             325924502   -----------------V------H----- -- -----V-----------------------------
Xanthomonas axonopodis           21242489    -----------------V------------ -- -----V-----------------------------
Xanthomonas fuscans              294626629   -----------------V------------ -- -----V-----------------------------
Pseudoxanthomonas suwonensis     319786626   ------------------------H----- -- ---------------------------S-------
Pseudoxanthomonas spadix         357417030   ------------------------------ -- -----V-----------------------------
Rhodanobacter sp. 2APBS1         352081858   -------A---K-GC-------I-------    FG--------------------------V---E--
Other Bacteria (0/500)
Pseudoalteromonas atlantica      109899564   ----------------------I-------    FG----------------------------D---V
Nitrosococcus watsonii           300113490   -------A--------------I-------    FG-----------------------V--L------
Methylomonas methanica           333985518   -------A----------------------    FGN-------------------------------V
Methylobacter tundripaludum      344943375   ----------------------I-------    FG-------------------------S----E-V
Legionella pneumophila           464597      -------A--------------I-------    FG-------------------------S----E--
Dichelobacter nodosus            146328886   ------------A----V----I-------    FG------------------M------S--E---V
Vibrio harveyi                   213012346   --M------L-Q--CMC-----I-------    FGN----------------------T----E---V
Pseudomonas putida               18076865    -----I-----NA-C-------I-------    FG-----------------------T--V-E---V
Gallionella capsiferriformans    302877752   -------A----------------------    FGN-------------------------------V
Methylotenera mobilis            253997269   ------------T---------I-------    FGN----------------------T---------
Dechloromonas aromatica          71909761    -------A---KT---------I-------    FG--------------------------------V
Lutiella nitroferrum             224825851   ------------T---------I-------    FG-------------------------G----E--
Cupriavidus sp. mapud3.4         304334137   --------T---T-C-------I-------    FG-------------------------S-----DV
Laribacter hongkongensis         226939722   ------------T---------I-------    FGN----------------------------T---
Cupriavidus sp. mapud10.1        304334135   --------T---T-C-------I-------    FG-------------------------S-----DV
Methylotenera versatilis         297539361   ------------T--M------I-------    FGN----------------------T--------V
Chromobacterium violaceum        61097689    ------------T---------I-------    FGN----------------------T-G------V
Methylomonas clara               132226      ------------T---------I-------    FGN-------------S--------T--------V
Neisseria mucosa                 2500090     --------H---T----V----I-------    FG-----------------------T-Q-----DV
Chromobacterium piscinae         61097707    ------------T---------I-------    FGN----------------------T-G----E-V
Chromobacterium violaceum        61097691    ------------T---------I-------    FGN----------------------T-G----E-V
Ralstonia solanacearum           83746096    --------T---T-C-------I-------    FG-------------------------S--R---V
Neisseria flavescens             2500089     --------H---T----V----I-------    FG-----------------------T-Q-----DV
Burkholderia sp. JPY157          262112670   --------T---T-C-------I-------    FGN------------------------S---N--V
Ralstonia solanacearum           300692434   --------T---T-C-------I-------    FG-------------------------S--R---V
```

Figure S30

Partial sequence alignment of a conserved region of RecA showing a 2 aa insert that is commonly shared by all Xanthomonadales except *Rhodanobacter* sp. 2APBS1. This signature was previously identified by (Cutino-Jimenez et al., 2010) and was described as all Xanthomonadales specific Signature.
