# Supplementary material for: Phylogenomics and Molecular Signatures for Species from the Plant Pathogen-Containing Order Xanthomonadales
Source: PLoS One. 2013 Feb 8;8(2):e55216. doi: 10.1371/journal.pone.0055216 (PMC3568101; doi:10.1371/journal.pone.0055216)
Supplement: Figure S31 — Partial sequence alignment of a conserved region of MutS showing a 5 aa insert that is commonly shared by Xanthomonadales except Pseudoxanthomonas suwonensis and Rhodanobacter sp. 2APBS1. This CSI was previously identified by [28] as all Xanthomonadales specific signature. (PDF) [file pone.0055216.s031.pdf]

			765		806
Xanthomonadales	Xylella fastidiosa 9a5c	15838317	LFATHYFELTALADETY	EGGLS	GIANVHFDAVEHSERLVFMH
	Xylella fastidiosa Dixon	71275401	-----------------	-----	--------------------
	Xylella fastidiosa Ann-1	71901697	-----------------	-----	--------------------
	Xylella fastidiosa M23	182681688	-------------E---	-----	--------------------
	Xylella fastidiosa Temecula1	28198974	-------------E---	-----	--------------------
	Xanthomonas oryzae pv. oryzae	58581265	---------------SH	D--A-	------L-----G-------
	Xanthomonas campestris pv. vas	289662777	---------------SH	A--A-	------L-----G-------
	Xanthomonas gardneri ATCC 1986	325919899	---------------SH	A--A-	------L-----G-------
	Xanthomonas axonopodis pv. cit	346724192	---------------SH	A--A-	------L-----G-------
	Xanthomonas perforans 91-118	325924847	---------------SH	A--A-	------L-----G-------
	Xanthomonas vesicatoria ATCC 3	325915949	---------------SH	A--A-	------L-----G-------
	Xanthomonas albilineans GPE PC	285018993	---------------PV	---P-	------L-----GD------
	Xanthomonas fuscans subsp. aur	294664088	--------------ASH	A--A-	------L-----G-------
	Stenotrophomonas maltophilia J	344206568	---------------QH	---R-	------L-----G-A-----
	Stenotrophomonas sp. SKA14	254522797	---------------QH	---R-	------L-----G-A-----
	Pseudoxanthomonas spadix BD-a5	357417002	-------------S-SF	---P-	------L-----GHT-----
	Pseudoxanthomonas suwonensis 1	319786617	----------S--TPES		------L-----GDS-----
	Rhodanobacter sp. 2APBS1	352081856	----------E--N-FA		A-----L----
Other Bacteria (0/500)	Cellvibrio japonicus	192360641	--------I-S-PETVP		--V---LN-T--QDKI--L-
	Citrobacter youngae	283835500	----------Q-PEKME		-V----L--L--GDTIA---
	Colwellia psychrerythraea	71282195	----------L--GQIS		TL----L--M--DDNI----
	Escherichia coli	21951852	----------Q-PEKME		-V----L--L--GDTIA---
	Haemophilus influenzae	45645383	------------PEQLE		----I-L--L--NNTIA---
	Halorhodospira halophila	121998436	--------M---EQIHP		-VV---LE-A--G--I--L-
	Marinobacter algicola	149374515	----------Q---DLE		HAV---LT-T--DDTI--L-
	Methylococcus capsulatus	53805175	-------------E-CE		DVG---L-----GDKV--L-
	Nitrosococcus watsoni	300113486	------------PEHLD		-V--L-LT-T--K--I--L-
	Oceanospirillum sp. MED92	89092115	----------S-P-QAA		NVF---LT----NDHI--L-
	Pseudoalteromonas atlantica	109899568	----------G---ALP		EL----L-----G-SIR---
	Psychromonas ingrahamii	119946988	----------K-PENIP		ELV---L-----GDAIA-L-
	Salmonella enterica	21951842	----------Q-PEKME		-V----L--L--GDTIA---
	Shewanella woodyi	170727679	----------Q-PELIS		NV----L--I--GDTI----
	Shigella dysenteriae	82778101	----------Q-PEKME		------L--L--GDTIA---
	Tolumonas auensis	237809472	----------Q--EQQA		N-V------I--GDSVA---
	Yersinia aldovae	238758208	----------T-PEKME		-VV---L--M--G-TIA---
	Yersinia mollaretii	238797129	----------T-PEKME		-VV---L--L--G-TIA---

Figure S31

Partial sequence alignment of a conserved region of MutS showing a 5 aa insert that is commonly shared by all Xanthomonadales except *Pseudoxanthomonas suwonensis* and *Rhodanobacter* sp. 2APBS1. This signature was previously identified by (Cutino-Jimenez et al., 2010) and was described as all Xanthomonadales specific Signature.
