# Supplementary material for: Phylogenomics and Molecular Signatures for Species from the Plant Pathogen-Containing Order Xanthomonadales
Source: PLoS One. 2013 Feb 8;8(2):e55216. doi: 10.1371/journal.pone.0055216 (PMC3568101; doi:10.1371/journal.pone.0055216)
Supplement: Figure S32 — Partial sequence alignment of a conserved region of DNA polymerase III subunit alpha, showing a 2 aa deletion that is commonly shared by all Xanthomonadales except Pseudoxanthomonas suwonensis and Rhodanobacter sp. 2APBS1. These two have only 1 aa deletion at the same position. (PDF) [file pone.0055216.s032.pdf]

			65		120
Xanthomonadales	Stenotrophomonas maltophilia	194365029	KFYKAAEGVGIKPIAGADVLIAEEGQD		PWRMTLLCRDREGYLSLSRLLTRAWMEG
	Stenotrophomonas sp. SKA14	254524078	---------------------------		----------------------------
	Xanthomonas oryzae	166712747	------------------M--TPDMT		----------------------------
	Xanthomonas axonopodis	21242159	------------------M--TPDMT		----------------------------
	Xanthomonas campestris	188992333	------------------M--TPDMT		----------------------------
	Xanthomonas fuscans	294625959	------------------M--TPDMT		----------------------------
	Xanthomonas albilineans	285018805	-----------------L---DGS-A		---L-V---N-D------------L--
	Xanthomonas perforans	325929585	------------------M--TPDMT		----------------------------
	Xanthomonas vesicatoria	325916626	------------------M--TPDMT		----------------------------
	Xanthomonas gardneri	325923963	------------------M--TPDMT		----------------------------
	Xylella fastidiosa	71898629	-------T------S---L----PEHT		--G-------HT---N--Q-IS-G-L--
	Pseudoxanthomonas spadix	357417821	-Y---C-A----------S-V--DGT		--SI------HS---T---------L--
	Pseudoxanthomonas suwonensis	319787804	-----C-A-----V----LM--SSPGE	A	-----A-V--HG---N-----------
	Rhodanobacter sp. 2APBS1	352086322	-----CSAA-----G-C-LWM-APDDP	R	---L----QH-A---N----VS---Q--
Other Bacteria	Acidithiobacillus	255020984	---Q--REA-V-A-I-SE-WLCPPD-P	ED	-SSLV------T-FRN-----S-GYL--
	Acidithiobacillus	218665927	---N--R-Q-V--LI-SE-WVHDVAEP	ER	-VSLV---M-KT--RN-----S--YL--
	Alkalilimnicola ehrlichii	114321000	------LAA-V---V--EIRVED-EAE	RD	HSAL------LD--AA-----SLSYQQ-
	Allochromatium vinosum	288940218	R-----V-A-V-------LWVRNPEDA	NH	-H-LV--AQ-EP--RN-T--IS-GFV--
	Arsenophonus nasoniae	284008505	---G--HAE-----I---FYLES-LLG	DE	VAHL-I-A--NK--HN-TL-ISA-YQK-
	Azotobacter vinelandii	226945926	------MAG-V---C---IWL-SAEP-	AP	LS-L---AM-AK--RN-TE-VS-G-T--
	Cellvibrio japonicus	192360327	------Q-T-------S-F-M-SHET-	GK	-TLL---AMNNQ--KNIIE-IS---RS-
	Chromohalobacter salexigens	92112711	-T----Q-A-L--VI---LWMENPHDE	EH	-Y-V---AM-ND--RH-TE-ISQG-S--
	Congregibacter litoralis	88706741	--H---VNA-----F----WVT-SLK-	DE	AHPLC--VMNAT---N-T---S--YR--
	Edwardsiella ictaluri	238918790	---GS-H-A----LV-----VRS-TMG	DE	LSAL---AC-NQ--QN-TQ-ISH-YQR-
	Edwardsiella tarda	269138108	---GS-H-A----LV-----VRS-TMG	DE	LSAL-I-AC-N---QN-TQ-ISH-YQR-
	Halorhodospira halophila	121998235	---R--LAA-V---I-G--WL-DDTVE	FG	YA-L-------G--RA-----S--YR--
	Marinobacter algicola	149377463	R-----M-A-V---I---LWVENPDEP	DN	-F-I---A-NND---N-TEVVSLGYT--
	Methylophaga thiooxidans	254491862	-LFN--MQA-V---L---LRLKDPSDA	KS	ST-FV---MNL--FHN-----S--Y---
	Nitrococcus mobilis	88812392	---R--VNA-V---I--EIRV-DADAS	ER	-A-LV---A-TQ--RT-T-----GYL--
	Proteus mirabilis	227357235	---G--H-A-----V----YLET-LLG	DE	YAHL-I-A--NV--QN-TL-ISE-YKH-
	Providencia rettgeri	291326493	R--G--H-A-----I---FYVES-LLG	DE	ISHL-V-A-NN---QN-TL-ISE-YQK-
	Pseudomonas mendocina	146308059	----T-M-A-V---C---IWM-SAEE-	GP	LT-L---AMNPK--RN-TE-IS-G-A--
	Saccharophagus degradans	90020753	------Q-A-V---C-C-FRMEPLDEE	SN	-AIF---AMNP---RNITE-ISLSYQK-
	Yersinia pestis	167470458	---GS-H-A-----I---FYVQS-ILG	DE	LAHL-V-A-NN---QN-TL-ISE-YQR-

Figure S32

Partial sequence alignment of a conserved region DNA polymerase III subunit alpha showing a 2 aa deletion that is commonly shared by all Xanthomonadales except *Pseudoxanthomonas suwonensis* and *Rhodanobacter* sp. 2APBS1. These two have only 1 aa deletion at the same position.
