# Supplementary material for: Phylogenomics and Molecular Signatures for Species from the Plant Pathogen-Containing Order Xanthomonadales
Source: PLoS One. 2013 Feb 8;8(2):e55216. doi: 10.1371/journal.pone.0055216 (PMC3568101; doi:10.1371/journal.pone.0055216)
Supplement: Figure S33 — Partial sequence alignment of tRNA (guanine-N(1)-)-methyltransferase, showing a 2 aa insert that is commonly present in all members of Xanthomonadales except Pseudoxanthomonas suwonensis and Rhodanobacter sp. 2APBS1. (PDF) [file pone.0055216.s033.pdf]

Group	Species	ID	140		200
Xanthomonadales	Stenotrophomonas maltophilia	194364933	GELGAAVIIDAVARLQDGALNDAESAAQDSFEG	DL	GLLDCPHYSQPAQHPLGDVPEVLRS
	Stenotrophomonas sp. SKA14	254523049	---------------------------------	--	-------------------------
	Xanthomonas campestris	289662769	-------VV-V-T-----V--------------	PQ	---------H--T-AW----D----
	Xanthomonas oryzae	166713046	-------VV-V-T---E-V--------------	PQ	---------H-SS-AW---------
	Xanthomonas axonopodis	21242047	-------VV-V-------V--------------	PQ	---------H--T-AW----D----
	Xanthomonas albilineans	285017748	---A---LV--LT---E----------------	PD	--------TH-RM-A--E--A----
	Xanthomonas vesicatoria	325914812	-------VV-V-T-----V--------------	PQ	---------H-ST-AW----D----
	Xanthomonas gardneri	325919893	-------LV-V-T---E-V--------------	PQ	---------H-ST-DW----D----
	Pseudoxanthomonas spadix	357416994	---A---L----T---E-V--------------	EG	-------FTH--S-AW-E-------
	Xylella fastidiosa	71275920	-------VV---T---E-V-------K-----A	AD	S---Y---TH-SN-AF-N-------
	Pseudoxanthomonas suwonensis	319786605	---A--IVV-T-----------G--E-----D		---------H-VE-AY-V--A----
	Rhodanobacter sp. 2APBS1	352081083	---A---V----G---E---H--Q--E----AD		--------AR-VHDA--S--D--L-
Other γ-Proteobacteria	Alteromonas macleodii	196156516	---P-M-LM------VP-V-GHKA--VE---TD		--------TR-EILDGQK-----L-
	Arsenophonus nasoniae	284007746	---P-M-M--SIS-FIP-V-GHHA--EE---MT		--------TR-EVLEGME--R--L-
	Azotobacter vinelandii	226945995	---P-M-L----T--LP---GH-D--EE---TD		--------TR-EVYEGKR-----L-
	Cellvibrio japonicus	192359118	---P-M-L---IT--VP---GH-Q--E----TD		--------TR-EEFEGLR--D--V-
	Citrobacter rodentium	283786227	---P-M-L--S-S-FIP-V-GHEA--TE---AD		--------TR-EVLEEME--A--L-
	Dickeya zeae	251788624	---P-MTL--S---FIP-V-GHEA--QE---AD		--------TR-EVLEGM---A--L-
	Edwardsiella ictaluri	238921086	---P-MTL--SIS-FVP-V-G-QA--EE---VD		--------TR-EVLQDME--P--L-
	Enterobacter cancerogenus	261342038	---P-MTL--S---FIP-V-GHEA--TE---AD		--------TR-EVLEGM---A--L-
	Erwinia billingiae	300718045	---P-MTL--S---FIP-V-GKQA--DE---SN		--------TR-EVLEGME--P--L-
	Grimontia hollisae	262273652	---P-MTLV-----FVP-V-G-LA--EE---AN		--------TR-EVLDGLE--S--K-
	Hahella chejuensis	83644608	--IA-MA---S-S--AP-V-GHN---E-----N		S-------TR-EIYRDKR-----L-
	Idiomarina baltica	85712462	---P-M-L------MVP-V-GHQD---E---AN		--------TR-ETYNGQQ--T--L-
	Legionella longbeachae	270158992	---A-T-F---II--IP-S-GHLC--E----MN		--------TR--EVDGLG--P--LG
	Methylophaga thiooxidans	254492628	---A-M-M--G----LP-V-G-EN--E----MT		-F------TR-EKLFDQS-----LG
	Nitrosococcus oceani	77165721	---A-M----GL---LP-V-GA----Q----VN		--------TR-EVIEGYR--P--L-
	Oceanospirillum sp. MED92	89091993	---P-MTM--S-S--VP-V-GHQD--TE---HD		--------TR-E-FEDMQ-----L-
	Providencia rustigianii	261346216	---P-M-L--S-S-FIP-V-GHQA--QE---AD		--------TR-EVLDGMQ---I-L-
	Pseudomonas aeruginosa	116051741	---P-M-LV---T--LP---GH-D--EE---TD		--------TR-EVYADKR-----L-
	Rickettsiella grylli	160871788	---A-MCL---IT---P---G-EK--S----HS		A---Y---TR--SLEEKN--A--LQ
	Shewanella baltica	126173450	---P-MTL--S-S--VP-V-GKQA--E----SD		--------TR-ESLDGV---A--L-
	Teredinibacter turnerae	254785311	---P-M-LL--LI-KLP---G-EL--Q-----T		--------TR-EKVGEDE--A--M-
	Tolumonas auensis	237809702	---P-MTLV---S--VP-V-G-MA--E----TD		--------TR-ESLDGVV--D--L-
	Vibrio fischeri	59711158	---P-MTL--S-S-FVP-V-G-FA--EE---AD		--------TR-EVLDGKE--S--K-
	Vibrio splendidus	84394292	---P-MTL--S-S-FVP-V-G-FA--EE---AN		--------TR-EVLDDK---S--K-
α-Proteobacteria	Methylobacterium sp. 4-46	170742648	---A-L-L---CV--LP-VMGK-A-GTEE----		---EY---TR-RAWEGRTI----TG
	Mesorhizobium ciceri	319780738	--PA-L-LL---V--LP-VMGN-M-GEEE---N		---EH---TR-QEFEGR-I----I-
	Acetobacter tropicalis	339018068	--TA-L-LL--CV--LP-VMGSD--GVEE--SD		---EY---TR--SWDGR------L-
	Rhodobacter capsulatus	294675921	--IA-Q-V---TV--RE-V-GN----TEE-HSN		---EH-Q-TR--EWHGRAI----M-
	Brucella suis	23502764	--TA-I-LL---V--LP-VMGNR--GETE---T		---EH---TR-QEWEGRAI-DI-T-
β-Proteobacteria	Lutiella nitroferrum	224824768	---P-M-LM---V--LP-V----Q--YE---VD		--------TR-EEYQGMR--DI-M-
	Chromobacterium violaceum	34499128	---P-M-LA---V--LP------Q--YE---VD		--------TR-EEYRGMR--D--L-
	Burkholderia ubonensis	167586559	---P-MAMM---V--LP-V----Q--V----V-		--------TR-EEYEGVR--D--LG
	Neisseria meningitidis	325207635	---P-MMLM---L--VP-V-G-MQ--E----SS		-I------TK-LEFQGMA-------
	Burkholderia thailandensis	167837423	---P-MALM---V--LP-V----Q--V----AD		--------TR-EEYEGAR--D--LG
	Ralstonia pickettii	187927901	--IA-MA-----V-QLP-V-G--Q--V----VN		--------TR-EEYESVR--D--LG
	Thauera sp. MZ1T	237653446	---P-M-LL--IV-QLP----T-G--QE---AD		--------TR-ERYEDER-----L-

Figure S33

Partial sequence alignment tRNA (guanine-N(1)-)-methyltransferase showing a 2 aa insert that is commonly present in all members of Xanthomonadales except *Pseudoxanthomonas suwonensis* and *Rhodanobacter* sp. 2APBS1.
