# Supplementary material for: Phylogenomics and Molecular Signatures for Species from the Plant Pathogen-Containing Order Xanthomonadales
Source: PLoS One. 2013 Feb 8;8(2):e55216. doi: 10.1371/journal.pone.0055216 (PMC3568101; doi:10.1371/journal.pone.0055216)
Supplement: Figure S34 — Partial sequence alignment of a conserved region in glucose-6-phosphate 1-dehydrogenase, showing a 4 aa deletion that is uniquely present in all Xanthomonadales except Pseudoxanthomonas spadix BD-a59 and Rhodanobacter sp. 2APBS1 which has 3 aa insert. (PDF) [file pone.0055216.s034.pdf]

Group	Organism	ID	290		334
Xanthomonadales	Stenotrophomonas maltophilia	190573773	RGRYTAGTINGQPAQAYQPPEGSDV		ETFAGVTAHIDNWRWSGVPF
	Stenotrophomonas sp. SKA14	254521422	-------------------------		--------------------
	Xanthomonas campestris	78047793	-------S---------H-------		---VA----------A----
	Xanthomonas oryzae	166712292	-------S---------H-------		---VA----------A----
	Xanthomonas axonopodis	21242810	-------S---------H-------		---VA----------A----
	Xanthomonas fuscans	294624878	-------S---------H-------		---VA----------A----
	Xanthomonas albilineans	285018600	--------V----------------		---VA---Y------A----
	Xanthomonas perforans	325928380	-------S---------H-------		---VA----------A----
	Xanthomonas gardneri	325920391	-----------------H-------		---VA----------A----
	Xanthomonas vesicatoria	325917568	-----------------H-------		---VA----------A----
	Xylella fastidiosa	28198263	----S-----S--VP--------N-		---V----Y------A----
	Pseudoxanthomonas suwonensis	319786896	-----------E-V---S--DD---		---V-----------A----
	Rhodanobacter sp. 2APBS1	352080567	--Q-R--VAE-AGVPG-PEEL-NSK	SNT	---VALK-E-A----A----
	Pseudoxanthomonas spadix BD-a59	357417100	--Q-A---V---AVP--RDEDTVSP	TSDT	--YVAIKLA--S---A----
Other γ-Proteobacteria	Alteromonas macleodii	239993587	--Q--S-FVK-EEVPG-LEE--ANT	QSKT	---VAIK-E------A----
	Azotobacter vinelandii	226942365	--Q-A--K-G--EVP--YFEKNV-S	DSDT	---VA-H-E------A----
	Candidatus Blochmannia	33519903	--Q----F-----VPG-LEEI--NK	DSKT	---VSIKVN------F----
	Citrobacter koseri	157145373	--Q--T-FAQ-KKVPG-LEE--ANK	SSNT	---VAIRVD------A----
	Colwellia psychrerythraea	71278552	--Q-SD-FL--VSVPG-LNE--ANA	NSNT	---VAIK-E------K----
	Cronobacter sakazakii	156933569	--Q----FAQ-KKVPG-LEE--ANK	TSHT	---VAIRVD------A----
	Dickeya dadantii	307130899	--Q--S-FVQ-KKVPG-LEE--ANK	TSNT	---VAIRVD--D--------
	Edwardsiella tarda	294635846	--Q----FVQ-KKVPG-LEE--ANK	SSNT	---IAIR-D------A----
	Enterobacter cancerogenus	261340238	--Q----FAQ-KKVPG-LEE--ANK	ASNT	---VAIRVD--D---A----
	Erwinia amylovora	292488528	--Q--S-FVQ-KKVPG-LEE--ANK	SSNT	---VSIRVD--D---A----
	Escherichia coli	600735	--Q----FAQ-KKVPG-LEE--ANK	SSNT	---VAIRVD------A----
	Hahella chejuensis	83643249	--Q----N-D-K-VPG-LDE--ANE	GSST	-S-VAIK-E-C----A----
	Idiomarina baltica	85711651	--Q-QG-F-GE-KVPG-LQE--ARE	NSDT	---VALKVN-----------
	Legionella drancourtii	254495818	-AQ-VGNV----KIPG-LDE--AYK	ESTT	---VAIK-F------A----
	Legionella longbeachae	270159029	-AQ-VENV----NVLG-LDE--AHR	SSTT	---VAIK-Y-----------
	Marinobacter algicola	149378411	--Q-----SG-K-VPG-LEE--AAR	GSKT	---VALK-E-----------
	Pantoea ananatis	291617744	--Q--S-FVQ-KKVPG-LEE--ANK	TSAT	---VAIRVD------A----
	Pectobacterium carotovorum	227111595	--Q--S-FVQ-HKVPG-LEE--ANK	SSST	---VSIRVD--D--------
	Photorhabdus asymbiotica	253989923	--Q----FVQ-SKVPG-LEE--ANQ	SSRT	---VSIRVD--D---A----
	Proteus mirabilis	197285018	--Q----FVQ-KKVPG-LDE--ANK	ASNT	---VAIR-D------A----
	Providencia stuartii	183600611	--Q-C--QV--ET-TD-CNEP-IP-	DTNT	----ALRIE------K----
	Pseudomonas entomophila	104784395	--Q-G--K-G--EVP--YFEKDV-N	DSDT	---VA-Q--------AS---
	Salmonella enterica	161502998	--Q----FAQ-KKVPG-LEE--ANK	SSNT	---VAIRVD------A----
	Serratia proteamaculans	157371008	--Q----FVQ-KKVPG-LEE--ANK	SSNT	---VSIRVD----Q-A----
	Shewanella amazonensis	119774947	--Q-S--FLK-S-VPG-LEE--AN-	NSNT	---VALRVD------A----
	Shigella flexneri	30063263	--Q----FAQ-KKVPG-LEE--ANK	SSNT	---VAIRVD------A----
	Sodalis glossinidius	85059248	--Q--G-FVQ-KKVPG-LEE--ANK	NSRT	---VSIRVD------A----
	Thiomicrospira crunogena	78485558	-AQ----E-----VP--LDEP-MAP	DSVT	--Y-ALKLYV-----A----
	Xenorhabdus nematophila	300723115	--Q----FVH-KKVPG-LEE--ANK	ASKT	-S-VSIRVD--D--------
	Yersinia bercovieri	238782520	--Q----FVQ-NKVPG-LEE--ANK	SSNT	---VSIRVD--D---A----
α-Proteobacteria	Magnetospirillum magnetotactic	23006819	--Q-A--AV--R-VES--ADL---A	ASRT	---VALKLEVRSG--A----
	Acidiphilium multivorum AIU301	326403463	--Q-G--EVS--S-RG-REEP-VAA	DSNT	--YVALRLA------A----
	Rhodopseudomonas palustris TIE	192292523	--Q--S-K-GDTDIPD-RSAKDVAA	DSTT	----AL-LS------A----
	Bradyrhizobium japonicum USDA	27381871	-AQ-L--R-GDEEITD-RKT-DVKP	GSTT	---VALKLM------A----
	Beijerinckia indica subsp. ind	182677454	--Q-GE-E-L--KIT--RAS-TV-P	ASMT	--Y-ALKLW------Q----
	Starkeya novella DSM 506	298293806	--Q-R--FV--EAVKD-RAEKDV-P	NSRT	--YVALKCFV-S---N----
	Bradyrhizobium sp. BTAi1	148240947	-AQ--E--VR-RAVRN-REEPNIAA	ASRT	--YVALRLM------A----
	Oligotropha carboxidovorans OM	209884211	--Q-L--R-GDRQVGY-RQTKDVSP	DSTI	--Y-ALKLT------A----
	Nitrobacter hamburgensis X14	92118736	--Q-H--K-GD-AVGD-RKTPDVKP	DSTT	--Y-ALKLT------A----
	Rhizobium etli CNPAF512	327188643	--A----PL--VELP--RETKDVSP	DSRT	--YVALKLYA-T---A----
β-Proteobacteria	Burkholderia xenovorans LB400	91778346	--Q----AVD-EAVKG-LEEDNVPA	GSRA	---VALR---N----AH---
	Ralstonia solanacearum CMR15	299069923	--Q-LE-A-A---V-G-LAEH-IPH	DSRT	---VAIK-E-A----A----
	Baumannia cicadellinicola str.	94676633	--Q----IVH-RKVPG-LEER-ANK	NSNT	---IS-RVD----Q-V----
	Delftia acidovorans SPH-1	160901031	--Q-L--ASQ---VP---QEK-VAA	GSQT	---VALRTE-C----A----
	Rhodoferax ferrireducens T118	89899926	--Q-A--S-G-A-VA--REET-V-P	HSHT	---VALRTE-S----A----
	Leptothrix cholodnii SP-6	171060061	--Q-R--NVD-KKVP--LDEVKVPT	GSHC	---VAIRTEVQ----A----
	Burkholderia multivorans CGD2M	221201170	--Q-H--V-K-VQVP--ATEP-VKP	DSQT	---VALK-E-E----A----

Figure S34

Partial sequence alignment of a conserved region in glucose-6-phosphate 1-dehydrogenase showing a 4 aa deletion that is uniquely present in all Xanthomonadales except *Pseudoxanthomonas spadix* BD-a59 and *Rhodanobacter* sp. 2APBS1 which has 3 aa insert.
