# Supplementary material for: Phylogenomics and Molecular Signatures for Species from the Plant Pathogen-Containing Order Xanthomonadales
Source: PLoS One. 2013 Feb 8;8(2):e55216. doi: 10.1371/journal.pone.0055216 (PMC3568101; doi:10.1371/journal.pone.0055216)
Supplement: Figure S35 — Partial sequence alignment of a conserved region of DNA ligase NAD dependent, showing a 57–65 aa insert that is commonly shared by all Xanthomonadales except Pseudoxanthomonas suwonensis and Rhodanobacter sp. 2APBS1. Both these species do not contain the insert of same length. This CSI was previously identified by [28] as all Xanthomonadales specific signature. (PDF) [file pone.0055216.s035.pdf]

DNA ligase signature

			461		583
Xanthomonadales	Xylella fastidiosa 9a5c	77747612	ELLVDAAVVHGVADLYHLSLDQLLRL	RLVTNAQTPTMLLREARDHVTGMRYQQLEEILRTVGVDLSGEGDVPKHWQIDVLRAQWPDFDWNH	KKIATKWAQNLIAAIDRSRQTTLERFLFALGM
	Xylella fastidiosa Ann-1	71899829	--------------------------	---E-----------------	--------------------------------
	Xylella fastidiosa Dixon	71275191	--------------------------	---E-----------------	--------------------------------
	Xanthomonas gardneri	325922521	-V---SG--Q------L-TV----Q-	--IST-ES-HAF-----E-LATGA-A---NTVVSI----A--RE--QT--A-L---GL-A----R	--------E---E--EK--D----------I
	Xanthomonas oryzae	188576356	-V---SG--Q------L-TV----Q-	-MIST-ES-HAF-----E-LASGA-A---AT-VGI----A--RE--QT--A-L---GL-T----R	--------E---E--ET--D----------I
	Xanthomonas axonopodis	346724509	-V---SGL-K------L--V----Q-	--IST-ES-HAF-----E-LASGA-A---TSVVGI----A--R---QT--A-L---GL-R----R	--------E---E--ET--D----------I
	Xanthomonas campestris	78047224	-V---SGL-K------L--V----Q-	--IST-ES-HAF-----E-LASGA-A---TSVVGI----A--R---QT--A-L---GL-R----R	--------E---E--ET--D----------I
	Xanthomonas perforans	325928155	-V---SGL-K------L--V----Q-	--IST-ES-HAF-----E-LASGA-A---TSVVGI----A--R---QT--A-L---GL-R----R	--------E---E--ET--D----------I
	Xanthomonas fuscans	294626370	-V---SGL-K------L--V----Q-	--IST-DS-HAF-----E-LASGA-A---ASVVGI----A--R---QT--A-L---GL-S----R	--------E---E--ET--D----------I
	Xanthomonas vesicatoria	325915467	-V---SGL-Q------L-TV----Q-	--IST-DS-HAF-----E-LATGA-A---ATMMRI----A--HEA-QT--A-L---GL-S----R	R-------E---E--ET--N----------I
	Pseudoxanthomonas spadix	357417248	-V----G--Q------R-D--L--Q-	----S----EGF--A--E-LASGA-ST--ATVARI CGKA-A--DT--AEL---GL-A----R	----S---------L-K-KH----------I
	Stenotrophomonas maltophilia	194366356	-T----GI-K------R--R----H-	K--LD-ED-SA-AAALKL-LPAEGSGAVLNAV -KLD-ND-G -RAQA- V-PAS-E--T	--------D-------A--AA----L-----I
	Stenotrophomonas sp. SKA14	254523079	-T----GI-KS-----R-NR----H-	K--LD-ED-SA-AAALKL-LPAEGSGAVLNAV -KLD-ND-G -RTQA- V-PAS-E--T	--------D-------A--AA----L-----I
	Xanthomonas albilineans	285018504	--EFGF-QSL----A-RVED-V-M	KAALD-ASSAD--QAVEESKGALVLDAAGYAA -AQQVPGWR HADFLR RHL AV-LG	G-L-----E--L-G--A--A---S-------I
	Rhodanobacter sp. 2APBS1	352085801	-A--ELDI--SP----G-GV-DFVEM	KRRIDERDG-TP ETVKA	G-V-----E--V-G-EA-KR---A-------I
	Pseudoxanthomonas suwonensis	319787332	-S-AEFGYLRD-S---R-T--D--EM	KRRADEREG-TP VAPAKSGVKS	G-V--R--D--L----T--N---A---Y---I
Other Bacteria	Escherichia coli	195938757	DQ--EKEY--TP---FK-TAGK-TG-		ERMGP-S---VVN-LEKAKE--FA---Y---I
	Citrobacter koseri	157144662	DQ--EKEY--TP---FR-TAGK-TG-		DRMGP-S---VVN-LEKAKE--FA---Y---I
	Klebsiella pneumoniae	364519394	DQ--EKEY--TP---FR-TAGK-TG-		DRMGP-S---VVN-LEKAKE--FA---Y---I
	Enterobacter aerogenes	336246576	DQ--EKEY--TP---FR--AGK-TG-		DRMGP-S---VVN-LEKAKE--FA---Y---I
	Cronobacter sakazakii	156933031	DQ--EKEY--TP---FR-TAGK-TG-		DRMGP-S----VN-LEKAKE--FA---Y---I
	Salmonella enterica	213618983	DQ--EREY--TP---FR-TAGK-TG-		DRMGP-S---VVN-LEKAKA--FA---Y---I
	Erwinia billingiae	300717845	DQ--EKEY--TP---FR-TAGK-TG-		DRMGP-S---VVNSLEKAKT---A---YS--I
	Photorhabdus luminescens	37525354	DQ---KEY-KTP---FR-TAGK-TG-		DRMGP-S---VVD-LEKAKK--FA---Y---I
	Pantoea vagans	308187689	DQ--EKEY-DTP---FR-TAGK-TG-		DRMGP-S---IVD-LEKAKS---A---Y---I
	Dickeya dadantii	307132386	DQ--EKEY-KTP---FR--AGI-TG-		DRMGP-S----VN-LNKAKS--FA---Y---I
	Yersinia pseudotuberculosis	153948204	-Q--EKQY-ENP---FQ-TAGK-TG-		DRMGP-S-------LEKAK---FA---Y---I
	Aliivibrio salmonicida	209695779	-Q---KEM-ETP---FK--AGV-TV-		DRMGP-S---VVN-LNKAKE---S---YS--I
	Providencia stuartii	183599716	DQ--EKEY-KNP----Q--AGI-TG-		DRMGP-S----VE-LNK-KS---A--IY---I
	Vibrio cholerae	153217073	-Q---REM-STP---FR-RAGE-TI-		ERMGP-S---V-D-LNKAK----PK--Y---I
	Proteus mirabilis	197285679	DQ--EKEY-KTP---FR-DEKI-SG-		ERMGE-S-KK-LN-LEKAKS--FA--IY---I
	Acidovorax delafieldii	241764431	DQ----N-IRTLP---R-G-TS-IA-		ERM-E-S---VL--LEK-K----P-------I
	Burkholderiales bacterium	303258051	-Q-IEHDL-TT---I-R-TV-K-VG-		DRMGQ-S-EK-V-S-EA-KK-------Y-ISI
	Nitrosomonas eutropha	114330949	DQ-I-REL--TP----K-NI-T-AS-		ERM-E-S-R--VT--EH-KK---P--IY---I
	Thiocystis violascens	350556847	-Q---LDW-REP----R-TPE--A--		ERMGE-S-A--L--LE--KE-SFA--I----I
	Desulfobacca acetoxidans	328954073	-Q-I-QGL-QD-----R-QASD--P-		ERF-E-S---IVQ--AG-KKPS-G--IY---I
	Methylovorus glucosetrophus	253999028	DQ---G-HI-TL--I-R-D-AT-SS-		DRM-E-S-K-I-K-L-A-KA---A--IY---I
	Pseudomonas psychrotolerans	359783270	-Q---R-L-KSP----T-TYE-VVE-		EGF-EVSSR--L---EA--RPA-A--I----I
	Oceanospirillum sp. MED92	89094416	-M---LDM--NP----A-QQTE-A--		ERMGP-S-E--VN--QA--N-E-P---Y---I
	Haemophilus influenzae	260581847	-Q---RELI-TP---FK-D-TT-T--		ERMGV-S-E-ALNSLEKAKS---A--I----I
	Pseudomonas aeruginosa	152984776	-Q---RGL-ASP----T-TY--VFE-		EGF-ELSTN--L---AD--TPS-A--I----I
	Aggregatibacter aphrophilus	347813461	-Q---REL--TP---FK-D-AT-T--		ERMGS-S-E-ALVSLEKAKH---A--I----I
	Salmonella enterica	213865477	DQ--EREY--TP---FR-TAGK-TG-		DRMGP-S---VVN-LEKAKA--FA---Y---I
	Francisella novicida	208779663	-Q--A-NLIKYP--I-K-NF---TG-		ERMGA-SS--VLDS-KK-KTPS-A--I--I-I
	Shewanella baltica	217972882	-Q-I-KEL-QSP---FK-TASMMTM-		DRMGM-S-T--A---EAAKT---A---Y---I
	Enterobacter cloacae	296104078	DQ--EKEY--TP---FK-TAGK-TG-		DRMGP-S---VVN-LEAAKE--FA---Y---I
	Geobacter metallireducens	78223924	-Q-LRLGL-GN-----T-TR-DFMKF		DRMGE-L-E--LN--EA-KKRE-S--I----I
	Bdellovibrio bacteriovorus	42521704	-T---NKLLTRFS-F-R-TKE-I-S-		ERQGD-S-D-I-KS-EN-KNP--A--I----I
	Gemmatimonas aurantiaca	226226196	QQ-L--GL--D----FDITV---TS-		ERF-K-S-E--V---AAAK-QP-S-LM-G--I
	Capnocytophaga sputigena	213962174	-Q-FR-GII-TY----EVTVEK--P-		ERM-Q-S-E-IVKG-QK-KEIPF--V-----I
	Enterococcus italicus	315641089	-QFFNHGF-ED-----R-T-----TI		D-VKE-S-T-MLQ--EA-K-NS--KL--G--I
	Bacillus sp. B14905	126649600	-Q-LR-DLI-D-S-----TIE--VE-		ERMGE-S-T--VN--QA-KENSM--L-IG--I

Figure S35

Partial sequence alignment of a conserved region of DNA ligase NAD dependent, showing a 57-65 aa insert that is commonly shared by all Xanthomonadales except *Pseudoxanthomonas suwonensis* and *Rhodanobacter* sp. 2APBS1. Both these species do not contain the insert of same length. This signature was previously identified by (Cutino-Jimenez et al., 2010) and was described as all Xanthomonadales specific Signature.
