# Supplementary material for: Phylogenomics and Molecular Signatures for Species from the Plant Pathogen-Containing Order Xanthomonadales
Source: PLoS One. 2013 Feb 8;8(2):e55216. doi: 10.1371/journal.pone.0055216 (PMC3568101; doi:10.1371/journal.pone.0055216)
Supplement: Figure S36 — A Neighbor-joining tree based upon carbamoyl phosphate synthase large subunit sequence. The Tree is showing the distinct branching of Xanthomonadales from various β-Proteobacteria with and without insert. (PDF) [file pone.0055216.s036.pdf]

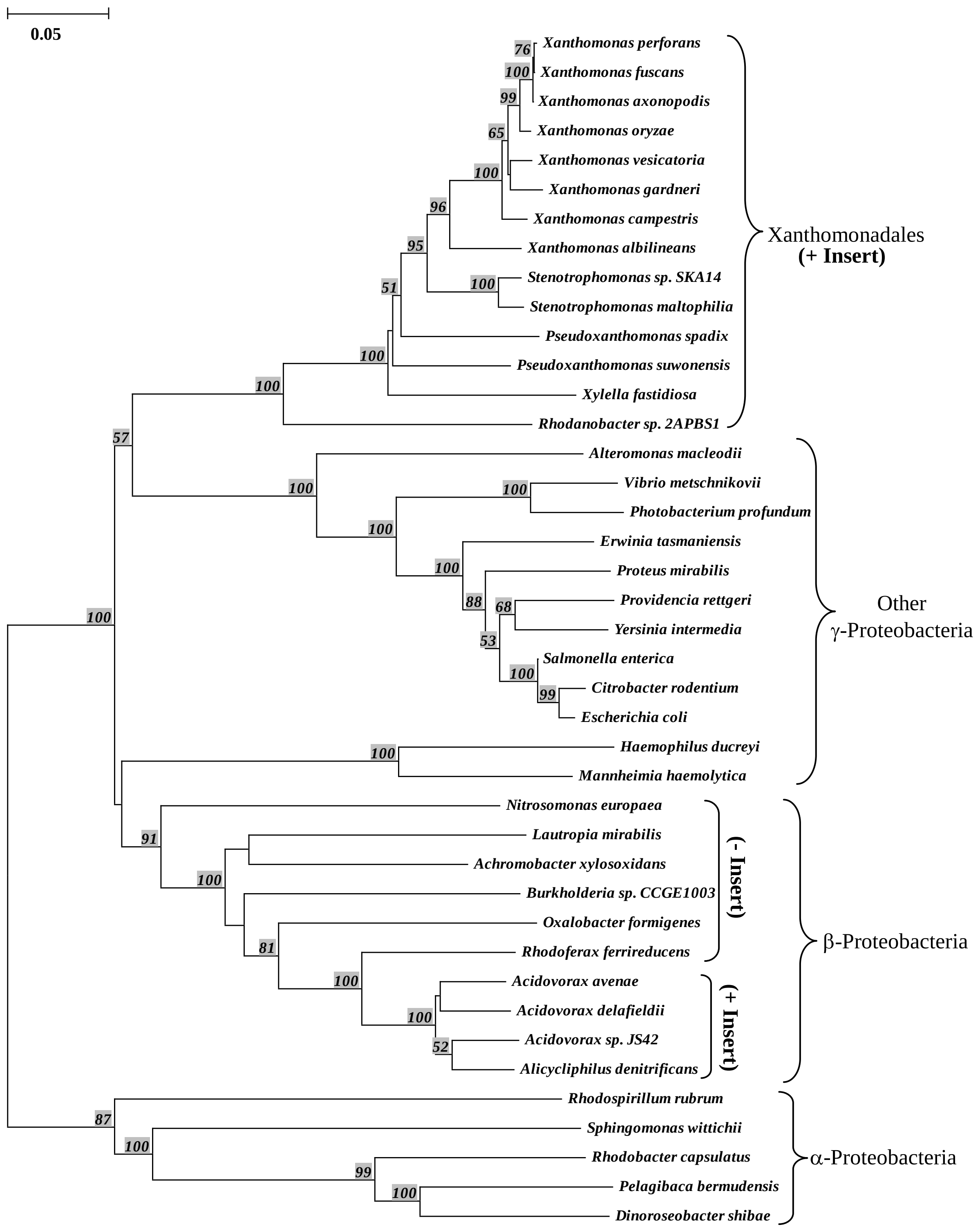

Figure S36

A Neighbor-joining (NJ) tree based upon carbamoyl phosphate synthase large subunit sequence. The numbers on the nodes indicate the bootstrap values. The Tree is showing the Xanthomonadales and various β-Proteobacteria with and without insert. Species representing α-Proteobacteria and some other Gammaproteobacteria are also shown in tree. The information for the respective CSI is shown in Fig. 8
