# Supplementary material for: Phylogenomics and Molecular Signatures for Species from the Plant Pathogen-Containing Order Xanthomonadales
Source: PLoS One. 2013 Feb 8;8(2):e55216. doi: 10.1371/journal.pone.0055216 (PMC3568101; doi:10.1371/journal.pone.0055216)
Supplement: Figure S37 — Partial sequence alignment of a conserved region of Hypothetical protein XOO1065, showing a 1 aa deletion that is present in Xanthomonadales. The deletion has also been found to be shared by few species from β-Proteobacteria but not in all of them. (PDF) [file pone.0055216.s037.pdf]

Group	Species	ID	36 … 77
Xanthomonadales	Xanthomonas oryzae	58580688	GGGMVNVTLTGAKECRKVRIDPSILSDQ EMAEDLIAAAFNDA
	Xanthomonas axonopodis	21241865	-----S---------------------- --------------
	Xanthomonas campestris	78046684	-----S---------------------- --------------
	Xanthomonas albilineans	285017409	---------------------------- --------------
	Stenotrophomonas maltophilia	190573046	-----S-----------------LT--P --L-----------
	Xylella fastidiosa	15838407	-----S-I-S-T------------N-- --I-----------
Other γ-Proteobacteria	Actinobacillus minor	240948278	-A-L-K--IN--HN--RIE----LME-D K --V---V-------
	Aeromonas hydrophila	117620231	-A---K--MA-SHSV-RIE----LME-D K --L---V---V---
	Aggregatibacter aphrophilus	251793009	-A-L-KI-VN--HN--R-E----LME-D K --L-------Y---
	Alkalilimnicola ehrlichii	114320665	-----A--IN-KH-A-R-S----LFE-D R --V---V-------
	Arsenophonus nasoniae	284006439	-A-L-K--IN--HN--R-E----LMA-D K D-L-----------
	Citrobacter koseri	157146902	-A-L-K--IN--HN--R-E----L-E-D K --L---V-------
	Colwellia psychrerythraea	71281215	-A---K--M--SHSV---EL-D-LME-D K D-I---L---V---
	Cronobacter sakazakii	156934950	-A-L-K--IN--HN--R-E----L-E-D K --L---V-------
	Dickeya dadantii	307129959	-A-L-K--IN--HN--R-E----LME-D K --L-----------
	Edwardsiella ictaluri	238919041	-A-L-K--IN--HN--R-E----LME-D K --L---V-------
	Enterobacter cancerogenus	261341081	-A-L-K--IN--HN--R-E----L-E-D K --L---V-------
	Erwinia tasmaniensis	188534598	-A-L-K--IN--HS--R-EV---L-E-D K D-L---V-------
	Escherichia coli	26246486	-A-L-K--IN--HN--R-E----L-E-D K --L---V-------
	Haemophilus influenzae	68249042	-A-L-KI-IN--HN--RID----LME-D K --L-----------
	Klebsiella pneumoniae	206578120	-A-L-K--IN--HN--R-E----L-E-D K --L---V-------
	Pantoea ananatis	291616583	-A---K--IN--HN--R-EV---L-E-D K D-L-----------
	Pasteurella dagmatis	260914408	-A-L-KI-IN--HN--RIE----LME-D K --L-----------
	Pectobacterium carotovorum	227328731	-A-L-KI-IN--HN--R-E----LME-D K --L-----------
	Photorhabdus luminescens	37527700	-A-L-K--IN--HN--R-E----LME-D K --L-----------
	Proteus mirabilis	197284052	-A-L-KI-IN--HN--R-E----L-E-D K --L-----------
	Providencia stuartii	183600058	-A-L-K--IN-SHN--R-E----L-E-D K --L-----------
	Salmonella enterica	56414360	-A-L-K--IN--HN--R-E----L-E-D K --L---V-------
	Shewanella sediminis	157375986	-A-L-K--M--SHSV---D----L-E-D K --L-------C---
	Sodalis glossinidius	85058668	-A-L-K--IN--HN--R-EV---L-E-D K D-L-----------
	Vibrio cholera	255745472	-A-L-K--V--SHSV-R-N--E-LME-D K --L-----------
	Xenorhabdus nematophila	300721961	-A-L-KI-IN--HN--R-E----LMD-D K D-L-----------
β-Proteobacteria	Achromobacter xylosoxidans	317406001	---L-K--M-CRHDVKR-E----L-A-D K D-L---V-------
	Bordetella parapertussis	33595894	---L-K--M-CRHDVKR-A----L-GED K D-L---V-------
	Thiomonas intermedia	296136311	-A-L-K--M-CKYGVKR-N----LVAED R D-L---V-------
	Pusillimonas sp. T7-7	332283344	---L-K-SM-CRHDVKR-T----L-A-D K D-L---V-------
	Burkholderia rhizoxinica	312796076	-A-L-K--M-CKN-V-R-T----L-A-D K D-L---V-------
	Bordetella pertussis Tohama	33592642	---L-K--M-CRHDVKR-A--A-L-GED K D-L---V-------
	Achromobacter piechaudii	293607023	---L-K--M-CRHDVKR-V----L-G-D K D-L---V-------
	Bordetella petrii	163857865	---L-K-SMSCRHDVKR-A----L-A-D K D-L---V-------
	Dechloromonas aromatica	71906446	-A---K-LM-C-H-V-R-N----VMD-R --L-------L---
	Thauera sp. MZ1T	217969821	-A---K-QM-CKYDV-R-S--E-VMD-R --L---V---L---
	Sideroxydans lithotrophicus	291613733	-S---K-IM-C-H-V-R-NL-Q-V-D-K --L----V--L---
	Methyloversatilis universalis	334130680	-A---K-VM-CKHDV-R-S----VMD-R --L---L---V---
	Laribacter hongkongensis	226939892	-A---KI-MSCNHDVKR-T--DGVMD-K --L-------I---
	Candidatus Accumulibacter	257091957	-A---K-LM-CGHDV-R-A----VMD-R --L---V---L---
	Aromatoleum aromaticum	56476073	-A---K-LM-CKYDV-R-T--D-VMD-K --L---L---V---

Figure S37

Partial sequence alignment of a conserved region of Hypothetical protein XOO1065 showing a 1 aa deletion that is present in all Xanthomonadales. The deletion has also been found to be shared by few species from β-Proteobacteria but not in all of them.
