# Supplementary material for: Phylogenomics and Molecular Signatures for Species from the Plant Pathogen-Containing Order Xanthomonadales
Source: PLoS One. 2013 Feb 8;8(2):e55216. doi: 10.1371/journal.pone.0055216 (PMC3568101; doi:10.1371/journal.pone.0055216)
Supplement: Figure S38 — Partial sequence alignment of a conserved region of orotate phosphoribosyltransferase, showing a 1 aa deletion that is present in all Xanthomonadales. The deletion has also been found to be shared by species from β-Proteobacteria. (PDF) [file pone.0055216.s038.pdf]

Group	Species	ID	69		107
Xanthomonadales	Stenotrophomonas maltophilia	194364072	GPAYKGIPLATAMACELAQRGRELPL		SFNRKEAKAHGEG
	Stenotrophomonas sp. SKA14	254524635	----------------------D---		--------D----
	Xanthomonas albilineans	285017194	------------L---Y-R---D-A-		A-------I----
	Xanthomonas fuscans	294666112	------------L--TY-G---D---		A-------D----
	Xanthomonas oryzae	166713818	------------L--AY-G---D---		A-------D----
	Xanthomonas campestris	289666317	------------L--AY-G---D---		A-------D----
	Xanthomonas axonopodis	21244622	------------L--AY-G---D---		A-------D----
	Xanthomonas oryzae	188578885	------------L--AY-G---D---		A-------D----
	Xylella fastidiosa	28198058	------------L---Y---E-D-L-		T-----V-N----
Other γ-Proteobacteria	Acinetobacter calcoaceticus	262280500	--------FVA-T-VA-S-VHNKSVP	W	G-------D----
	Actinobacillus minor	257465331	--------I--TV-VA--NQFNVDKP	C	C-------D----
	Actinobacillus succinogenes	152978105	--------IG-TVSVA-FN-FGLDKP	V	C-------D----
	Aeromonas hydrophila	117618078	--------I-S-T-VQ-VEQHDVDVP	W	C-------D----
	Aggregatibacter aphrophilus	251793340	--------I---VSVA-FNQFNVDKP	V	C-------D----
	Alcanivorax borkumensis	110833068	---------VA-VSVA--EHHGRDYP	W	A-------D----
	Alteromonas macleodii	239994378	--------I--TT-VA--DSHDLDVP	Y	C-------T----
	Cardiobacterium hominis	258545111	---------VA-V-VI-HAEHGLSLP	W	T-------D----
	Chromohalobacter salexigens	92115319	-----------TT-VA--DHHDRDLP	F	A------------
	Citrobacter koseri	157149260	--------I--TT-VA--EHHDKDLP	Y	C-------D----
	Congregibacter litoralis	88705454	-----------TT-VA--DHHGRDVP	F	AY------S----
	Dickeya zeae	251791522	--------I--TT-VA--EHHDRDVP	Y	C-------D----
	Edwardsiella ictaluri	238918031	--------I--TT-VA--EHHDRDMP	Y	C-------D----
	Enterobacter cloacae	295095227	--------I--TT-VA--EHHDRDVP	Y	C-------T----
	Erwinia pyrifoliae	259906755	--------I--TTVVA--DHHDRDVP	Y	C-------D----
	Escherichia albertii	170766648	--------I--TT-VA--EHHDRDLP	Y	C-------D----
	Escherichia coli	15804183	--------I--TT-VA--EHHDLDLP	Y	C-------D----
	Grimontia hollisae	262273501	--------I--TT-VA--EHHNVDTP	Y	C-------D----
	Haemophilus ducreyi	33152772	--------I--TV-VA--NQFAIDKP	C	C-------T----
	Haemophilus influenzae	301168925	--------IG-SVSVA-FN-YGIDKP	V	C-----V-D----
	Klebsiella pneumoniae	206579381	--------I--TT-VA--EHHDRDLP	Y	C-------T----
	Methylococcus capsulatus	53803127	---------VA-T-IA--RVSG-EIP	Y	A-------D----
	Nitrococcus mobilis	88813080	----------C-T-IA--EHHGRDVP	Y	A-D---T-T----
	Oceanobacter sp. RED65	94502049	----------ATT-MA-S-EFNRDTP	Y	T-----K-Y----
	Oceanospirillum	89093848	----------ATT-VA-SDQHG-DVP	Y	V-------D----
	Pantoea ananatis	291619461	--------I--TT-VA--DHHDRDVP	Y	C-------D----
	Pectobacterium wasabiae	261823587	--------I--TA-VA--EHHDRDLP	Y	C-------D----
	Photobacterium damselae	269103913	--------I--TT-VA--DQHDIDTP	Y	C-------D----
	Proteus penneri	226326903	--------I--TT-VA-VEHHDIDIP	Y	C-------D----
	Providencia stuartii	183597212	--------I--TT-VA--EHHDVDMP	Y	C-------D----
	Pseudomonas stutzeri	146280861	---------GA-T-IA--EQHQRDLP	W	C-------D----
	Salmonella enterica	161505732	--------I--TT-VA--EHHDKDLP	Y	C-------D----
	Serratia odorifera	270265233	--------I--TT-VA--EHHERDVP	Y	C-------T----
	Shigella dysenteriae	82779133	--------I--TT-VA--EHHDLDLP	Y	C-------D----
	Sodalis glossinidius	85060190	--------I--TT-VA--EQHQRDVP	Y	C-------D----
	Tolumonas auensis	237806962	--------I-S-T-VQ--ELHDQDVP	W	C-------D----
	Xenorhabdus bovienii	290477305	--------I--TT-VA--E-HDIDMP	Y	C-------D----
	Yersinia aldovae	238760456	--------I--TT-VA--EHHERDVP	Y	C-------D----
β-Proteobacteria	Ralstonia pickettii	171363652	-------T--S-T-VA--GM--DVGF		AY------D----
	Cupriavidus taiwanensis	188590963	-------T--S-T-VA--GM--DVGF		AY------D----
	Sutterella wadsworthensis	319940716	----------ASV-MN-SRM-CDV-W		A-----I-D----
	Variovorax paradoxus	319790981	---------GATV-A---R---NY-F		AY-----------
	Polaromonas naphthalenivorans	121603085	----------A-V-I---RL--N--F		AY------D----
	Candidatus Accumulibacter	257092321	---------VA-I-VA--RA--NF-F		--------D----
	Methylibium petroleiphilum	124265281	-------T--A-V-I---RL--N--Y		AY----T-D----

Figure S38

Partial sequence alignment of a conserved region of Orotate phosphoribosyltransferase showing a 1 aa deletion that is present in all Xanthomonadales. The deletion has also been found to be shared by species from β-Proteobacteria.
