# Supplementary material for: Phylogenomics and Molecular Signatures for Species from the Plant Pathogen-Containing Order Xanthomonadales
Source: PLoS One. 2013 Feb 8;8(2):e55216. doi: 10.1371/journal.pone.0055216 (PMC3568101; doi:10.1371/journal.pone.0055216)
Supplement: Figure S39 — Partial sequence alignment of a conserved region of Putative ribonuclease HII, showing a 1 aa insert that is present in Xanthomonadales. The CSI has also been found to be shared by few species from β-Proteobacteria but is not present in all. (PDF) [file pone.0055216.s039.pdf]

Group	Species	Accession	27		80
Xanthomonadales	Stenotrophomonas maltophilia	190573489	AGVDEAGRGPLAGPVAVAAVVFDPARP	R	INGLDDSKQLTAARREQLHDRIIERA
	Stenotrophomonas sp. SKA14	254523247	------------------------S--	-	--------------------------
	Xanthomonas campestris	289670236	------------------------SK-	-	------------E-----YA--VD--
	Xanthomonas oryzae	58581586	------------------------NK-	-	------------E-----YA--VD--
	Xanthomonas albilineans	285018804	------------------------NQ-	-	----------------T-YP------
	Xanthomonas fuscans	294625960	------------------------GK-	-	------------E-----YA--VD--
	Xanthomonas axonopodis	21242160	------------------------GK-	-	------------E-----YA--VD--
	Xylella fastidiosa	15837643	---------------V--------SQ-	-	----N-----SP-C--R-YAH-V---
Other γ-Proteobacteria	Acinetobacter baumannii	184157471	-----------V-S-VA---IL--NN-		-L--N---K--EKK--K-FIE-Q-K-
	Allochromatium vinosum	288941772	---------------CA---IL-----		-A-IG---K-SP----R-EPL-RDQ-
	Azotobacter vinelandii	226945927	-----V-----C-A-VT---IL--E--		-L--N---K-SE----A-SEE-R-K-
	Cellvibrio japonicus	192358895	--C--V-------D-VA---IL--GQS		-I------K--EKK----F-E-RAK-
	Citrobacter koseri	157147385	-----V-----V-A-VT---IL-----		-V--N---K-SEK--LA-Y-E-K-K-
	Coxiella burnetii	212212281	---------------IT---ILN-EII		-E--A---K-SLKK--E-YEK--TNC
	Dickeya dadantii	242240382	-----V-----V-A-VT---IL---K-		-A--A-----SEK--RV-Y-E-R-K-
	Edwardsiella tarda	294635136	-----V-----V-A-VT---IL-----		-R--A---K-SEK--NA-Y-E-V-K-
	Enterobacter cancerogenus	261338819	-----V-----V-A-VT---IL-----		-V--N---K-SEK--LA-F-E-Q-K-
	Erwinia billingiae	300715410	-----V-----V-A-VT---IL-----		-V--A---K-SEK--LA-Y-E-V-K-
	Escherichia coli	297517117	-----V-----V-A-VT---IL-----		-A--N---K-SEK--LA-CEE-K-K-
	Idiomarina baltica	85711419	C---------I----VA---IL--EQ-		-E--N---K-S-KK--R-SEE-KAK-
	Kangiella koreensis	256823113	-----V---------VA---IL---N-		-V--A---K--EK---A-SIE-K-K-
	Klebsiella pneumoniae	206579509	-----V-----V-A-VT---IL---K-		-V--N---K-SEK--LA-C-E-K-K-
	Marinobacter algicola	149374423	-----V-----V-A-VT---ILN-E--		-P--A---K--DK---A-YEQ-----
	Oceanospirillum sp. MED92	89092102	-----V-----V-N-VA---IL--NK-		-E--A---K---K---A-YHE-KDK-
	Pantoea sp. aB	304396658	-----V-----V-A-VT---IL---N-		-T--A---K-SEK--LA-Y-E-K-K-
	Pectobacterium atrosepticum	50119985	-----V-----V-A-VT---IL--T--		-V--A-----SEK--LS-Y-E-K-K-
	Photorhabdus asymbiotica	253988139	-----V-----V-A-VT---IL--S--		-A--A---K-SEK---E-YLE-T-K-
	Proteus mirabilis	197286117	-----V-----V-A-VT---IL---N-		-Q--M---K--EKK-NA-Y-E-K-K-
	Providencia alcalifaciens	212710383	-----V-----V-A-VT---IL--NN-		-E--A---K-SEKK--K-F-E-K-K-
	Pseudomonas aeruginosa	107103156	-----V-----C---VT---IL--S--		-L--N---K-SE----A-FEE-R-K-
	Reinekea sp. MED297	88798271	-----V-------K-VT---IL-----		-D--N---K-SP---LA-SQH-R-N-
	Rickettsiella grylli	160872277	---------------FA---IL-NKKK		MT--K-------KS--YFAKK-Q-K-
	Salmonella enterica	161504651	-----V-----V-A-VT---IL-----		-V--N---K-SEK--LA-Y-E-K-K-
	Serratia odorifera	270264806	-----V-----V-A-VT---IL-----		-I--A---K-SEK--LA-Y-E-V-K-
	Shewanella sediminis	157376279	-----V-----V-N-VT---IL--TK-		-T--N---K-SEKK--A-FTE-H-K-
	Shigella flexneri	24111618	-----V-----V-A-VT---IL-----		-A--N---K-SEK--LA-YEE-K-K-
	Sodalis glossinidius	85059907	-----V-----V-A-VT---IL-----		VL--A---K-SEK---A-YEE-TRY-
	Xenorhabdus bovienii	290473671	-----V-----V-A-VT---IL---K-		-T--M---K--EK---A-YLE-K-K-
	Yersinia pestis	22126997	-----V-----V-A-VT---IL--N--		-V--A---K-SEK--LS-Y-E-T-K-
β-Proteobacteria	Burkholderia xenovorans LB400	91784105	C--------------VA---I----K-	M	-R------V---KK-DE-Y-K-VD--
	Burkholderia phytofirmans PsJN	187924419	C--------------VA---I---SK-	M	-R------V---KK-DE-Y-K-VD--
	Burkholderia phymatum STM815	186476082	C--------------VA---IL---K-	-	-R--T---V---KK-DE-YEK-----
	Ralstonia eutropha JMP134	73541556	C--------------YA--------K-	M	LR--A---V---SK--A-Y-K-C---
	Burkholderia graminis C4D1M	170692156	C--------------VA---I---SK-	M	-R------V---KK-DE-YEK-VD--
	Burkholderia rhizoxinica HKI 4	312796258	C--------------VA---IL---A-	-	-R------A-SP-T--K-F---V--S
	Methylibium petroleiphilum PM1	124262697	---------------VA---IL--S--		-D--N-----S-K--DR-F-L-----
	Cupriavidus necator N-1	338166264	C--------------YA----L--K--		-R--A---I----K----Y-K-C---
	Ralstonia eutropha H16	113868020	C--------------YA---IL--K--		-R--A---I----K----YEK-C---
	Burkholderia cenocepacia J2315	206560439	C--------------VA---IL-----		-D------V-S-KK-DL-Y-L-VA-S
	Variovorax paradoxus EPS	319793969	---------------VA---IL-DT--		-R--A---T---LQ--R---Q-LAK-
	Ralstonia solanacearum CMR15	299067473	C--------------TAG---LN-RK-		-Q--A---V---KK--A-Y-E-V-K-
	Variovorax paradoxus S110	239815594	---------------VA---IL-DQ--		-R--A---T---LQ--R-N-Q-LAK-
	Comamonas testosteroni KF-1	221066099	---------------VA---IL-DMK-		-A--N---K---N---V-Y-E-RAK-
	Acidovorax avenae subsp. avena	326316587	---------------VA---IL-ELQ-		-D--A---T-------A-F-E-RAK-
	Acidovorax citrulli AAC00-1	120610517	---------------VA---IL-ELQ-		-E--A---T-------A-F-E-RAK-
	Aromatoleum aromaticum EbN1	56478865	C------------S-VA---IL-----		-D--N---K--ERA--R-APL-R---
	Sideroxydans lithotrophicus ES	291614100	C--------------SA---IL-KS--		-E--A---K-SERQ-D--APL-R---
α-Proteobacteria	Methylocella silvestris BL2	217977760	----------------A---IL--DNL		PR--N------REE--K-YES-MK--
	Bradyrhizobium japonicum USDA	27377632	--C------------VA---IL--D-I		PR-I----R---EQ--K-F-K-CAT-
	Agrobacterium radiobacter K84	222085047	--A------------VA---IL--E-I		PQ--N-----S-----A-FAE-LAT-
	Hoeflea phototrophica DFL-43	163761011	--L------------VA---IL----I		PE--N---A-SRKK--T-F-Q--AT-
	Candidatus Hamiltonella defens	238897797	-----V---------VT---IL--KN-		-I--A---T-SKKK-MA-YEE-THK-
	Bradyrhizobiaceae bacterium SG	338973306	--C------------VA---IL--K-I		PK-I----R-SKE-----FED-CAT-
	Oceanibulbus indolifex HEL-45	163745410	-----V---------TA-----TEGQI		PE--N---K---RA--R-Y-E-MSM-
	Sulfitobacter sp. NAS-14.1	83855284	-----V---------TA----L--D-I		PE--N---K--KPA--R-YEELLQV-
	Rhodobacterales bacterium HTCC	126724759	-----V---------TA---IL--DNI		PE--N---K-S-KK-DL-F-Q-MNV-

Figure S39

Partial sequence alignment of a conserved region of Putative ribonuclease HII showing a 1 aa insert that is present in all Xanthomonadales. The CSI has also been found to be shared by few species from β-Proteobacteria but is not present in all.
