# Supplementary material for: Phylogenomics and Molecular Signatures for Species from the Plant Pathogen-Containing Order Xanthomonadales
Source: PLoS One. 2013 Feb 8;8(2):e55216. doi: 10.1371/journal.pone.0055216 (PMC3568101; doi:10.1371/journal.pone.0055216)
Supplement: Figure S40 — Partial sequence alignment of a conserved region of glycyl-tRNA synthetase subunit beta, showing a 2 aa insert that is present in Xanthomonadales. The insert has also been found to be shared by some β-Proteobacteria. (PDF) [file pone.0055216.s040.pdf]

			12		58
Xanthomonadales	Xanthomonas axonopodis	21244927	GTEELPVKALPGLAQALFDGVLSGLEKRGIAVA	RG	DAKPLSTPRRLA
	Xanthomonas fuscans	294624286	-------------R-F-----------V--T	--	------------
	Xanthomonas campestris	78049866	------------E--F-----------V--T	--	------------
	Xanthomonas oryzae	166714071	----------D----F---------------T	--	A-----------
	Xanthomonas albilineans	285016900	--------------------IA--D------E	--	------------
	Stenotrophomonas sp. SKA14	254522505	---------------F----VD--R---V-LD	L-	--R---------
	Stenotrophomonas maltophilia	190576418	---------------F----VE--R---V-LE	L-	--R---------
	Xylella fastidiosa	15838553	-------N---N-SR-F-E---A-------V-D	H-	E--S--------
Other γ-Proteobacteria	Actinobacillus minor	257465118	------P---KK--T-FAEN-EAE-NQA-LSFD		KVEWFAA-----
	Aeromonas salmonicida	145301092	--A---P---RS--E-FA-NFKAE-T-ADL-FG		-IEWFAS-----
	Aggregatibacter aphrophilus	251793743	------P---KT--TSFA-N-EAE-NQA-LTFD		KIEWFAA-----
	Alcanivorax borkumensis	110832869	------P---AS-SA--T-EFVRQ-DEA-LNHG		AVERFAA-----
	Azotobacter vinelandii	226942180	-S----P---KS--T-FLA-IEK--AAA-LEYR		V-RFYAA-----
	Cardiobacterium hominis	258545073	-V----T--VTT--D-GR-LWAQA-ANA-LTHG		-IEA-A------
	Cellvibrio japonicus	192359481	------P-N-KN-MN-FAESIQAD-TALELSFT		SV-AYAA-----
	Citrobacter koseri	157149175	------P---RS--ESFAAN-TAE-DNA-L-HG		NVEWFAA-----
	Colwellia psychrerythraea	71281166	------P-S-KT--T-FY-NIKGQ-DSHNLSYS		-I-WFA----F-
	Cronobacter turicensis	260599858	------P---RS--ESFAANFTAE-DAA-LTHG		VVSWFAA-----
	Dickeya dadantii	242237521	------P---RN--ESFAANFTAE-DTA-LGYQ		SVNWFAA-----
	Edwardsiella ictaluri	238917996	------P---RS----FA-NFRTE-DNA-L-HG		-IEWFAA-----
	Enhydrobacter aerosaccus	257453498	------P-N-KT-RD--NNH-TQS-KDAN-RFD		TI-SFAA-----
	Erwinia amylovora	292490092	------P---RS--ESFAANLTAE-DAA-L-HG		EVSWFAA-----
	Escherichia coli	193068544	------P---RS--EFFAANFTAE-DNA-L-HG		TVQWFAA-----
	Haemophilus influenzae	145640588	------P---KT--TSFA-N-EAE-IQA-LSFD		KIEWFAA-----
	Hahella chejuensis	83642934	-A----P---RQ--E-FAQ-IRQR---AQLSFS		ELTWYAA-----
	Idiomarina baltica	85711017	------P---KQ-SESFKH-IEE--N-AEL-FG		EI-A-A------
	Klebsiella pneumoniae	206579067	------P---RS--ESFAAN-TAE-DNA-L-HG		KVEWFAA-----
	Mannheimia haemolytica	254362729	------P---KK--T-FAEN-ENE-NQA-LSFE		KVEWFAA-----
	Marinobacter sp. ELB17	126668181	------P---KQ-SD-FTQ-IV---QAA--EFG		AVE-FAA-----
	Nitrosococcus watsoni	300115242	------P---RK-SES-LQELGR--QAA-LIY-		-L-GYGA-----
	Oceanobacter sp. RED65	94502179	------PT--KS-RD-FQK-IETF-QAK-LSF-		NSEAFAA-----
	Pasteurella dagmatis	260914511	------P---KK--T-FAEN-ELE-NQA-LTFD		KVEWFAA-----
	Photobacterium profundum	54307292	------P---RT--E-FSSNFEAE-KTAALVHQ		GIEWFA------
	Proteus mirabilis	197286684	------P---RS--ESFATHFTAE-DNAN-THG		-VSWFAA-----
	Providencia stuartii	183597049	------P---RS--ESFAANFTAE-DSAD--HG		EVSWFAA-----
	Pseudomonas aeruginosa	296386480	------P---NS-GE-FLS-IEK--KAA-LGY-		A-RFYAA-----
	Psychromonas ingrahamii	119947319	------P-S-RQ--ESFASN-EAE-N-AELSFD		SVRW-AS-----
	Salmonella enterica	56415548	------P---RS--ESFAANFTAE-DNA-L-HG		NVEWFAA-----
	Shigella boydii	187731977	------P---RS--ESFAANFTVE-DNA-L-HG		TVQWFAA----T
	Tolumonas auensis	237806784	------P---RK----FA-NFTAE-D-A-L-HQ		GVQWFAA-----
	Vibrio furnissii	260771056	------P-Q-RT--E-FAANFTAE-NNA---HD		GVTWYA------
	Yersinia ruckeri	238754024	------P---RS--ESFAANITAE-DSANLSHG		-VSWFAA-----
β-Proteobacteria	Ralstonia eutropha H16	113866550	F-----P---AR-GD-FAQ-LFA--GE-DLLEP	GA	TVT-FA------
	Cupriavidus necator N-1	338164853	F-----P---GR-GD-FAQ-LFA--GE-DLLEP	GA	TVT-FA------
	Ralstonia eutropha JMP134	73540213	F-----P---AR-GD-FAQ-LFA--RE-DLLE-	DA	AVT-FAS-----
	Bordetella pertussis	33591304	F-----P---QK-G--FAE--RAT-ARHHLLAD	GC	AVE-FA------
α-Proteobacteria	Starkeya novella	298293954	FS--I-ARMQA-A-EN-RKL-TDA-VE--LLYE		G--AFV------
	Rhodobacterales bacterium	254465195	FS--I-ARMQARA-ED-KKR-TD--VEA-LTY-		G-AA-------T
	Oceanicola granulosus	89070594	LS--I-ARMQAKASDD-KKM-TD--VEA-LTY-		S-AAFA-----V
	Roseovarius nubinhibens	83952444	FS--I-ARMQAKA-ED-RQR-TD--VEA-LTY-		G-ASF------T

Figure S40

Partial sequence alignment of a conserved region of glycyl-tRNA synthetase subunit beta showing a 2 aa insert that is present in all Xanthomonadales. The insert has also been found to be shared by species from β-Proteobacteria.
