# Supplementary material for: Phylogenomics and Molecular Signatures for Species from the Plant Pathogen-Containing Order Xanthomonadales
Source: PLoS One. 2013 Feb 8;8(2):e55216. doi: 10.1371/journal.pone.0055216 (PMC3568101; doi:10.1371/journal.pone.0055216)
Supplement: Figure S41 — Partial sequence alignment of a conserved region of the septum-site determining protein MinD, showing a 1 aa deletion that is present in Xanthomonadales. This CSI is also present in some species from β-Proteobacteria. (PDF) [file pone.0055216.s041.pdf]

Group	Species	ID	144		194
Xanthomonadales	Stenotrophomonas maltophilia	194364873	NPEVSSVRDSDRIIGLLDSKTHKAESG		QDVPAFLLLTRYTPVRVESGEMLS
	Stenotrophomonas sp. SKA14	254522605	---------------------------		------------------------
	Xanthomonas albilineans	285017689	-------------L-----------N-		KSL---------S-A---G-----
	Xanthomonas campestris	21230582	---------------------R---E-		KN----------S-G---G-----
	Xanthomonas oryzae	84624994	---------------------R---E-		KA----------S-G---G-----
	Xanthomonas axonopodis	21241979	---------------------R---E-		KA----------S-G---G-----
	Xylella fastidiosa	15837922	---------------------K---T-		GSIITT------S-A---------
Other γ-Proteobacteria	Aeromonas hydrophila	117618772	-------------L-I-A--SRR--R-	E	DPIKEH------C-T--NR-D---
	Alcanivorax borkumensis	110833980	---------------I-A---RH--Q-	D	G-I--R------S-E---N-Q---
	Allochromatium vinosum	288940165	------------ML-I-S-RSKR--EN	L	DPIREY------D-M--AN-----
	Alteromonadales bacterium	119470940	-------------L-I-H--SKR--E-	L	ENIKEH------N-E--AK-----
	Azotobacter vinelandii	226945563	------------ML---A--SRR--N-	E	EPIKEH------S-D--NK----G
	Cardiobacterium hominis	258543901	-------------L-I-A--S-R--L-	E	DP-KEH-VI---N-E--QQQ----
	Cellvibrio japonicus	192360095	-------------L-I-Q--SRR--QS	Q	EPIREH------N-S---A-----
	Citrobacter koseri	157145438	-------------L-I-A--SRR--N-	E	DPIKEH------N-G--NR-D---
	Colwellia psychrerythraea	71279322	-------------L-M-A-RSRR--L-	L	EPIKEH------S-K---E-----
	Dickeya dadantii	271500524	-------------L-I-S--SRR--Q-	Q	DPIKEH------N-G--SR-D---
	Enterobacter cancerogenus	261340186	-------------L-I-A--SRR--N-	Q	EPIKEH------N-G--SK-D---
	Erwinia tasmaniensis	188533678	-------------L-I-S--SRR--N-	Q	DAIKEH------N-G--NR-D---
	Escherichia coli	191169112	-------------L-I-A--SRR--N-	E	EPIKEH------N-G--SR-D---
	Grimontia hollisae	262276191	-------------L-I----SRR--N-	E	EP-KTH------N-G--AR-----
	Hahella chejuensis	83644579	-------------L-I-H--SRR--M-	L	DP-KEH------N-E--QN-----
	Marinobacter algicola	149377096	-------------L-I-Q--SRR--M-	Q	DP-KEH---S--N-D---K-----
	Nitrococcus mobilis	88813343	------------VL---A---RR--R-	E	PS-RER--V---A-S--GR-----
	Photobacterium profundum	90411625	-------------L-I----SRR--QA	E	EP-KQH------N-A--TN-D---
	Providencia alcalifaciens	212711899	-------------L-I-S--SRR--K-	Q	DPIKEH------N-G--TR-D---
	Pseudomonas aeruginosa	152987775	------------ML---A--SQR--K-	E	EAIKEH------N-E--TK----G
	Salmonella enterica	16760705	-------------L-I-A--SRR--N-	E	EPIKEY------N-G--NK-D---
	Shewanella amazonensis	119775066	-------------L-I-Q--SRR--QN	L	EPIKEY------S-T--KN-----
	Vibrio cholerae	229529016	-------------L-I----SMR--Q-	Q	APIKQH------N-A--TQ-----
	Yersinia intermedia	238794478	-------------L-I-S--SRR--N-	Q	DPIKEH------N-G--NR-D---
β-Proteobacteria	Kingella kingae	333375644	-------------L-I-Q--SL---M-		-S-KEH--I---S-E---K-----
	Eikenella corrodens	225025768	-------------L-I-Q--SRH--K-		EQ-KEH--I---N-E---------
	Cupriavidus metallidurans	94308979	-------------L-I-S---KR-SE-		EPIKEH--I---N-K--HG-----
	Neisseria meningitidis	15676098	-------------L-I-Q--S----Q-		GS-KEH--I---S-E--AK-----
	Comamonas testosteroni	221069679	-------------L-M-S---ER-VK-		ESIKEH--I---N-N---D-Q---
	Delftia acidovorans	160895542	-------------L-M-S---AR-IA-		ES-KEH--I---N-N--QD-Q---
	Ralstonia eutropha	73539769	-------------L-I-A---KR-SE-	G	EPIKEH--I---N-K--HG-----
	Achromobacter xylosoxidans	338780958	-------------L-I-AA-SKR-VE-	G	DP-KE-------N-K--VD-----
	Acidovorax sp. JS42	121592501	-------------L-M-S---KR-IE-	G	EP-KEH--I---N-H---D-Q---
	Pusillimonas sp. T7-7	332285270	-------------L-I-SA-SRR--K-	E	EPIKEY------N-K--AE-----
	Acidovorax delafieldii	241764905	-------------L-M-G---KR-IE-	G	EPIKEH--I---N-S---D-Q---
	Oxalobacteraceae bacterium	329901598	-------------L-IIQA-SRR-QN-	G	EP-KEH--I---V-K---------
	Burkholderia graminis	170691957	-------------L-I-S---KR-IE-	K	EPIKEH--I---N-K--SE-----
α-Proteobacteria	Ochrobactrum anthropi	153010294	------------------A--L---R-		ER-EKH------D---A-R-D--K
	Methylocella silvestris	217976820	---------------------E---K-		ERMEKH------DAA-A-R----K
	Gluconobacter oxydans	58038876	---------------------Q---Q-		EK-EKH------D-A-AARK----
	Brucella neotomae	256059452	---------------------L---R-		EKMDKH------D-S-A-R-D--K
	Methylobacterium nodulans	220922834	------------------A--AR--A-		-SLDKH-I----D-A-A-R----K
	Sinorhizobium medicae	150376483	------------------A--ER--R-		ER-EKH------DA--A-R-D--K
	Aurantimonas manganoxydans	90419084	------------------A--A---R-		ERMEKH------D-N-A-R-D--K

Figure S41

Partial sequence alignment of a conserved region of the Septum-site determining protein MinD showing a 1 aa deletion that is present in all Xanthomonadales. This CSI is present in some species from β-Proteobacteria.
