# Supplementary material for: Phylogenomics and Molecular Signatures for Species from the Plant Pathogen-Containing Order Xanthomonadales
Source: PLoS One. 2013 Feb 8;8(2):e55216. doi: 10.1371/journal.pone.0055216 (PMC3568101; doi:10.1371/journal.pone.0055216)
Supplement: Figure S42 — A Neighbor-joining tree based upon sequences from hypothetical protein X001065. The Tree is showing the Xanthomonadales and various β-Proteobacteria that share the 1 aa deletion. Species representing some other Gammaproteobacteria are also shown in tree. (PDF) [file pone.0055216.s042.pdf]

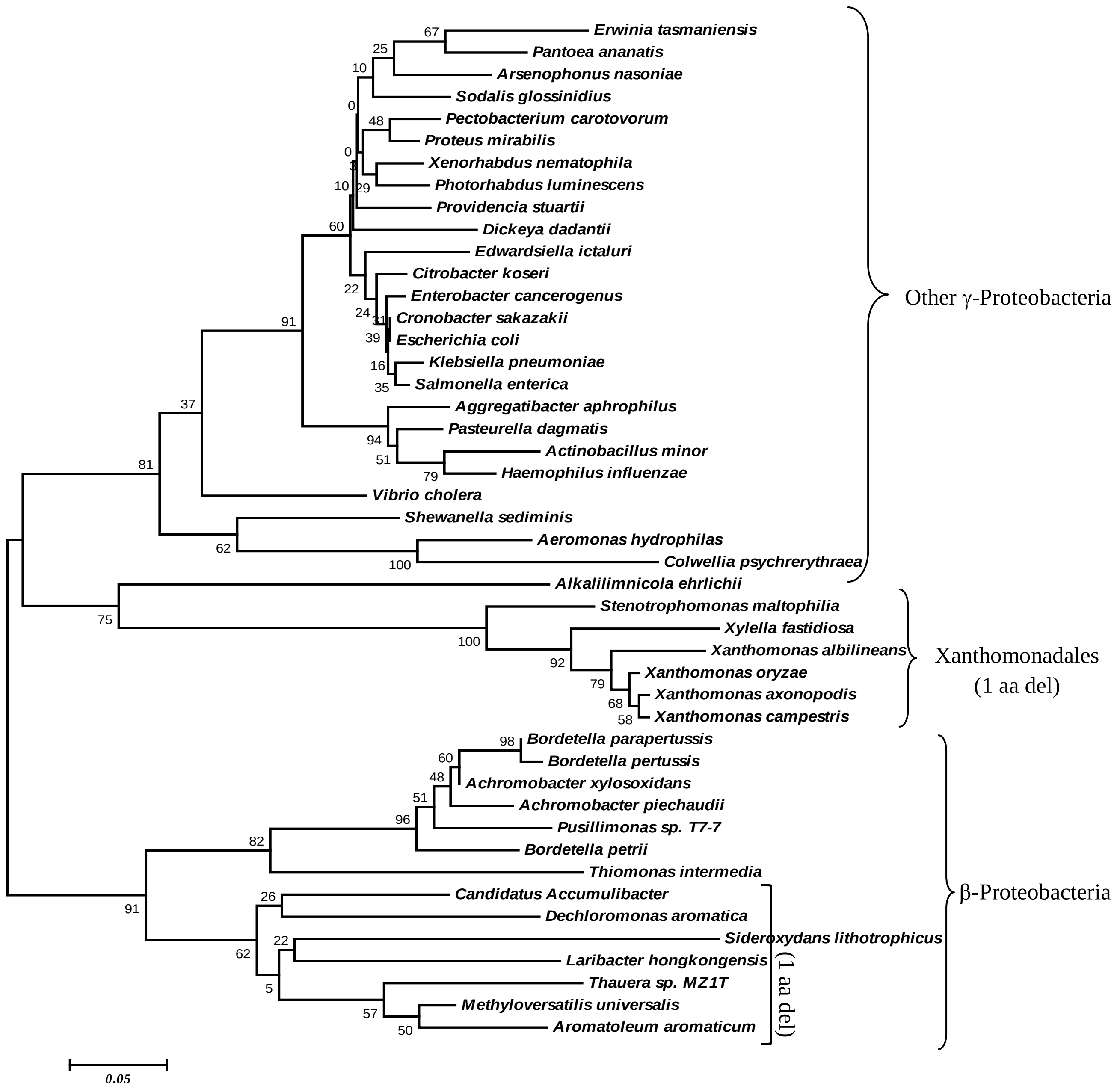

Figure S42

A Neighbor-joining (NJ) tree based upon sequences from hypothetical protein X001065. The numbers on the nodes indicate the bootstrap values. The Tree is showing the Xanthomonadales and various β-Proteobacteria that share the 1 aa deletion. Species representing some other Gammaproteobacteria are also shown in tree.
