# Supplementary material for: Phylogenomics and Molecular Signatures for Species from the Plant Pathogen-Containing Order Xanthomonadales
Source: PLoS One. 2013 Feb 8;8(2):e55216. doi: 10.1371/journal.pone.0055216 (PMC3568101; doi:10.1371/journal.pone.0055216)
Supplement: Figure S43 — A Neighbor-joining tree for Proteobacterial species based upon orotate phosphoribosyltransferase sequences. The Xanthomonadales and different β-Proteobacteria that contain the 1 aa deletion in this protein do not branch together in this tree suggesting that the deletion in these two groups have likely occurred independently. The tree shows only representative species from other Gammaproteobacteria and Alphaproteobacteria and it was rooted using sequences from Epsilonproteobacteria. (PDF) [file pone.0055216.s043.pdf]

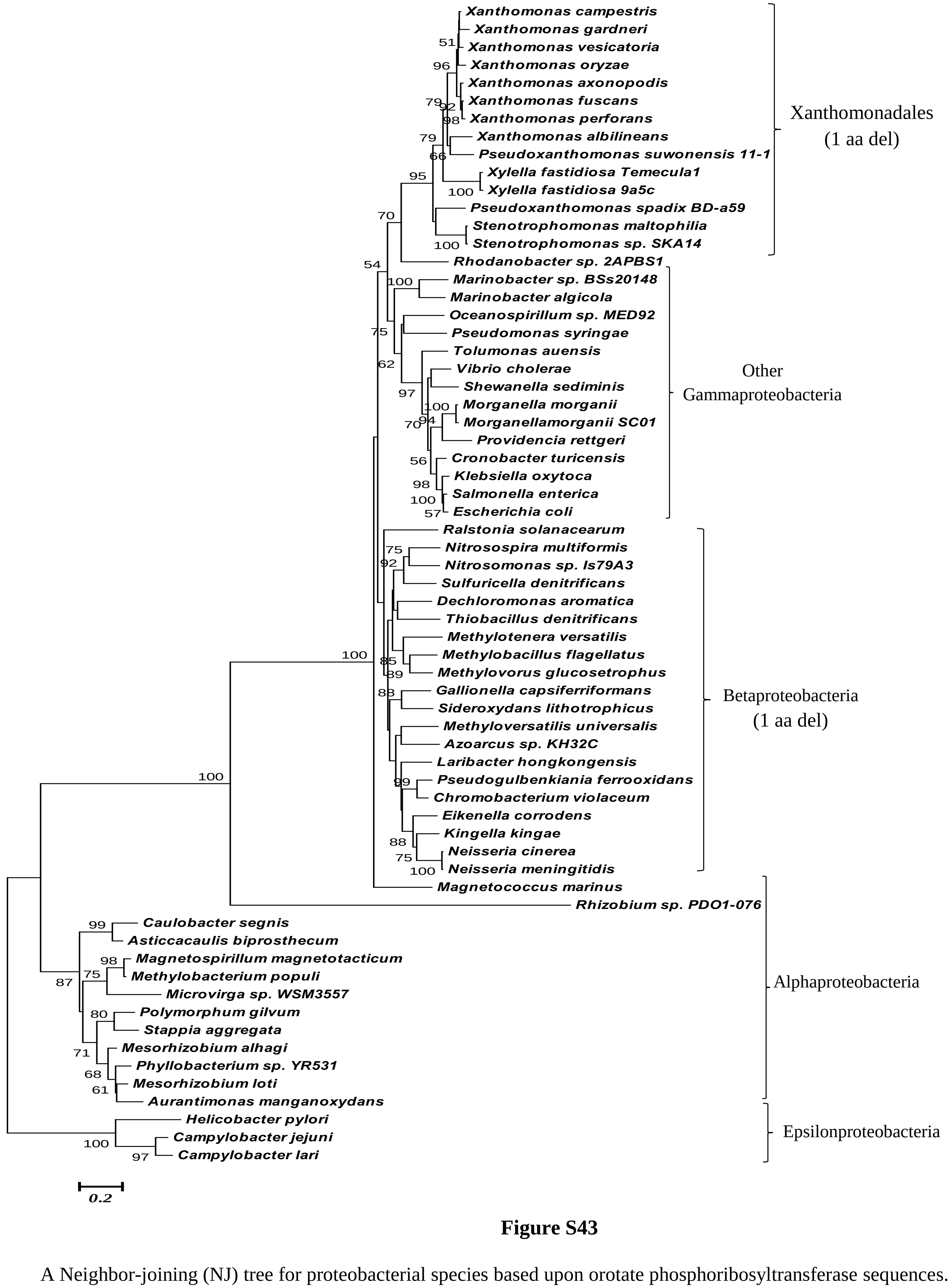

Figure S43

A Neighbor-joining (NJ) tree for proteobacterial species based upon orotate phosphoribosyltransferase sequences. The numbers on the nodes indicate the bootstrap values. The Xanthomonadales and different β-Proteobacteria that contain the 1 aa deletion in this protein do not branch together in this tree suggesting that the deletion in these two groups have likely occurred independently. The tree shows only representative species from other Gammaproteobacteria and Alphaproteobacteria and it was rooted using sequences from Epsilonproteobacteria.
