# Supplementary material for: Phylogenomics and Molecular Signatures for Species from the Plant Pathogen-Containing Order Xanthomonadales
Source: PLoS One. 2013 Feb 8;8(2):e55216. doi: 10.1371/journal.pone.0055216 (PMC3568101; doi:10.1371/journal.pone.0055216)
Supplement: Figure S45 — A maximum-likelihood tree based upon sequences from glycyl-tRNA synthetase subunit beta. The Tree shows the branching of Xanthomonadales separately from the other insert containing Betaproteobacteria. The species distribution of this insert could be explained by either the independent occurrence of a similar genetic event in the Betaproteobacteria and the Xanthomonadales, or that this insert was introduced in a common ancestor of the Beta- and Gamma-proteobacteria, followed by its loss from other Gammaproteobacteria after the divergence of deep-branching Xanthomonadales. (PDF) [file pone.0055216.s045.pdf]

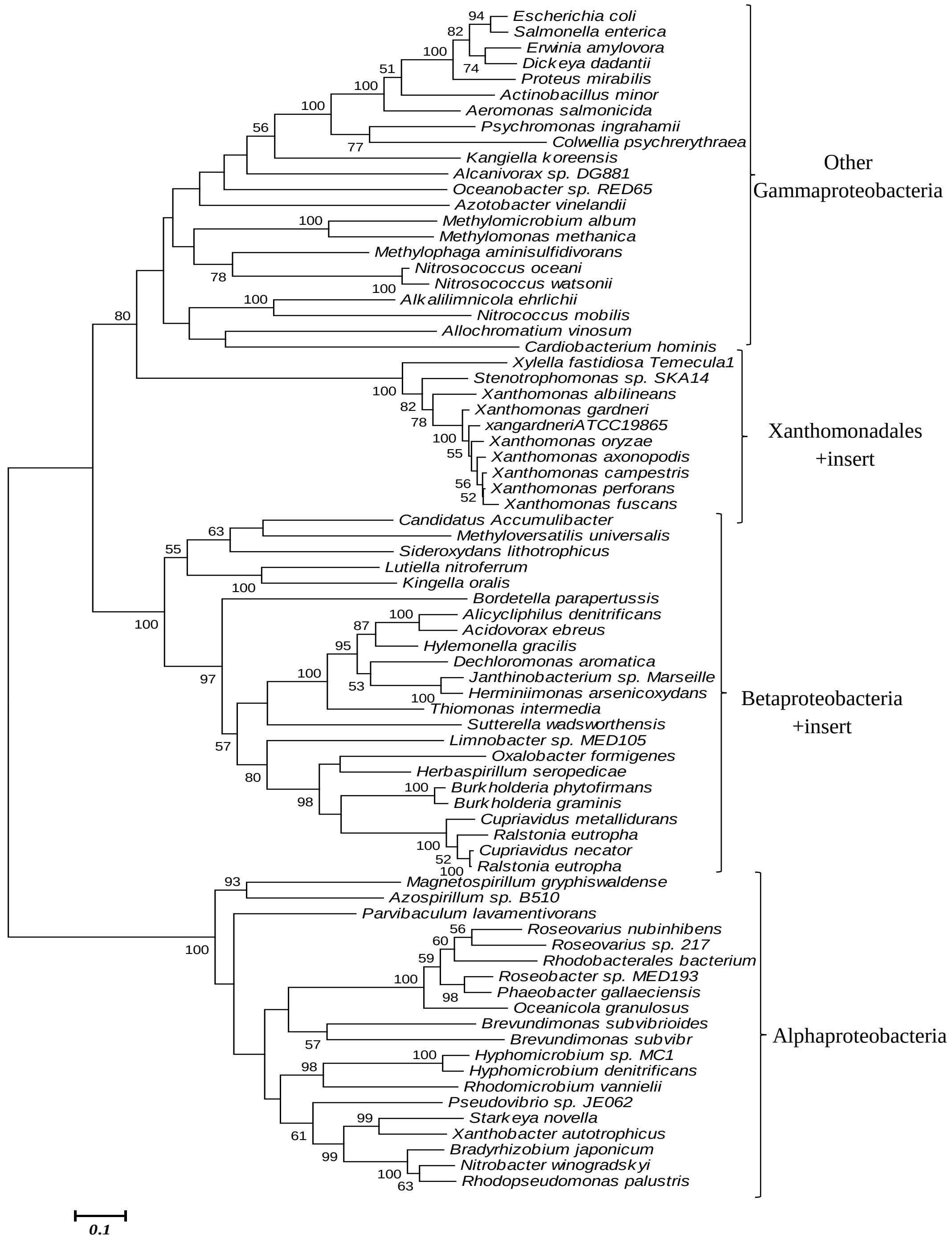

Figure S45

A maximum-likelihood (ML) tree based upon sequences from glycyl-tRNA synthetase subunit beta subunit. The numbers on the nodes indicate the bootstrap values. The Tree shows the branching of Xanthomonadales separately from the other insert containing Betaproteobacteria. The species distribution of this insert could be explained by either the independent occurrence of a similar genetic event in the Betaproteobacteria and the Xanthomonadales, or that this insert was introduced in a common ancestor of the Beta- and Gamma-proteobacteria, followed by its loss from other Gammaproteobacteria after the divergence of deep-branching Xanthomonadales.
