# Supplementary material for: Phylogenomics and Molecular Signatures for Species from the Plant Pathogen-Containing Order Xanthomonadales
Source: PLoS One. 2013 Feb 8;8(2):e55216. doi: 10.1371/journal.pone.0055216 (PMC3568101; doi:10.1371/journal.pone.0055216)
Supplement: Figure S46 — A Neighbor-joining tree based upon sequences from septum site-determining protein MinD protein. The Tree is showing the branching of Xanthomonadales distinctly from the other insert containing Betaproteobacteria. (PDF) [file pone.0055216.s046.pdf]

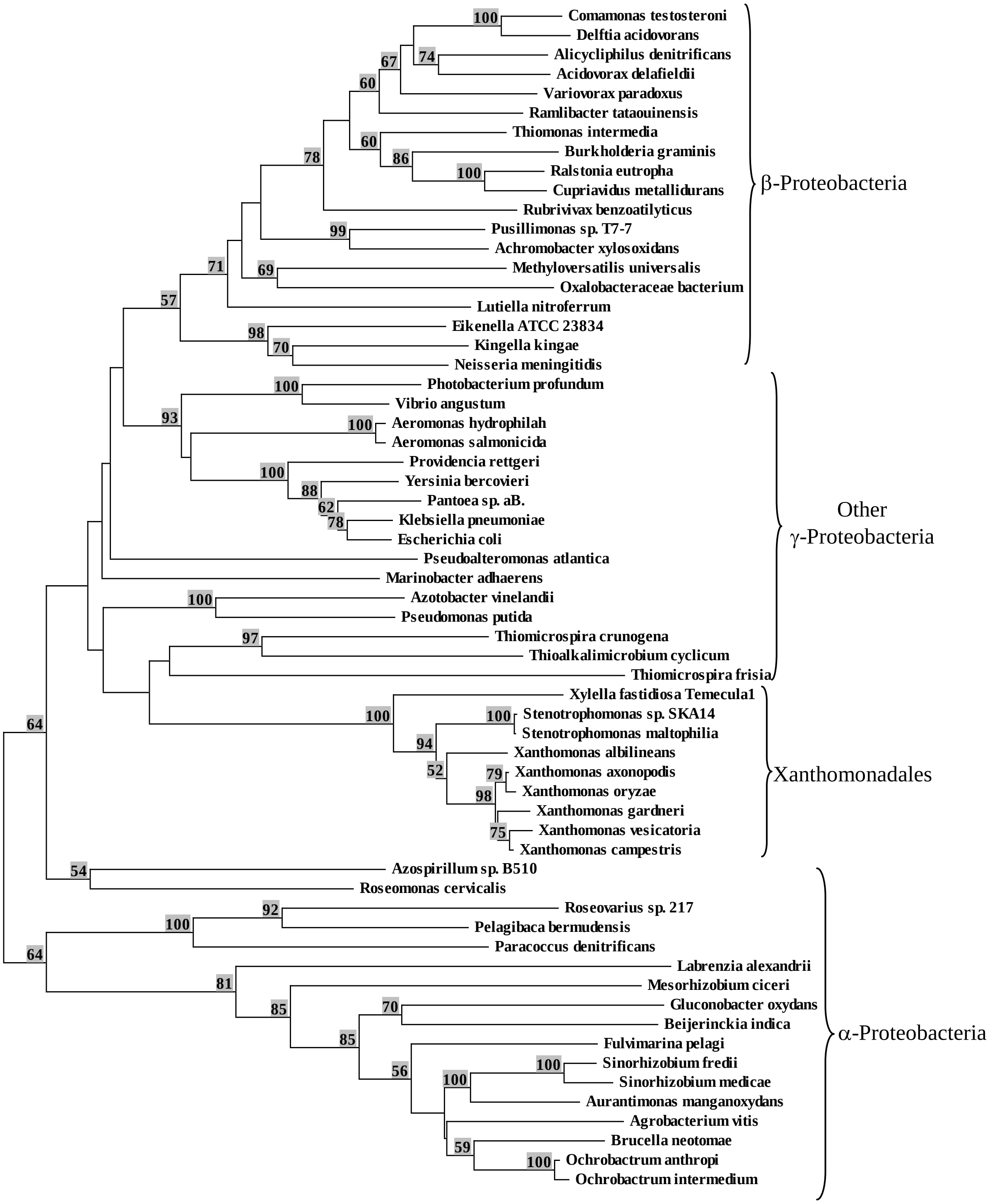


**Figure S46**

A Neighbor-joining (NJ) tree based upon sequences from septum site-determining protein MinD protein. The numbers on the nodes indicate the bootstrap values. The Tree is showing the branching of Xanthomonadales distinctly from the other insert containing betaproteobacteria.
